# Neuronal expression of bromodomain proteins alters neuronal health and peripheral protein homeostasis to promote longevity

**DOI:** 10.1101/gad.353559.125

**Published:** 2026-09-01

**Authors:** Naibedya Dutta, Daniel Hicks, Edgar Esparza, Anna Entin, Priyadharshini Vijayakumar, Caitlin M. Lange, Tripti Nair, Elijah A. Roditi, Athena Alcala, Maxim Averbukh, Matthew Vega, Rebecca Aviles Barahona, Caitlin McTygue, Veronica Kuo, Juri Kim, Herbert Anson, Aeowynn J. Coakley, George Wang, Steven E. Pilley, Max A. Thorwald, Whitaker Cohn, Sean P. Curran, Nicolas Brouilly, Caroline Kumsta, Rachel N. Arey, Peter J. Mullen, Gilberto Garcia, Ryo Higuchi-Sanabria

**Affiliations:** 1 Leonard Davis School of Gerontology, University of Southern California, Los Angeles, California 90089, USA;; 2 Department of Immunology and Immune Therapeutics, Keck School of Medicine, University of Southern California, Los Angeles, California 90033, USA;; 3 Center for Precision Environmental Health, Department of Molecular and Cellular Biology, Baylor College of Medicine, Houston, Texas 77030, USA;; 4 Center for Cardiovascular and Muscular Diseases, Sanford Burnham Prebys Medical Discovery Institute, La Jolla, California 92037, USA;; 5 Graduate School of Biomedical Sciences, Sanford Burnham Prebys Medical Discovery Institute, La Jolla, California 92037, USA;; 6 Alfred E. Mann School of Pharmacy and Pharmaceutical Sciences, University of Southern California, Los Angeles, California 90033, USA;; 7 Centre National de la Recherche Scientifique (CNRS), Institut de Biologie du Développement de Marseille (IBDM), Aix Marseille University, Marseille 13009, France;; 8 Norris Comprehensive Cancer Center, Keck School of Medicine, University of Southern California, Los Angeles, California 90033, USA

**Keywords:** longevity, neurons, nonautonomous signaling, proteostasis, stress

## Abstract

Longevity and stress resilience require precise coordination of gene expression programs across tissues. Here, we demonstrate that overexpression of the chromatin reader *bet-1* specifically in neurons of *Caenorhabditis elegans* promotes organismal longevity and stress resistance via cell-nonautonomous signaling. Neuronal *bet-1* elicits a neurotransmitter-dependent signal that activates the conserved stress-responsive transcription factor HSF-1 in the intestine, enhancing proteostasis, oxidative stress resistance, metabolic remodeling, and immune defense. Life span extension by neuronal *bet-1* requires both *hsf-1* and *daf-16* in neurons but only requires *hsf-1* in peripheral tissues. Using bulk RNA sequencing, we reveal distinct prolongevity pathways that include enhanced heat-shock response, proteostasis, increased actin stability, and resistance to pathogens, which likely together coordinate the prolongevity effects of neuronal *bet-1*. Our findings establish BET-1 as a potent nonautonomous regulator of aging and stress response, highlighting chromatin readers as upstream modulators of intertissue signaling and systemic resilience.

## Abstract (precis)

In this study, Dutta et al. show that the neuronal expression of the chromatin reader BET-1 promotes longevity and resilience in *C. elegans*. Neuronal BET-1 activates various prolongevity transcriptional programs—including proteostasis, immune response to pathogens, and metabolism—through signaling mechanisms involving master transcription factors HSF-1 and DAF-16 and neurotransmitter UNC-13.

The rate of aging processes is often encoded genetically, and sometimes, even single genes can influence entire networks of signaling to influence longevity. Often referred to as “master regulators,” these generally include transcriptional regulators that can alter the expression of many genes that can impact age-associated processes. For example, several biological hallmarks of aging such as protein misfolding, oxidative damage, and mitochondrial dysfunction are driven by discrete stress responsive factors that counteract damage to maintain homeostasis. Heat and other proteotoxic stressors can activate the heat shock response (HSR), which is coordinated by HSF-1, a transcription factor conserved from invertebrates to humans (Liu et al. 1997; Morley and Morimoto 2004). Under conditions of stress, HSF-1 is released from inhibitory control by small heat shock proteins, trimerizes, enters the nucleus, and drives transcription of major families of genes including chaperones, autophagy, and the ubiquitin proteasome system to restore protein homeostasis (proteostasis) (Morimoto 2008). Similarly, unfolded protein responses (UPRs) exist with their own discrete induction mechanisms to restore homeostasis of cellular components, including the mitochondria (UPR^mt^) and the endoplasmic reticulum (UPR^ER^) (Yoshida et al. 2001; Sutandy et al. 2023). The robustness of HSF-1 signaling and other stress responses often declines with age, thereby reducing the cell's capacity to handle proteotoxic insults and contributing to the progression of age-related dysfunction (Ben-Zvi et al. 2009; Dues et al. 2016; Jurivich et al. 2020; Trivedi et al. 2021). Therefore, extending transcriptional responsiveness to stress presents a significant therapeutic potential to promote healthy aging and longevity (Dutta et al. 2022).

Activation of cellular stress responses can occur in a cell-autonomous manner whereby cells directly experiencing stress activate specific transcriptional programs. In addition, neuroendocrine signaling can nonautonomously promote the activation of cellular stress responses across distal tissues to alter longevity. Although neural cells are the most prominent players in this cell-nonautonomous stress response, other cell types including the germline, intestine, hypodermis, and muscle have also been shown to participate in nonautonomous stress paradigms (Nussbaum-Krammer and Morimoto 2014; Taylor et al. 2014; Doyle et al. 2020; Calculli et al. 2021; Levi-Ferber et al. 2021). For example, *hsf-1* overexpression in neurons drives a serotonin- and thermosensory circuit-dependent signal to increase HSF-1 activity throughout the entire organism, ultimately extending life span (Douglas et al. 2015). Similarly, overexpression of *hsf-1* in glia extends life span, although this is not dependent on serotonin signaling (Gildea et al. 2022). At least for neurons, this beneficial communication relies on peripheral activation of the FOXO family transcription factor, DAF-16, recapitulating work on mutants of the insulin/IGF-1 receptor homolog, *daf-2* (Gems et al. 1998; Holzenberger et al. 2003). In fact, disrupting the insulin/IGF-1 signaling pathway has produced some of the longest life span extensions in both invertebrates and mammals (Kenyon 2011).

Considering the dual-activation of DAF-16 and HSF-1 in neural *hsf-1* overexpression paradigms, it is of interest to know whether other known functions of general HSF-1 signaling also require DAF-16. HSF-1 not only acts on chaperones and other proteostasis machinery, but also has roles in maintaining mitochondrial health, promoting healthy metabolism, and maintaining cytoskeletal integrity (independent of activating canonical heat shock proteins (Baird et al. 2014; Ma et al. 2015; Pomatto et al. 2017; Qiao et al. 2017; Sural et al. 2020)). Some of this functionality for HSF-1 likely depends on FOXO3, first identified as DAF-16 in *Caenorhabditis elegans*. HSF-1 can directly bind to an intronic enhancer in *FOXO3*, and this binding is increased in a *FOXO3* polymorphism that is strongly associated with human longevity. Both transcription factors are frequently required in life span-extended animal models, where FOXO3 itself is known for promoting lipid β-oxidation and antioxidant synthesis. Maintenance of lipid oxidation helps support positive health outcomes in obesity models, such as protecting against cardiovascular dysfunction and accumulation of damaged mitochondria in the heart (Shao et al. 2020).

In our most recent work, we identified BET-1/BRD4, previously known for its role in maintaining stable gene transcription and cell identities, as an important player in stress resilience, longevity, and healthspan (Garcia et al. 2023). Epigenetic dysregulation is a hallmark of aging, which we also suspected *bet-1* might impact, as it is a reader of acetylated histone tags, opening the chromatin at these sites, as well as adding additional histone tags (Shibata et al. 2010; Zhang et al. 2012; Fisher et al. 2013; Pomatto and Davies 2017; Pomatto et al. 2017; Yang et al. 2023; Zavala et al. 2024). We found that overexpression of *bet-1* promoted longevity, in part due to an increase in actin stability and stress resilience. Here, we questioned whether BET-1 signaling could also be initiated across cell types in a cell-nonautonomous manner, similar to other stress response paradigms. By overexpressing *bet-1* solely in neurons, we achieved a dramatic life span extension beyond what was previously seen in whole-animal overexpression. This life span extension involves a complex, multisignal activation of HSF-1, DAF-16, and BET-1 distinctly in neurons and peripheral cells to promote several anti-aging pathways including protein homeostasis, immune response, and actin stability.

## Results

### Neuronal *bet-1* promotes longevity through transcriptional activation of prolongevity pathways

**Figure 1.**
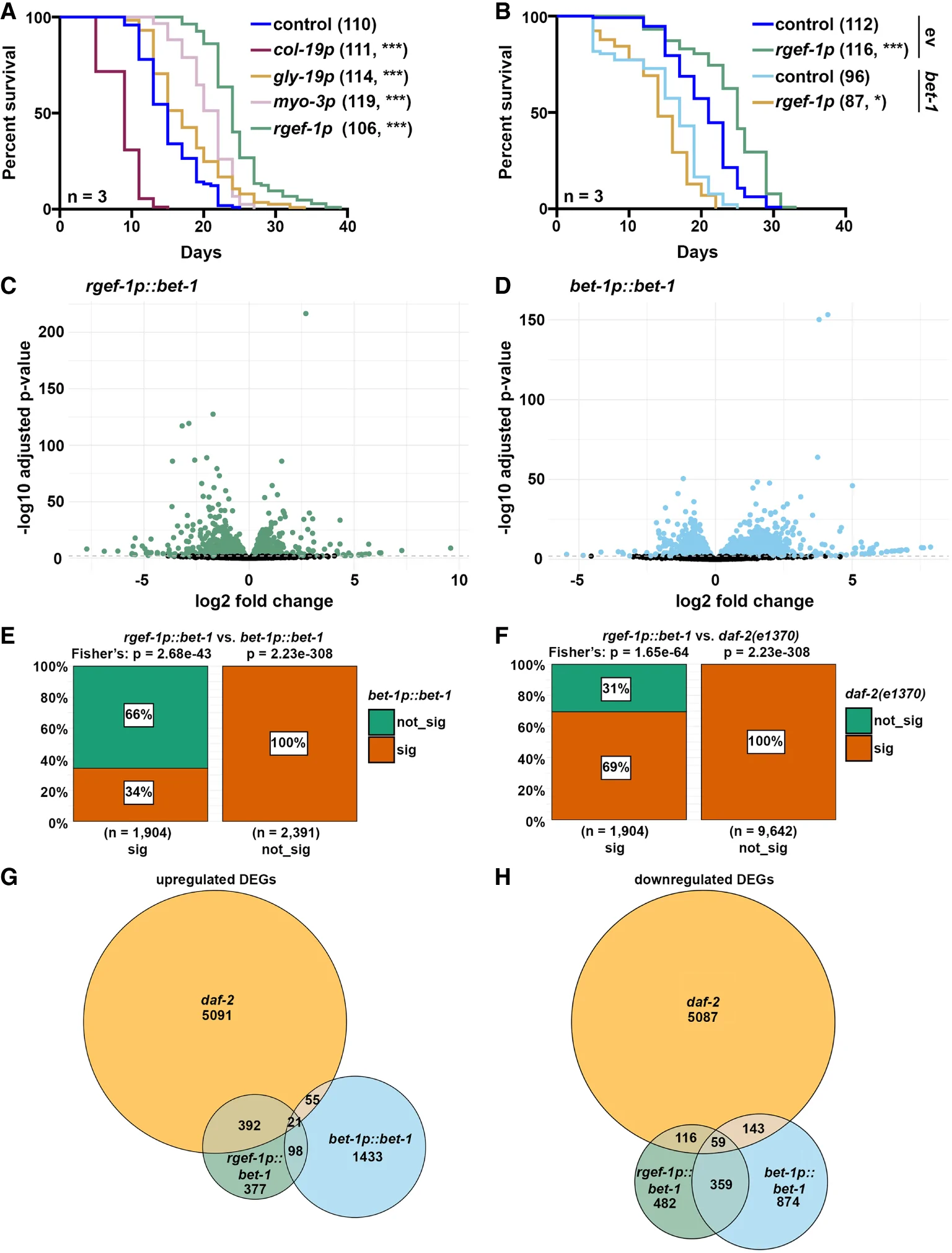
Neuronal *bet-1* extends life span through transcriptional activation of prolongevity pathways. (*A*) Life spans of N2 control (blue) and *bet-1B* overexpression in the hypodermis (*col-19p*, magenta), intestine (*gly-19p*; yellow), muscle (*myo-3p*; pink), and neurons (*rgef-1p*; green) grown on empty vector (EV) from L1. (***) *P* < 0.001, by log rank testing. See Supplemental Table S1 for complete life span statistics. Intestine-, hypodermis-, and muscle-specific life spans have been previously published (Garcia et al. 2023) and are adapted here for ease of comparison. (*B*) Life spans of N2 control (blue) and neuronal *bet-1* (*rgef-1p*; green) grown on EV or *bet-1* RNAi (light blue, yellow) from L1. (***) *P* < 0.001, (*) *P* < 0.05 by log rank testing. See Supplemental Table S1 for complete life span statistics. (*C*) RNA-seq was performed in day 1 animals in *glp-4(bn2)* animals grown at 22°C to eliminate contamination from progeny and germline. Volcano plot showing all differentially expressed genes in green and unchanged genes in black for neuronal *bet-1* (*rgef-1p::bet-1*). (*D*) Volcano plot showing all differentially expressed genes in blue and unchanged genes in black for whole-animal *bet-1* (*bet-1p::bet-1*). All differentially expressed genes are in Supplemental Table S2. (*E*) Bar plot displaying the overlap of all significant DEGs between neuronal *bet-1* (*rgef-1p::bet-1*) and whole-animal *bet-1* (*bet-1p::bet-1*) animals in the *glp-4(bn2)* background grown on EV at 22°C. Statistical significance of overlap was assessed using Fisher's exact test. (*F*) Bar plot displaying the overlap of all significant DEGs between neuronal *bet-1* (*rgef-1p::bet-1)* grown on EV versus a previously published *daf-2(e1370)* mutant data set (Son et al. 2017). Statistical significance of overlap was assessed using Fisher's exact test. (*G*) Venn diagram of overlapping upregulated DEGs in whole-animal *bet-1* (*bet-1p::bet-1*), neuronal *bet-1* (*rgef-1p::bet-1*), and *daf-2(e1370)* animals. (*H*) Venn diagram of overlapping downregulated DEGs in whole-animal *bet-1* (*bet-1p::bet-1*), neuronal *bet-1* (*rgef-1p::bet-1)*, and *daf-2(e1370)* animals.

In our previous work, *bet-1* isoform B-specific overexpression from its endogenous promoter (referred to here as *bet-1* overexpression for simplicity) extended life span by promoting actin stability and stress resilience (Garcia et al. 2023). Moreover, *bet-1* overexpression specifically in the intestine and muscle is sufficient to extend life span, but overexpression in the hypodermis significantly reduces life span (Garcia et al. 2023). Here, we expanded our analysis to determine the effect of *bet-1* overexpression specifically in neurons using the *rgef-1* promoter (referred to here as neuronal *bet-1*) (Chen et al. 2011). To our surprise, overexpressing *bet-1* in neurons resulted in a profound increase in life span greater than that of any of the tested tissue-specific overexpression strains (Fig. 1A). This life span extension is dependent on *bet-1*, as RNAi knockdown of *bet-1* suppressed this extension (Fig. 1B).

To determine whether neuronal *bet-1* animals exhibited improved healthspan metrics in addition to life span extension, we next measured several metrics of organismal health including reproductive health and locomotor behavior. We saw no significant change in reproductive health as measured by a viable brood assay (Castro Torres et al. 2022) in neuronal *bet-1* animals compared to wild-type animals (Supplemental Fig. S1A). However, we did see a mild, but statistically significant, decrease in locomotor behavior in mid-age and old animals, measured as a reduction in liquid thrashing at day 5 and day 9 of adulthood (Supplemental Fig. S1B).

Next, to get a mechanistic understanding of the life span extension of neuronal *bet-1* animals, we performed transcriptomic analysis through bulk, whole-animal RNA sequencing (RNA-seq). Importantly, we compared neuronal *bet-1* transcriptomes to whole-animal *bet-1* overexpression to determine the similarities and differences between whole-animal and neuron-specific *bet-1* overexpression. Here, we utilized *glp-4(bn2)* animals to avoid contamination from germline as whole-animal *bet-1* overexpression has been shown to significantly reduce brood size (Garcia et al. 2023) and thus may have confounding effects on transcriptome. We have confirmed that neuronal *bet-1* still extends life span in the *glp-4(bn2)* background at the restrictive temperature of 22°C (Supplemental Fig. S2A). RNA-seq libraries showed Phred scores >35 with consistent mapping and clustering across replicates, indicating high-quality data (Supplemental Fig. S2B–E). As expected with overexpression of a major transcriptional regulator, we found several thousand differentially expressed genes when overexpressing *bet-1* in neurons (Fig. 1C) or throughout the entire animal (Fig. 1D). Interestingly, we found that more than half (∼66%) of differentially expressed genes in neuronal *bet-1* animals were distinct from those of whole-animal *bet-1* animals (Fig. 1E,G,H), suggesting that neuronal *bet-1* animals, while having some overlap with whole-animal *bet-1* animals, drive different mechanistic outcomes. As previously reported, whole-animal *bet-1* overexpression promotes longevity through increased stress resilience to ER, mitochondrial, and oxidative stress, as well as improved actin stability (Garcia et al. 2023). Neuronal *bet-1* and whole-animal *bet-1* animals shared changes in oxidative stress response genes (Supplemental Fig. S2F); however, we did not observe any statistical significance in differentially expressed genes for UPR^MT^ or UPR^ER^ in whole-animal *bet-1* animals, although UPR^ER^ displayed significant differences in DEGs in neuronal *bet-1* animals (Supplemental Fig. S2G,H). Both neuronal *bet-1* and whole-animal *bet-1* animals had altered expression of translation-related genes (Supplemental Fig. S2I). It is important to note that neuronal *bet-1* animals have a significantly higher level of *bet-1* expression compared to whole-animal *bet-1* animals (Supplemental Fig. S3A), which may contribute to these differences. Overall, these data suggest that while some overlaps exist, neuronal *bet-1* animals have distinct transcriptional changes from whole-animal *bet-1*.

To better understand the mechanisms driving neuronal *bet-1* life span extension, we performed gene ontology pathway analysis and found that neuronal *bet-1* animals showed significant enrichment of differentially expressed genes in several prolongevity pathways, including mitochondrial processes, metabolic remodeling, immune response, and protein homeostasis (Supplemental Fig. S3B,C). More importantly, we saw that more than half of the differentially expressed genes in neuronal *bet-1* animals (∼69%) overlapped with the transcriptome of the insulin/IGF-1 signaling mutant, *daf-2(e1370)*, one of the longest-lived mutants recorded in *C. elegans* (Fig. 1F–H; Kenyon et al. 1993; Zarse et al. 2012). A majority of the prolongevity pathways we identified have been directly ascribed to functions of the master transcriptional regulator, HSF-1 (Hsu et al. 2003; Morley and Morimoto 2004; Singh and Aballay 2006; Ben-Zvi et al. 2009; Minsky and Roeder 2015; Sural et al. 2020; Williams et al. 2020; Chauve et al. 2021; Erinjeri et al. 2024). Longevity associated with *hsf-1* overexpression is also dependent on the DAF-16/FOXO transcription factor, thus suggesting that neuronal *bet-1* may potentially promote longevity by activating a similar HSF-1/DAF-16 network.

**Figure 2.**
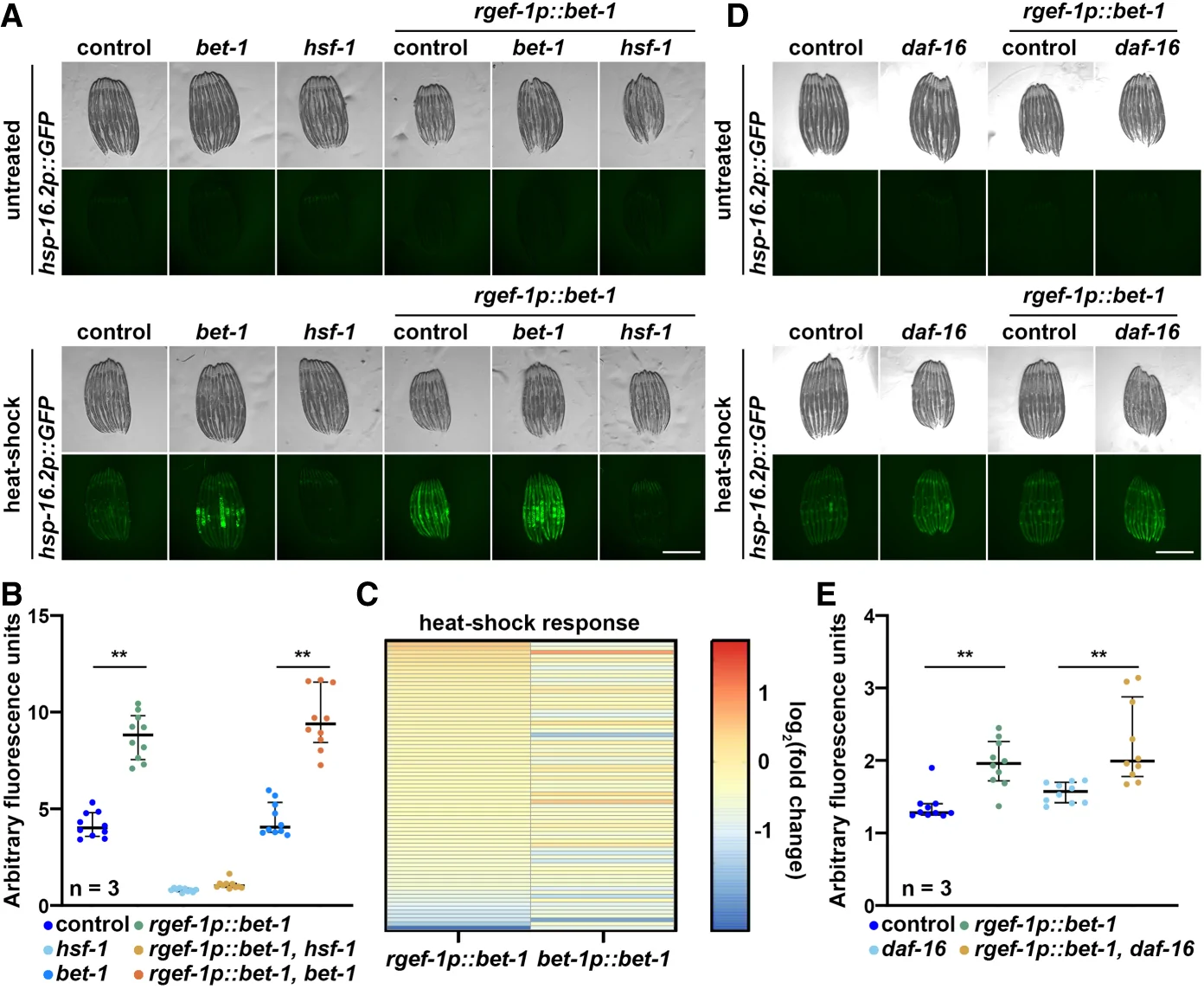
Neuronal *bet-1* promotes life span through *hsf-1* and *daf-16*. (*A*) Representative fluorescent images of day 1 adult animals expressing *hsp-16*.*2p*::*GFP* grown on EV (control), *hsf-1*, or *bet-1* RNAi bacteria from L1. Animals were heat-shocked at 34°C for 2 h followed by a 2 h recovery at 20°C. Data is representative of three independent replicates. Scale bar, 500 µm. (*B*) Quantification of *A* presented as arbitrary fluorescent units, which are integrated fluorescence intensity measurements using ImageJ Fiji. Lines represent the median and interquartile range. Quantification is only provided for heat-shocked samples. Data is representative of three independent replicates. (**) *P* < 0.01 measured by the nonparametric Mann–Whitney testing. (*C*) Heat map of heat-shock response gene expression (GO:0009408) showing *bet-1p::bet-1* and *rgef-1p::bet-1* animals compared to *glp-4(bn2)* control worms. Warmer colors indicate increased expression, and cooler colors indicate decreased expression. See Supplemental Table S4 for all raw gene counts for heat map. (*D*) Representative fluorescent images of day 1 adult animals expressing *hsp-16*.*2p*::*GFP* grown on EV or *daf-16* RNAi bacteria from L1. Animals were heat-shocked for 2 h at 34°C followed by a 2 h recovery at 20°C. Data is representative of three independent replicates. Scale bar, 500 µm. (*E*) Quantification of *D* performed similarly to *B*.

To directly test whether neuronal *bet-1* animals had increased HSF-1 signaling, we utilized the *hsp-16.2p::GFP* transcriptional reporter, which is robustly induced upon heat stress (Link et al. 1999). We found that neuronal *bet-1* almost doubled *hsp-16.2p::GFP* activation in response to heat stress, which was dependent on *hsf-1* but not *bet-1* (Fig. 2A,B). Importantly, *hsp-16.2p::GFP* expression was activated throughout the animal, adding further evidence that neuronal *bet-1* is driving peripheral HSF-1 activity. Interestingly, *bet-1* knockdown also seems to increase *hsp-16.2p::GFP* signal, although this is only apparent in the eggs, which is confounded by the fact that *bet-1* knockdown induces an egg-laying defect. More importantly, neuronal *bet-1* animals exhibited transcriptomic changes in several heat-shock response (HSR) genes, although few of these changes overlapped with whole-animal *bet-1* overexpression (Fig. 2C). This increased HSF-1 transcriptional activity was independent of *daf-16*, as RNAi knockdown of *daf-16* did not suppress the increase in *hsp-16.2p::GFP* found in neuronal *bet-1* animals (Fig. 2D,E). This is consistent with a heterotypic response to neuronal *hsf-1* that showed divergent *hsf-1* and *daf-16* dependent pathways activated in the periphery (Douglas et al. 2015).

**Figure 3.**
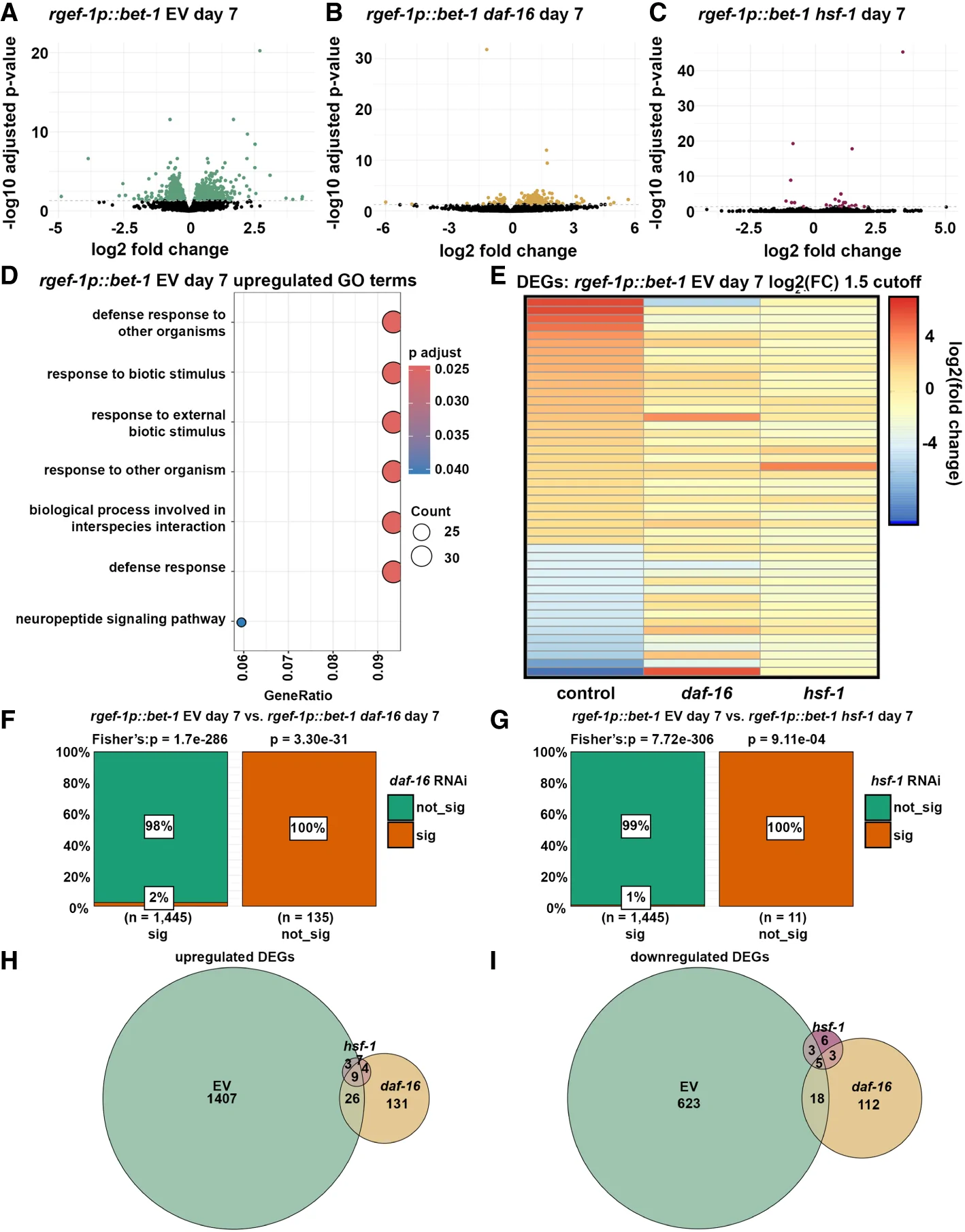
RNA-seq reveals transcriptomic changes of neuronal *bet-1* are largely *daf-16* and *hsf-1* dependent. RNA-seq was performed in day 7 animals in N2 background to avoid contamination with progeny and germ cells and potential confounding factors of growing *hsf-1* knockdown animals at elevated temperature of 22°C required for *glp-4(bn2)* animals. (*A*) Volcano plot showing all differentially expressed genes in green and unchanged genes in black for neuronal *bet-1* (*rgef-1p::bet-1*) grown on EV control. (*B*) Volcano plot showing all differentially expressed genes in yellow and unchanged genes in black for neuronal *bet-1* (*rgef-1p::bet-1*) grown on *daf-16* RNAi against an N2 control grown on *daf-16* RNAi. (*C*) Volcano plot showing all differentially expressed genes in purple and unchanged genes in black for neuronal *bet-1* (*rgef-1p::bet-1*) grown on *hsf-1* RNAi against an N2 control grown on *hsf-1* RNAi. All differentially expressed genes are in Supplemental Table S2. (*D*) Top gene ontology terms from the significantly upregulated genes (*P* < 0.05) in neuronal *bet-1* (*rgef-1p::bet-1*) grown on EV control. See Supplemental Table S3 for all GO terms. (*E*) Heat map of genes with larger than a 1.5 log_2_(fold change [FC]) in either direction (upregulated or downregulated) in neuronal *bet-1* (*rgef-1p::bet-1*) grown on EV (control) compared to N2 control grown on EV. *daf-16* represents fold changes of neuronal *bet-1* (*rgef-1p::bet-1*) grown on *daf-16* RNAi against an N2 control grown on *daf-16* RNAi. *hsf-1* represents fold changes of neuronal *bet-1* (*rgef-1p::bet-1*) grown on *hsf-1* RNAi against an N2 control grown on *hsf-1* RNAi. Warmer colors indicate increased expression and cooler colors indicate decreased expression. DEGs with log_2_(fold change) >1.5 are shown for ease of visibility. See Supplemental Table S4 for all raw gene counts for heat map. (*F*) Bar plot displaying the overlap of all significant DEGs between neuronal *bet-1* (*rgef-1p::bet-1*) grown on EV compared to N2 control grown on EV versus neuronal *bet-1* (*rgef-1p::bet-1*) grown on *daf-16* RNAi compared to N2 control grown on *daf-16* RNAi. Statistical significance of overlap was assessed using Fisher's exact test. (*G*) Bar plot displaying the overlap of all significant DEGs between neuronal *bet-1* (*rgef-1p::bet-1*) grown on EV compared to N2 control grown on EV versus neuronal *bet-1* (*rgef-1p::bet-1)* grown on *hsf-1* RNAi compared to N2 control grown on *hsf-1* RNAi. Statistical significance of overlap was assessed using Fisher's exact test. (*H*) Venn diagram of overlapping upregulated DEGs in neuronal *bet-1* grown on EV, *daf-16*, or *hsf-1* RNAi. (*I*) Venn diagram of overlapping downregulated DEGs in neuronal *bet-1* grown on EV, *daf-16*, or *hsf-1* RNAi.

We next sought to determine whether the general transcriptomics signature of neuronal *bet-1* animals was dependent on downstream activation of HSF-1 and DAF-16. Therefore, we performed comparative RNA-seq analysis in the presence of *daf-16* and *hsf-1* RNAi. Previously, we used the germlineless *glp-4(bn2)* animals for RNA-seq analysis to eliminate germline. However, the requirement of growing the animals at 22°C, presented a potential challenge as *daf-16* and *hsf-1* knockdown significantly alters sensitivity to heat stress (McColl et al. 2010; Douglas et al. 2015; Dues et al. 2016). Therefore, we opted to perform our RNA-seq analysis on day 7 animals, where progeny formation had already ceased, thus eliminating the necessity to use a temperature-sensitive germlineless mutant. RNA-seq libraries showed Phred scores >35 with consistent mapping and clustering across replicates, indicating high-quality data (Supplemental Fig. S4A–D). As expected, neuronal *bet-1* animals still exhibit differential gene expression of over a thousand genes even at day 7 of adulthood (Fig. 3A), many of which are also involved in prolongevity pathways, including immune response (Fig. 3D), although not all prolongevity signatures could be identified at day 7, which was not entirely surprising considering that many pathways including proteostasis collapse by day 7 (Ben-Zvi et al. 2009). Importantly, activation of a large number of DEGs in neuronal *bet-1* animals was suppressed upon RNAi knockdown of *daf-16* (Fig. 3B) or *hsf-1* (Fig. 3C), with *hsf-1* knockdown showing more prominent effects. Extraction of the top upregulated and downregulated (i.e., log_2_(fold-change) > 1.5 or <−1.5) DEGs further highlighted that almost all of these genes were no longer differentially expressed upon *hsf-1* knockdown (Fig. 3E; all DEGs available in Supplemental Fig. S4E). Further concordance analysis revealed that <2% of DEGs found in neuronal *bet-1* animals overlapped in conditions with *daf-16* (Fig. 3F,H,I) or *hsf-1* (Fig. 3G–I) knockdown. Altogether, these data show that neuronal *bet-1* animals activate a multipronged prolongevity transcriptomics signature, dependent on downstream DAF-16 and HSF-1 signaling.

### Neuronal *bet-1* animals display improved proteostasis

**Figure 4.**
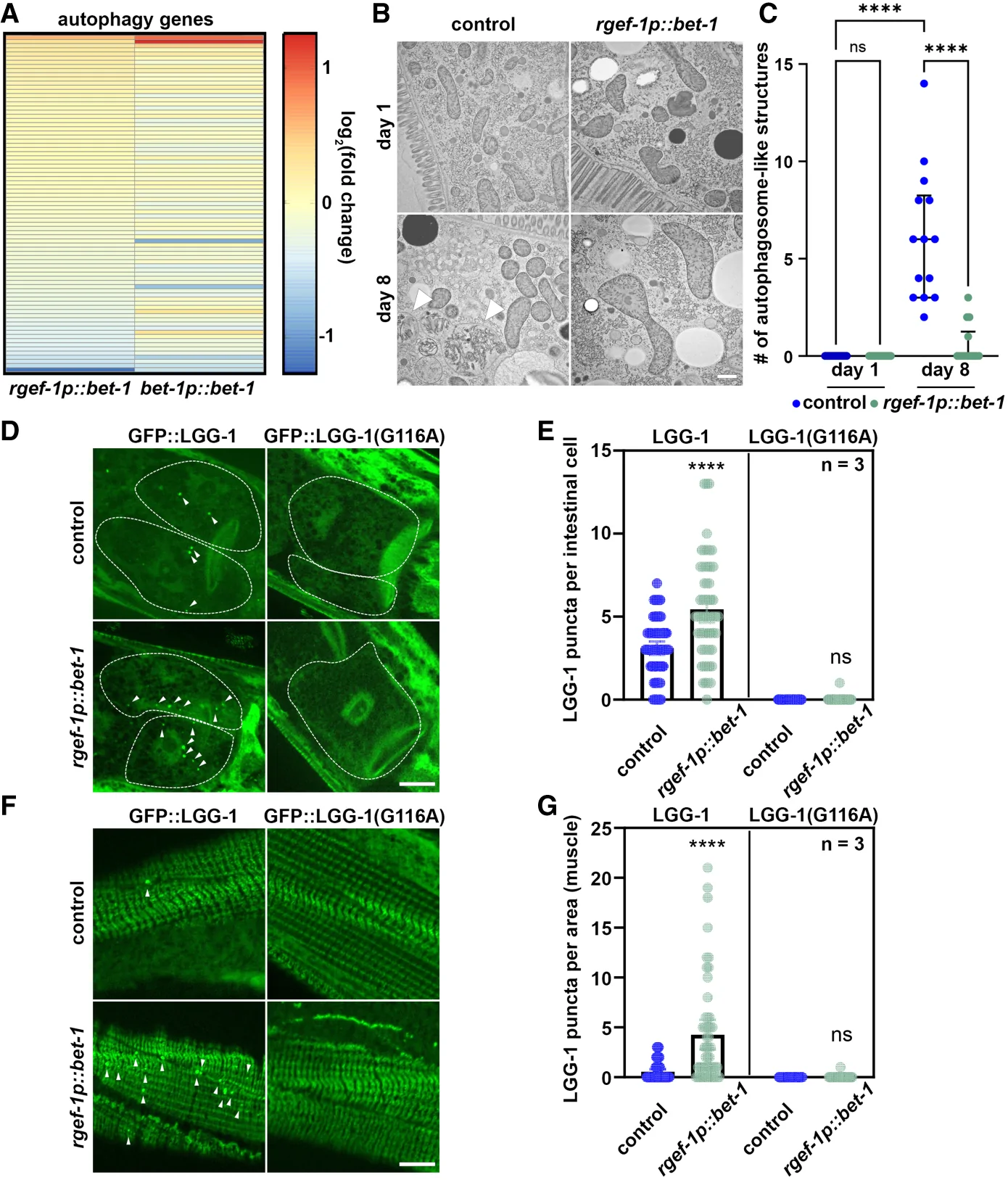
Neuronal *bet-1* animals display reduced age-associated defects in autophagy. (*A*) Heat map of autophagy gene expression (GO:0006914) showing *bet-1p::bet-1* and *rgef-1p::bet-1* animals compared to *glp-4(bn2)* control worms. Warmer colors indicate increased expression, and cooler colors indicate decreased expression. See Supplemental Table S4 for all raw gene counts for heat map. (*B*) Electron microscopy was performed in the intestine of wild-type control and neuronal *bet-1* animals grown on EV RNAi from L1 at day 1 and day 8 of adulthood. White arrowheads indicate autophagosome-like structures. Scale bar, 500 nm. (*C*) Quantification of *B* as number of autophagosome-like structures per EM image field (∼20 µm^2^ field per image). Fourteen images per sample were quantified. (*D*) Representative images of GFP::LGG-1 and GFP::LGG-1(G116A) puncta in proximal intestinal cells of wild-type and *rgef-1p::bet-1* animals on day 1 of adulthood. Dashed lines represent single intestinal cells. White arrowheads point to LGG-1::GFP puncta. Scale bar, 10 µm. (*E*) GFP::LGG-1-positive puncta were quantified from three independent experiments in proximal intestinal cells [LGG-1: *N* = 73, LGG-1(G116A): *N* = 69, LGG-1; *rgef-1p::bet-1*: *N* = 57, and LGG-1(G116A); *rgef-1p::bet-1*: *N* = 72 cells]. (*F*) Representative images of GFP::LGG-1 and GFP::LGG-1(G116A) puncta in muscle of wild-type and *rgef-1p::bet-1* animals on day 1 of adulthood. White arrowheads point to LGG-1::GFP puncta. Scale bar, 10 µm. (*G*) GFP::LGG-1-positive puncta were quantified from three independent experiments in body-wall muscle (LGG-1: *N* = 57; LGG-1(G116A): *N* = 35; LGG-1; *rgef-1p::bet-1*: *N* = 52; LGG-1(G116A); *rgef-1p::bet-1*: *N* = 36). Error bars indicate 95% CI. (ns) *P* > 0.05, (****) *P* < 0.0001, by two-way ANOVA with Šídák's multiple comparisons test. Data are shown as a superplot of three independent replicates, with each different shade of blue or green representing a single replicate.

HSF-1 and DAF-16 both drive many prolongevity pathways, including proteostasis, increased actin stability, immune response, and lipid homeostasis. Therefore, we next tested the effect of neuronal *bet-1* on these diverse prolongevity pathways. First, we evaluated the effect of neuronal *bet-1* on autophagy, as *hsf-1* overexpression has been shown to promote longevity through activation of autophagy (Kumsta et al. 2017). Although we did not see significant differences in transcriptional expression of a majority of autophagy-related genes in neuronal *bet-1* animals, a handful of genes did show differential expression (Fig. 4A). To assess whether neuronal *bet-1* influences autophagy, we used a GFP-tagged LGG-1/Atg8 reporter (Meléndez et al. 2003), which allows autophagosomes to be visualized as fluorescent puncta. Neuronal *bet-1* animals exhibited a significant increase in GFP::LGG-1 puncta in both the intestine and muscle compared to controls. To test whether these puncta represent bona fide autophagosomes, we examined GFP::LGG-1(G116A) animals (Manil-Ségalen et al. 2014), which carry a mutation that prevents LGG-1 lipidation and incorporation into autophagosomal membranes (Fig. 4D–G). Using this reporter, puncta formation was abolished in both control and neuronal *bet-1* animals, indicating that GFP::LGG-1 puncta represent bona fide autophagosomes, supporting the conclusion that neuronal *bet-1* promotes autophagy in the intestine and muscle. Importantly, unbiased electron microscopy of high-pressure frozen samples from older animals revealed a large number of autophagosome-like organelles in the control animals. These structures resembled accumulated autophagosomes that fail to be turned over by lysosomes, indicative of impaired autophagy degradation with age. This phenotype was mitigated in neuronal *bet-1* animals (Fig. 4B,C), further supporting a beneficial role for neuronal *bet-1* in autophagy by maintaining vesicle turnover with age.

During aging, protein homeostasis declines, leading to the accumulation of protein aggregates, including polyglutamine (polyQ) in *C. elegans*. These aggregates can be reduced under conditions that enhance protein clearance, including activation of autophagy mediated by the autophagy receptor, p62/SQST-1 (Kumsta et al. 2019). Therefore, we measured the impact of neuronal *bet-1* on intestinal polyQ44 (Morley et al. 2002). As expected, we found that neuronal *bet-1* reduced the number of polyQ foci at late age (Supplemental Fig. S5), suggesting that autophagy function is better maintained during aging. Therefore, to determine whether maintenance of autophagy is indeed required for the reduced numbers of polyQ foci in neuronal *bet-1* animals, we performed RNAi knockdown of several autophagy genes, including *unc-51*, required for autophagy initiation; *bec-1,* required for nucleation; *lgg-1*, required for elongation and Atg8 conjugation; *sqst-1*, required for cargo recruitment; *cup-5*, required for lysosome fusion; and *epg-5*, required for autophagosome degradation (Lange et al. 2025). Interestingly, we found that RNAi knockdown of *cup-5* and *unc-51* partially suppressed the reduction of polyQ foci in neuronal *bet-1* animals (Supplemental Fig. S5), suggesting that the maintenance of autophagy is at least partially responsible for improved protein clearance in these animals.

HSF-1's prolongevity activity is not limited to proteostasis and is also required for regulation of actin stability and function (Baird et al. 2014; Higuchi-Sanabria et al. 2018). In addition, whole-animal *bet-1* overexpression increases actin stability (Garcia et al. 2023). Therefore, we sought to determine whether neuronal *bet-1* animals similarly exhibit improved actin stability. Interestingly, neuronal *bet-1* overexpression did not result in general transcriptional activation of actin-related genes, although a handful of genes were significantly upregulated (Supplemental Fig. S6A). In addition, unlike in whole-animal *bet-1* overexpression, we did not observe improved actin stability at any age in the intestine (Supplemental Fig. S6B,C). However, neuronal *bet-1* did result in a mild, but statistically significant increase in stability of actin in the muscle at late age (Supplemental Fig. S6D,E), which is in contrast to protein aggregation phenotypes, which were improved in the intestine. As expected, the improvement in actin stability in the muscle was dependent on *hsf-1*. However, *daf-16* was not required for the increased actin stability (Supplemental Fig. S6F,G).

### Neuronal *bet-1* promotes immune response but reduces neuronal function

**Figure 5.**
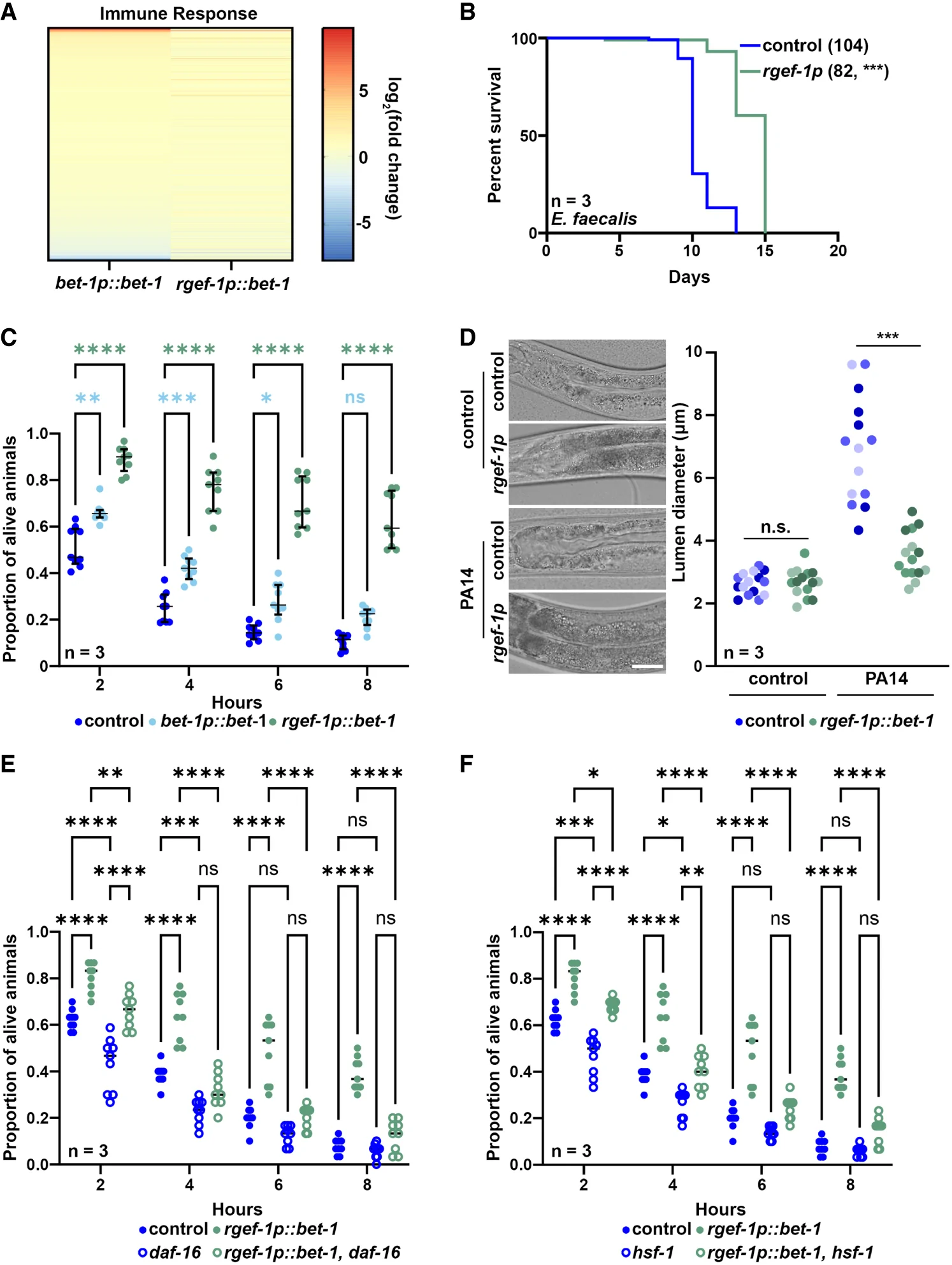
Neuronal *bet-1* animals display improved pathogen resistance. (*A*) Heat map of immune response gene expression (GO:0006955) showing *bet-1p::bet-1* and *rgef-1p::bet-1* animals compared to *glp-4(bn2)* control worms. Warmer colors indicate increased expression and cooler colors indicate decreased expression. See Supplemental Table S4 for all raw gene counts for heat map. (*B*) Survival plots of N2 control (blue) and neuronal *bet-1* (*rgef-1p::bet-1*; green) animals following exposure to *E. faecalis* starting at the L4 stage. (***) *P* < 0.001 using log rank testing. (*C*) Proportion of surviving N2 control (blue), whole-animal *bet-1* (*bet-1p::bet-1*; light blue), and neuronal *bet-1* (*rgef-1p::bet-1*; green) animals exposed to PA14 at day 1 of adulthood. Survival was scored every 2 h for 8 h. Data are pooled from three biological replicates, each of which included three technical replicates. (****) *P* < 0.0001, (***) *P* < 0.001, (**) *P* < 0.01, (*) *P* < 0.05, (ns) nonsignificant (*P* > 0.05) by two-way ANOVA. (*D*) Intestinal lumen diameter in N2 control (blue) and neuronal *bet-1* (*rgef-1p::bet-1*; green) animals at day 1 of adulthood, with a representative image of each strain shown with and without PA14 exposure. Data are pooled from three biological replicates, each containing three technical replicates. (***) *P* < 0.001, (ns) nonsignificant (*P* > 0.05) by two-way ANOVA. (*E*) Proportion of surviving N2 control (blue), neuronal *bet-1* (*rgef-1p::bet-1*; green), N2 + *daf-16* RNAi (blue, hollow circles), and neuronal *bet-1* + *daf-16* RNAi (green, hollow circles) animals exposed to PA14 at day 1 of adulthood. Survival was scored every 2 h for 8 h. Data are pooled from three biological replicates, each containing three technical replicates. (****) *P* < 0.0001, (***) *P* < 0.001, (ns) nonsignificant (*P* > 0.05) by two-way ANOVA. (*F*) Proportion of surviving N2 control (blue), neuronal *bet-1* (*rgef-1p::bet-1*; green), N2 + *hsf-1* RNAi (blue hollow circles), and neuronal *bet-1* + *hsf-1* RNAi (green hollow circles) animals exposed to PA14 at day 1 of adulthood. Survival was scored every 2 h for 8 h. Data are pooled from three biological replicates, each containing three technical replicates. (****) *P* < 0.0001, (***) *P* < 0.001, (**) *P* < 0.01, (*) *P* < 0.05, (ns) nonsignificant (*P* > 0.05) by two-way ANOVA.

Some of the most prevalent GO terms enriched in neuronal *bet-1* animals were related to immune response (Supplemental Fig. S3B,C). Indeed, neuronal *bet-1* animals displayed differential gene expression of many immune response genes, unlike whole-animal *bet-1* overexpression (Fig. 5A). In their natural environment, *C. elegans* are constantly exposed to the risk of bacterial infection due to their dependence on bacteria for food, and breakdown of the gut barrier results in bacterial colonization of the gut and pharynx, and bacterial infection is the leading cause of death in *C. elegans* (Zhao et al. 2017; Egge et al. 2019). Therefore, to directly test response to pathogens, we performed pathogen exposure assays to two opportunistic pathogens, *Enterococcus faecalis* and *Pseudomonas aeruginosa* (PA14). Interestingly, we found that neuronal *bet-1* animals displayed a significant increase in resistance to *E. faecalis* (Fig. 5B). Moreover, by performing a fast kill PA14 assay, we found that neuronal *bet-1* animals exhibited a striking increase in resistance to PA14, far better than the mild increase in PA14 resistance found in whole-animal *bet-1* overexpression (Fig. 5C; Mahajan-Miklos et al. 1999; Cezairliyan et al. 2013). In addition, neuronal *bet-1* animals exhibited a significant reduction in intestinal bloating compared to control animals (Fig. 5D).

Because HSF-1 and DAF-16 have both been reported to also activate immune response (Garsin et al. 2003; Singh and Aballay 2006), we next tested whether the enhanced resistance to PA14 observed in neuronal *bet-1* animals also require these transcription factors. RNAi knockdown of *daf-16* or *hsf-1* partially suppressed the increased resistance to PA14 in neuronal *bet-1* animals (Fig. 5E,F). These results suggest that both DAF-16 and HSF-1 contribute, at least in part, to the enhanced immune resilience observed in these animals. However, neuronal *bet-1* animals remained significantly long-lived when maintained on kanamycin-killed bacteria (Supplemental Fig. S7A; Liu et al. 2018), indicating that improved pathogen resistance, while significant, is unlikely to fully account for the life span extension and suggesting that additional mechanisms, such as enhanced proteostasis, also contribute to longevity in these animals.

*C. elegans* also utilize their nervous system to modulate numerous behavioral responses, including learning behavior to avoid pathogenic bacteria (Nuttley et al. 2002; Zhang et al. 2005; Pradel et al. 2007; Chiang et al. 2022). This behavior is mediated by several neurotransmitters, including serotonin, and certain strains that have increased pathogen resistance show a lack of this typical avoidance behavior (Nair et al. 2024). Therefore, to determine whether the heightened pathogen resistance of neuronal *bet-1* animals drives differences in pathogen avoidance behavior, we used a previously validated forced exposure model (Nair et al. 2024). Indeed, neuronal *bet-1* animals displayed a severe defect in pathogen avoidance, essentially never leaving the PA14 lawn (Supplemental Fig. S7B). To determine whether this behavioral deficit reflected a specific apathy toward pathogens or a broader sensory impairment, we next assessed chemotaxis to attractive odorants. We selected a panel of five well-characterized chemoattractants: three sensed by AWC neurons and two by AWA neurons, and tested animals at day 1 of adulthood (Bargmann 2006; Kauffman et al. 2011; Cheng et al. 2021). Strikingly, neuronal *bet-1* animals exhibited a profound defect in chemotaxis behavior across all odorants tested (Supplemental Fig. S7C). Unlike wild-type controls, which reliably migrated toward the odorant sources, neuronal *bet-1* animals frequently failed to orient or initiate directed movement, resulting in a large fraction of the population remaining in neutral zones of the assay plate (Supplemental Fig. S7D). To capture this deficit, we quantified both the traditional chemotaxis index and the percentage of animals that did not select either odorant or control spots. The elevated proportion of nonchoosing animals in the neuronal *bet-1* animals suggests a generalized failure in sensory-driven decision-making rather than a selective deficit in pathogen aversion. Overall, these data suggest neuronal *bet-1* reduces neuronal sensory functions at the expense of improved peripheral health.

Previous studies have shown that reduced sensory neuron function can extend life span. For example, amphid gustatory neurons, including ASI, tonically signal to limit longevity under well-fed conditions, whereas sensory dysfunction or loss of food-derived cues relieves repression of DAF-16/FOXO-dependent longevity pathways (Apfeld and Kenyon 1999; Shaw et al. 2007). In addition, ablation of olfactory neurons can also extend life span (Alcedo and Kenyon 2004). Because lipophilic dye uptake is disrupted when amphid sensory endings are malformed or improperly exposed to the environment, neuronal dye-filling assays serve as a useful first-pass readout of the structural integrity and potentially functional competence of these ciliated sensory organs (Perkins et al. 1986). Consistent with this framework, neuronal *bet-1* animals exhibited a marked reduction in DiI-labeled sensory neurons by day 9. Notably, even at day 1, these animals showed a modest but significant decrease in labeling, with a median of five out of six amphid sensory neuron pairs labeled, compared to six out of six in N2 controls (Supplemental Fig. S7E,F). This staining pattern suggests that neuronal *bet-1* disrupts amphid sensory neuron integrity in an age-dependent manner, likely impairing sensory function, thus suggesting that life span extension could also be explained in part by reduced sensory neuron function.

Consistent with these functional and structural defects, analysis of our neuronal RNA-seq data set, using curated gene sets based on published work, revealed a broader transcriptional signature suggestive of impaired sensory neuron signaling. Neuronal *bet-1* animals displayed a significant reduction in genes encoding neuron-secreted signaling peptides, which was particularly evident among genes associated with ASI-secreted peptides (Supplemental Fig. S8C). This included the gene encoding TGF-β, *daf-7*, and those encoding the DAF-2 agonists, *ins-6* and *daf-28*. We also observed altered expression of several neurotransmitter-related genes, including several genes linked to monoaminergic signaling such as *tdc-1*, *tbh-1*, *cat-1*, and *dat-1* (Supplemental Fig. S8D), consistent with a general remodeling of neuronal communication (Chase and Koelle 2007; Hobert 2013). Finally, because dye-filling defects often reflect disruption of ciliated sensory endings, we also analyzed expression of genes associated with intraflagellar transport (IFT) and found that several genes including *che-11*, *dyf-19*, and *klp-20*, were differentially expressed in neuronal *bet-1* animals (Supplemental Fig. S8E; Bell et al. 2006; Kobayashi et al. 2007; Ou et al. 2007; Li et al. 2010). Together, these transcriptomic data complement the chemotaxis and dye-filling assays and further support the conclusion that neuronal *bet-1* disrupts sensory neuron function.

Given the defects observed in both chemotaxis and dye-filling assays, we next asked whether neuronal *bet-1* exerts broader detrimental effects on neuronal health. Using a strain expressing neuronal polyQ::YFP, we found that neuronal *bet-1* animals exhibited increased polyQ::YFP signal relative to controls, indicating impaired neuronal proteostasis (Supplemental Fig. S8A,B). Together, these findings support a model in which neuronal *bet-1* promotes beneficial peripheral effects while imposing local costs on neuronal health.

### Neuronal *bet-1* animals have mild effects on lipid metabolism

Finally, we tested the impact of neuronal *bet-1* on metabolic remodeling, another highly enriched class of GO-terms (Supplemental Fig. S3B,C), that is directly relevant to longevity. Increased HSF-1 signaling has been shown to induce mitochondrial remodeling to promote oxidative stress resilience (Tharp et al. 2021). Therefore, we first performed cell type-specific mitochondrial imaging in neuronal *bet-1* animals using a matrix-targeted GFP (MLS::GFP) (Kim et al. 2025b). We found that neuronal *bet-1* animals displayed no major differences in mitochondrial morphology in the hypodermis, intestine, or muscle (Supplemental Fig. S9). Since we did identify differential gene expression of genes related to mitochondria in neuronal *bet-1* animals (Supplemental Fig. S10A), to ensure that the lack of changes in mitochondrial morphology was not due to the resolution limits of standard fluorescence microscopy, we next performed electron microscopy. Higher-resolution imaging to capture ultrastructure of mitochondria consistently showed no changes in neuronal *bet-1* animals (Supplemental Fig. S10B,C). Similarly, we did not see any significant changes in oxygen consumption rate using a Seahorse assay (Supplemental Fig. S10D; Ng and Gruber 2019).

We also observed significant changes to genes involved in lipid homeostasis (Supplemental Fig. S11A). Therefore, we assessed lipid content by visualizing lipid droplets, the main organelles responsible for storing intestinal lipids in *C. elegans* (Lemieux and Ashrafi 2015). To visualize lipid droplets, we utilized the DHS-3::GFP reporter, a fluorescently tagged short-chain dehydrogenase, which is abundant on the surface of intestinal lipid droplets (Na et al. 2015). We found that neuronal *bet-1* animals displayed no change in lipid droplet levels compared to wild-type control at any age (Supplemental Fig. S11B,C). However, *bet-1* knockdown reduced lipid droplet levels at days 4 and 7 of adulthood, suggesting that *bet-1* has some functions in lipid handling.

Because we did not observe differences in DHS-3::GFP-labeled lipid droplet abundance, despite significant changes in lipid metabolism genes in neuronal *bet-1* animals, we next assessed neutral lipid content using complementary staining approaches. BODIPY (Klapper et al. 2011) is a fluorescent dye that labels neutral lipids and is commonly used to visualize lipid droplets, whereas Oil Red O (O'Rourke et al. 2009) is a colorimetric dye that detects bulk neutral lipid stores, including triglycerides and cholesteryl esters. Using these assays, we observed partially divergent results. BODIPY staining revealed elevated neutral lipid signal in neuronal *bet-1* animals on day 1 of adulthood, an effect that was suppressed by *hsf-1*, but not *daf-16*, RNAi. However, by day 5, BODIPY signal was no longer significantly different between neuronal *bet-1* and control animals (Supplemental Fig. S12A,B). In contrast, Oil Red O staining showed very small differences. At day 1, no quantifiable difference was observed, while at day 5, a very mild but statistically significant reduction was observed in neuronal *bet-1* animals (Supplemental Fig. S12C,D). At first glance, these results appear inconsistent. However, this divergence likely reflects the distinct lipid pools and physical properties captured by each method. DHS-3::GFP labels a subset of lipid droplets, BODIPY preferentially detects more accessible or dynamic neutral lipid pools, and Oil Red O provides a bulk end point measure of stored lipids. As such, these approaches differ in sensitivity, spatial resolution, and biochemical specificity, and are not expected to report uniformly on complex and temporally dynamic lipid remodeling processes. Taken together, these findings suggest that neuronal *bet-1* does not induce a simple increase or decrease in total lipid storage but rather drives age-dependent remodeling of distinct neutral lipid pools that are differentially captured by conventional staining methods. Importantly, the variability across assays highlights a key limitation of dye-based approaches: they provide only coarse and method-dependent readouts of lipid abundance, without resolving lipid species composition.

**Figure 6.**
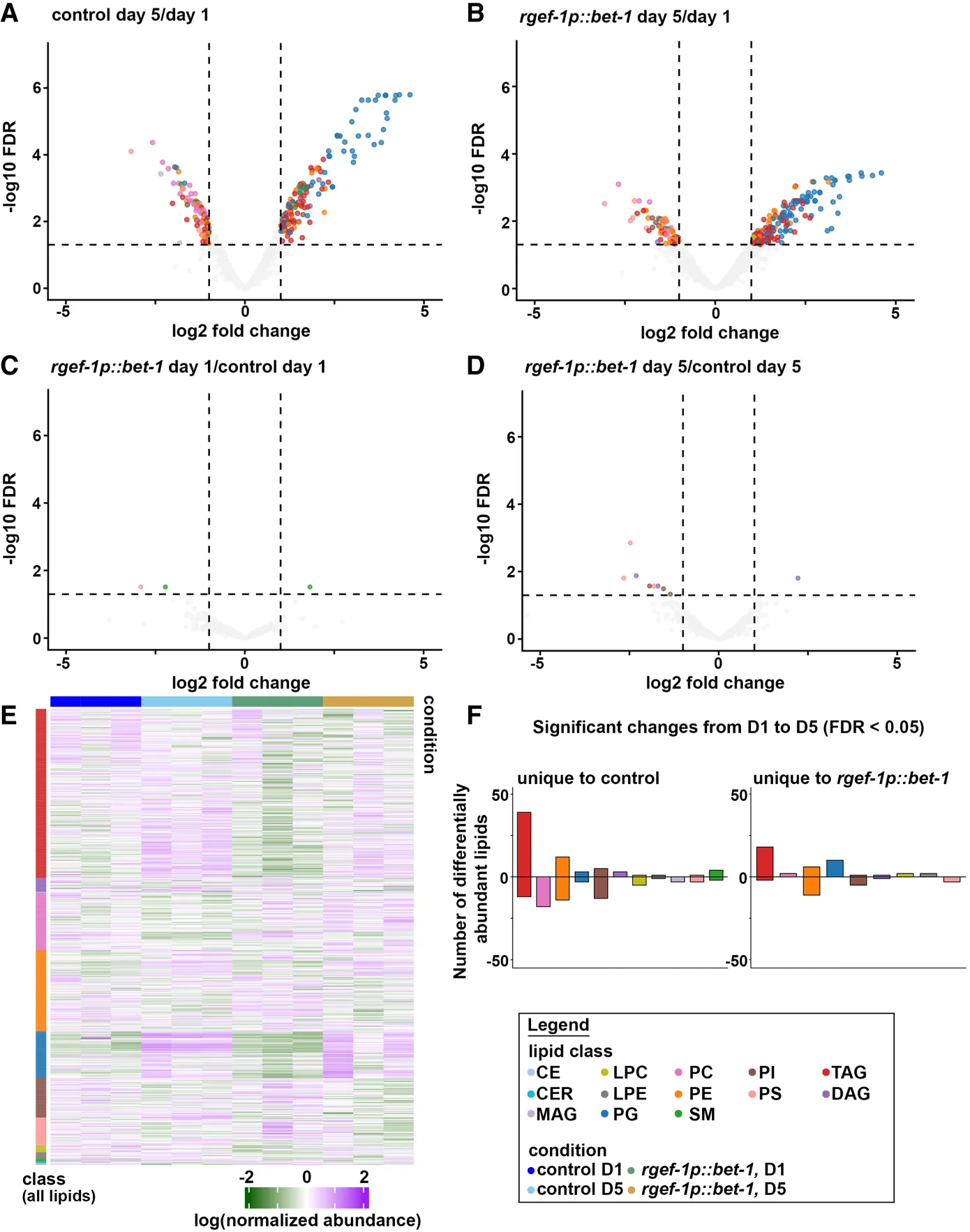
Neuronal *bet-1* has only minor changes to lipid levels in late life. Lipidomics was performed in day 1 and day 5 animals in *glp-4(bn2)* animals grown at 22°C to eliminate contamination from progeny and germline. (*A*) Volcano plot showing all differentially abundant lipids for control day 5 animals compared to control day 1 animals. (*B*) Volcano plot showing all differentially abundant lipids for neuronal *bet-1* (*rgef-1p::bet-1*) day 5 animals compared to neuronal *bet-1* day 1 animals. (*C*) Volcano plot showing all differentially abundant lipids for neuronal *bet-1* (*rgef-1p::bet-1*) day 1 animals compared to control day 1 animals. (*D*) Volcano plot showing all differentially abundant lipids for neuronal *bet-1* (*rgef-1p::bet-1*) day 5 animals compared to control day 5 animals. (*E*) Spearman correlation plot of all lipids measured. (Green) Lower abundance, (purple) higher abundance. Plots specific for neutral lipids and membrane lipids are available in Supplemental Figure S13, D and E. All raw values are in Supplemental Table S5. (*F*) Unique differentially abundant lipids were calculated comparing day 5 versus day 1 for control (*left*) and neuronal *bet-1* (*rgef-1p::bet-1*) (*right*). All positive numbers are numbers of lipids in each category that are increased in abundance with age and all negative numbers are numbers of lipids in each category that reduced in abundance with age in each condition. The legend at the *bottom right* applies to *A*–*F*. Significance thresholds of log_2_FC of >1 or <−1 and FDR of <0.05 were set for *A*–*D*.

To overcome these limitations and obtain a comprehensive, quantitative view of lipid changes, we therefore performed a targeted, quantitative lipidomic analysis to thoroughly evaluate whether changes in lipids occur in neuronal *bet-1* animals, reflective of transcriptional changes. Once again, we utilized *glp-4(bn2)* animals to avoid contamination from germline. We first confirmed that this assay was able to find significant differences in lipids during natural aging (Fig. 6A). Day 5 animals exhibited an increase in triglycerides (TAGs) and phosphatidylglycerols (PGs) compared to day 1 animals. This is consistent with prior work showing that lipid droplets, which primarily store TAGs, accumulate progressively with age in *C. elegans*, providing orthogonal evidence that neutral lipid stores expand during aging (Palikaras et al. 2023). This observation is further supported by findings in mammals showing similar increases in TAGs in multiple tissues, including well-documented increases in hepatic steatosis (Ogrodnik et al. 2017) and intramyocellular lipid deposition (Cree et al. 2004), reflecting a conserved shift toward ectopic neutral lipid storage and impaired lipid homeostasis with age. In addition to changes in TAGs, cardiolipin, which is synthesized from phosphatidylglycerol (PG), declines during aging, contributing to defects in mitochondrial function (Hou and Taubert 2012). Thus, it is plausible that PG accumulation is due to the failure in conversion to cardiolipin with age.

We found similar changes with age in PGs and TAGs in neuronal *bet-1* animals (Fig. 6B), suggesting that neuronal *bet-1* animals do not delay the observed age-associated changes in lipid profile. Indeed, direct comparison of neuronal *bet-1* animals to control did not show major differences at any age with the exception of a few specific lipids (Fig. 6C,D). A more zoomed-out, population-level analysis showed that neuronal *bet-1* animals differ more from control animals at day 5 compared to day 1 (Fig. 6E; Supplemental Fig. S13A), suggesting that there were some distinctions in age-related changes in lipid profiles, although these changes were not dramatic. Further analysis revealed that although 164 differentially abundant lipids with age are shared in neuronal *bet-1* animals and control animals, a total of 207 differentially abundant lipids are unique to either condition (Supplemental Fig. S13B,C). Plotting differentially abundant lipids with age unique to each condition showed that neuronal *bet-1* animals generally exhibit slightly dampened age-related responses, including a reduced number of TAGs that increase in abundance with age, lower numbers of phosphatidylcholines (PCs) and phosphatidylinositols (PIs) that decrease in abundance with age, and fewer differentially abundant sphingomyelins (SMs) and monoacylglycerols (MAGs) with age compared to control animals (Fig. 6F). Again using a zoomed out, population level analysis, looking specifically at neutral lipids (Supplemental Fig. S13D) or membrane lipids (Supplemental Fig. S13E), differences can be seen between control and neuronal *bet-1* animals.

Consistent with our findings, previous work has shown that an increase in TAG abundance occurs with aging in *C. elegans* (Staab et al. 2023), whereas PCs and SMs decline with age (Wan et al. 2019). Importantly, SMs are directly correlated with longevity, with a predicted increase in sphingomyelinase activity with age and mutants of the sphingomyelinase enzyme, *asm-3* exhibiting increased life span (Staab et al. 2023). Furthermore, reduction in TAGs by increased lysosomal lipases or desaturases can also extend longevity (Imanikia et al. 2019a,b). Additional work has hypothesized that a reduction in PC synthesis with aging can be a driver of natural mitochondrial aging (Poliezhaieva et al. 2026). Altogether, our data suggest that although neuronal *bet-1* does not result in major changes to the overall lipid profile, the few changes that do occur are predominantly associated with aging and longevity, suggesting that the lipid changes in neuronal *bet-1* animals, while mild, could potentially be drivers of prolongevity.

### Neuronal *bet-1* animals activate daf-16 and hsf-1 to promote longevity

**Figure 7.**
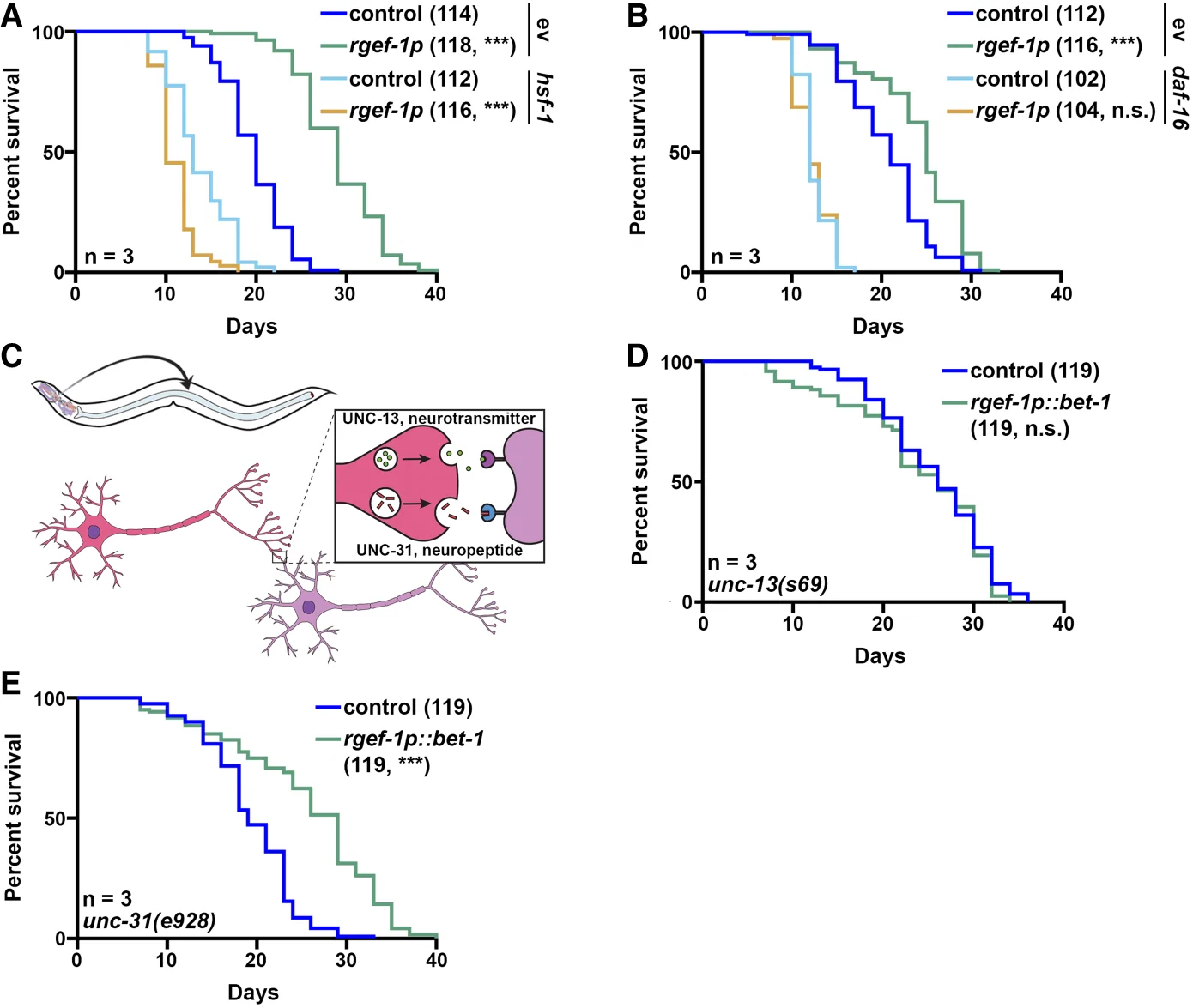
Neuronal *bet-1* extends life span through neurotransmitter signaling. (*A*) Life spans of N2 control and neuronal *bet-1* (*rgef-1p*) animals grown on EV or *hsf-1* RNAi from L1. (***) *P* < 0.001 using log rank testing. See Supplemental Table S1 for complete life span statistics. (*B*) Life spans of N2 control and neuronal *bet-1* (*rgef-1p*) animals grown on EV or *daf-16* RNAi from L1. (***) *P* < 0.001, (n.s.) not significant (*P* > 0.05) using log rank testing. See Supplemental Table S1 for complete life span statistics. (*C*) Schematic of neuronal signaling mediated by neurotransmitter release through UNC-13 and neuropeptide release through UNC-31. (*D*) Life span comparing *unc-13(s69)* neurotransmitter release mutants with (green) and without (control; blue) the *rgef-1p::bet-1B* transgene grown on EV from L1. (n.s.) not significant (*P* > 0.05) by log rank testing. See Supplemental Table S1 for complete life span statistics. (*E*) Life span comparing *unc-31(e928)* neuropeptide release mutants with (green) and without (control, blue) the *rgef-1p::bet-1B* transgene grown on EV from L1. (***) *P* < 0.001 using log rank testing. See Supplemental Table S1 for complete life span statistics.

Overall, our data show that neuronal *bet-1* extensively remodeled the transcriptome in a DAF-16 and HSF-1 dependent manner, which dramatically impacted animal health and physiology. Therefore, we next sought to determine whether *hsf-1* and *daf-16* were required for the life span extension of neuronal *bet-1* animals. Indeed, we found that RNAi knockdown of *hsf-1* (Fig. 7A) or *daf-16* (Fig. 7B) fully suppressed the life span extension of neuronal *bet-1* animals. In fact, neuronal *bet-1* overexpression further reduced the short life span of *hsf-1* RNAi animals, suggesting that when peripheral prolongevity phenotypes are not activated, the trade-off in neuronal health may be toxic.

Since the activation of HSF-1 can promote longevity through several downstream effector pathways, including the UPS, autophagy (Kumsta et al. 2017), and improved actin integrity (Baird et al. 2014; Higuchi-Sanabria et al. 2018), we next sought to determine which of these pathways is required for neuronal *bet-1*-mediated life span extension. To assess the role of the UPS, we performed RNAi knockdown of *rpn-6.1*, which encodes a critical proteasome subunit. Loss of *rpn-6.1* partially suppressed the life span extension of neuronal *bet-1* animals (Supplemental Fig. S14A), suggesting a contributory but nonessential role for proteasome function. Next, we examined the role of autophagy. Given that *bec-1* is a core early acting autophagy gene required for autophagosome formation, we tested whether RNAi against *bec-1* impacts life span extension. Interestingly, we found that autophagy was largely not required for the life span extension (Supplemental Fig. S14B), which is consistent with our data suggesting that autophagy was not largely required for reduction in the accumulation of polyQ foci in neuronal *bet-1* animals. Finally, we tested whether maintenance of actin integrity is required for this phenotype, as both *hsf-1* and *bet-1* are required for proper actin maintenance with age (Baird et al. 2014; Higuchi-Sanabria et al. 2018; Garcia et al. 2023). Because strong *act-1* knockdown is lethal, we used a sublethal 10% dilution of *act-1* RNAi (Garcia et al. 2023). Under these conditions, *act-1* knockdown fully suppressed the life span extension of neuronal *bet-1* animals, demonstrating that actin function is required for this effect (Supplemental Fig. S14C). Together, these results identify maintenance of actin integrity as a central requirement for neuronal *bet-1*-mediated longevity, with the UPS and autophagy contributing more modestly.

We next hypothesized that the life span extension of neuronal *bet-1* animals occurred through a neuron-to-body cell-nonautonomous signal, as previous reports have shown that neuronal overexpression of *hsf-1* increased life span by similarly activating *hsf-1* and *daf-16* in peripheral tissues (Douglas et al. 2015). In *C. elegans*, neurons signal either through neurotransmitters released via small clear vesicles mediated by UNC-13 or through neuropeptides released via dense core vesicles mediated by UNC-31 (Fig. 7C). Both UNC-13- and UNC-31-mediated signals have been previously described in stress signaling from neural cells (Taylor and Dillin 2013; Frakes et al. 2020; Bar-Ziv et al. 2023). Therefore, to test a role for neuronal signaling in the life span extension of neuronal *bet-1* animals, we examined life spans of neurotransmitter (*unc-13(s69)*) and neuropeptide (*unc-31(e928)*) release mutants. We found that blocking neurotransmitter release (Fig. 7D), but not neuropeptide release (Fig. 7E), resulted in suppression of the life span extension found in neuronal *bet-1* animals, suggesting that neuronal *bet-1* promotes longevity through a neuron-to-body signaling modality mediated by neurotransmitter signaling.

**Figure 8.**
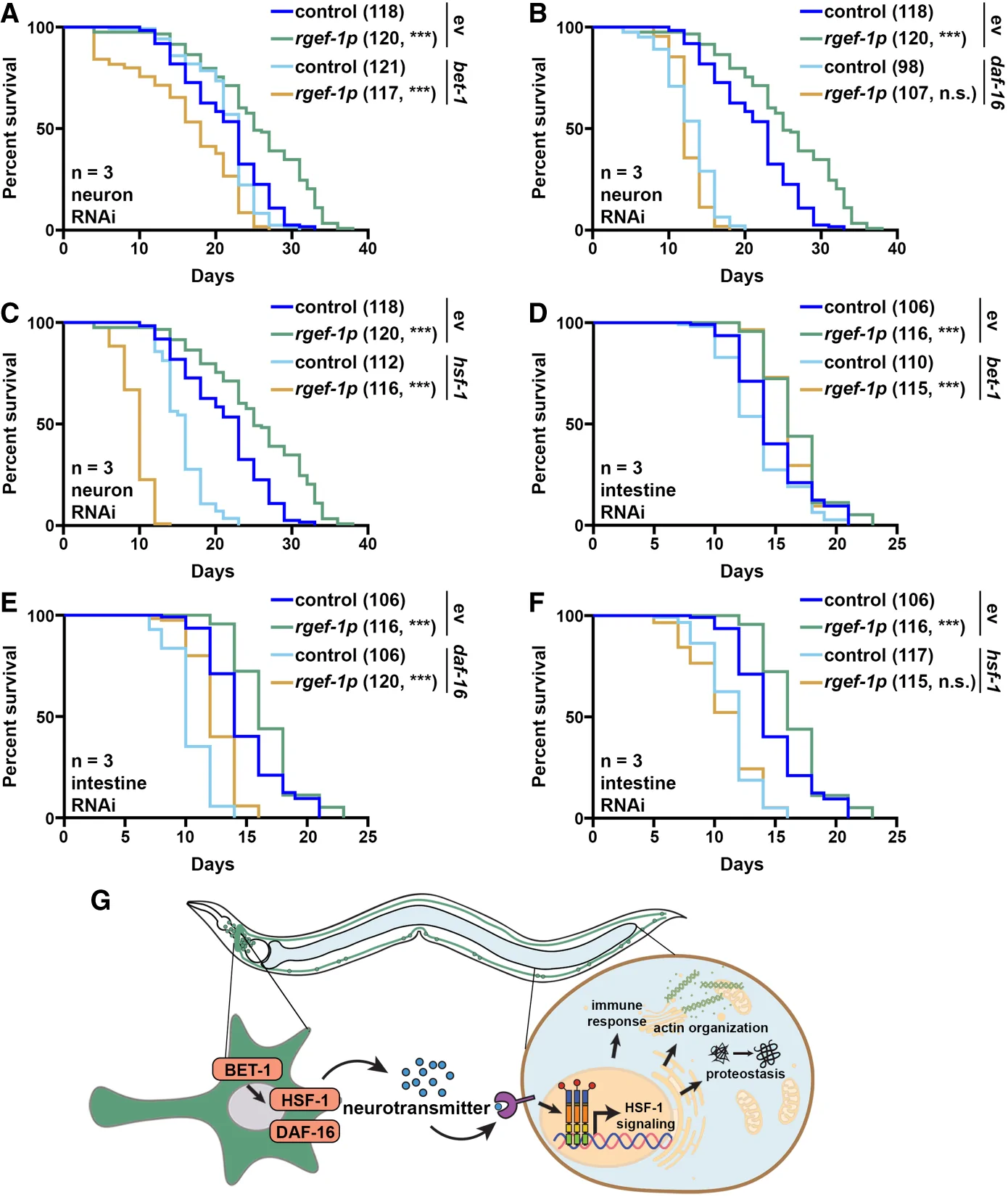
Neuronal *bet-1* animals require *hsf-1* in neurons and intestine and *daf-16* and *bet-1* in neurons for life span extension. (*A*) Life spans of MAH667 (neuron-specific RNAi) control and MAH677 with neuronal *bet-1* overexpression (*rgef-1p*) grown on EV or *bet-1* RNAi from L1. (*B*) Life spans of MAH667 (neuron-specific RNAi) control and MAH677 with neuronal *bet-1* overexpression (*rgef-1p*) grown on EV or *daf-16* RNAi from L1. (*C*) Life spans of MAH667 (neuron-specific RNAi) control and MAH677 with neuronal *bet-1* overexpression (*rgef-1p*) animals grown on EV or *hsf-1* RNAi from L1. For *A*–*C*, all life spans were performed at the same time and can be directly compared to each other. (*D*) Life spans of VP303 (intestine-specific RNAi) control and VP303 with neuronal *bet-1* overexpression (*rgef-1p*) grown on EV or *bet-1* RNAi from L1. (*E*) Life spans of VP303 (intestine-specific RNAi) control and VP303 with neuronal *bet-1* overexpression (*rgef-1p*) grown on EV or *daf-16* RNAi from L1. (*F*) Life spans of VP303 (intestine-specific RNAi) control and VP303 with neuronal *bet-1* overexpression (*rgef-1p*) animals grown on EV or *hsf-1* RNAi from L1. For *D*–*F*, all life spans were performed at the same time and can be directly compared to each other. (***) *P* < 0.001, (n.s.) not significant (*P* > 0.05) using log rank testing compared to their respective controls. Life span conditions were separated for ease of visibility. See Supplemental Table S1 for complete life span statistics. (*G*) Model for neuronal *bet-1* driving proteostasis via HSF-1 and immune response to promote longevity.

To gain a better understanding of the tissue-specific requirements of BET-1, HSF-1, and DAF-16 in both the neuronal signal and peripheral response, we performed neuron- and periphery (i.e., intestine)-specific RNAi using cell type-specific RNAi strains (Espelt et al. 2005; Yang et al. 2024). Interestingly, we found that the life span extension of neuronal *bet-1* required *bet-1*, *hsf-1*, and *daf-16* in neurons (Fig. 8A–C), suggesting that neuronal *bet-1* activates both HSF-1 and DAF-16 in the neurons, which then elicit a signal to the periphery. Once again, neuronal *hsf-1* knockdown resulted in further reduction of life span in neuronal *bet-1* animals, further supporting the model that without HSF-1 activation, neuronal *bet-1* can have toxic effects. These data raised an intriguing question of whether BET-1 in neurons can promote HSF-1 activity directly in neurons, which then elicits a neuronal signal to drive peripheral activation of BET-1, HSF-1, and/or DAF-16. Indeed, previous work has shown that neuronal *hsf-1* can drive heterotypic activation of both HSF-1 and DAF-16 in the periphery (Douglas et al. 2015). Therefore, we next tested whether neuronal *hsf-1* also required *bet-1* in neurons. Interestingly, we found that neuronal *hsf-1* does not require *bet-1* or *daf-16* in neurons (Supplemental Fig. S14D). In addition, neuronal *hsf-1* likely does not require *bet-1* in any tissue, as whole-animal *bet-1* RNAi did not suppress the life span extension of these animals (Supplemental Fig. S14E). These data are consistent with a model whereby neuronal *bet-1* is upstream of neuronal *hsf-1*, which can drive nonautonomous communication to the periphery.

To further test this model, we performed intestine-specific RNAi of *bet-1*, *daf-16*, and *hsf-1* in neuronal *bet-1* animals. Interestingly, only *hsf-1* was required in the intestine (Fig. 8D–F), suggesting that the requirement of *bet-1* and *daf-16* was limited to neurons, whereas *hsf-1* was required in both the neurons and the intestine. Altogether, our data suggest a potential model whereby neuronal *bet-1* activates neuronal DAF-16 and HSF-1, which drives intestinal activation of HSF-1 to promote longevity (Fig. 8G). Importantly, this model is distinct from the previously characterized neuronal *hsf-1* model, which activates both HSF-1 and DAF-16 in the periphery (Douglas et al. 2015).

## Discussion

Our work reveals that BET-1, a chromatin reader traditionally linked to establishment and maintenance of cell fate, also plays a critical role in regulating stress responses and longevity. By overexpressing *bet-1* specifically in neurons, we uncover a powerful neuron-to-periphery signaling axis that promotes organismal longevity through peripheral activation of the canonical stress regulator, HSF-1. This represents a paradigm shift in our understanding of how epigenetic readers like BET-1 can function not only in local chromatin regulation, but also as initiators of systemic stress adaptation.

Importantly, the effects of neuronal *bet-1* overexpression differ markedly from previously reported whole-animal *bet-1* overexpression (Garcia et al. 2023). This divergence may stem from promoter strength and tissue-specific expression. While whole-animal overexpression used the endogenous *bet-1* promoter (yielding modest expression under physiological control), our neuron-specific construct employed the *rgef-1* promoter, which produces much higher expression levels in neurons. This amplification, coupled with the unique signaling capacity of neurons, likely enables novel phenotypes absent in whole-animal *bet-1* models, including more robust life span extension and distinct transcriptional signatures. This is not unique to *bet-1*, as neuronal and whole-animal *xbp-1s* overexpression also displays significant differences in capacity to alter stress resilience and longevity (Taylor and Dillin 2013).

Neuronal *bet-1* signal transmission was dependent on neurotransmitter signaling, paralleling previously identified neuron-to-periphery signals such as neuronal *hsf-1*, and *xbp-1s* (Taylor and Dillin 2013; Douglas et al. 2015). In each of these nonautonomous signaling paradigms, peripheral effects act on the intestine (Ailion et al. 1999; Douglas et al. 2015; Zhang et al. 2018; Imanikia et al. 2019a; Özbey et al. 2020). However, both neuronal *hsf-1* and *xbp-1s* showed some distinct phenotypic effects on other peripheral tissues as well, including the muscle and hypodermis (Higuchi-Sanabria et al. 2018; Imanikia et al. 2019a), whereas the nonautonomous effects of neuronal *bet-1* were mostly limited to the intestine. It is possible that these effects are due to the specific neurotransmitters that are utilized in each signaling paradigm, whereby neuronal *bet-1* utilizes specific signaling molecules that only impact the intestine. Further research to investigate which specific neurotransmitters are involved in the distinct peripheral responses would be highly intriguing, especially considering the divergent signaling that exists for neuronal *xbp-1s*. Neuronal *xbp-1s* utilizes distinct neuronal signaling including serotonergic and glutamatergic signaling for peripheral proteostasis, dopaminergic signaling for lipid homeostasis, and octopaminergic signaling for immune response (Higuchi-Sanabria et al. 2020; Coakley et al. 2025). Moreover, we found in our RNA-seq data set that tyramine and octopamine synthesis genes were upregulated in neuronal *bet-1* and the dopamine receptor, *dop-2*, was downregulated. The TGF-β gene, *daf-7*, was also greatly downregulated along with other products of ASI neurons, an effect observed in the nonautonomous communication of longevity under dietary restriction or absence of food odor (Fletcher and Kim 2017). These data suggest a tantalizing hypothesis whereby neuronal *bet-1* also utilizes distinct neurotransmitters to elicit each unique peripheral response.

Unlike neuronal *xbp-1s*, which also activates *xbp-1s* in the periphery to drive beneficial effects, neuronal *bet-1* activated a unique response whereby *daf-16* and *hsf-1* are also required in neurons, but only *hsf-1* is required in the intestine. Moreover, *bet-1* is not required for the life span extension of neuronal *hsf-1* animals, suggesting that HSF-1 acts downstream from *bet-1*, potentially acting as an integrator or amplifier of stress-responsive chromatin states. Indeed, BRD4 has been shown to occupy enhancers of FOXO targets, and BRD4 colocalizes with HSF1 at nuclear stress bodies during heat shock (Col et al. 2017; Nagarajan et al. 2017). Thus, neuronal *bet-1* can potentially activate neuronal HSF-1 and DAF-16, driving nonautonomous activation of HSF-1 prolongevity pathways in the periphery (Douglas et al. 2015; Uno et al. 2021). Still to be understood is how neuronal *bet-1* activates DAF-16 and HSF-1 in neurons and how this specifically activates an HSF-1 signature independent of peripheral DAF-16.

Neuronal *bet-1* promotes multiple prolongevity phenotypes, but also incurs modest healthspan trade-offs, including a mild reduction in motility. At the same time, these animals display striking resilience to infection, with enhanced resistance to bacterial pathogens and reduced PA14-induced intestinal bloating. In *C. elegans*, DAF-16 protects against bacterial infection through an HSF-1-dependent pathway, with HSF-1 acting both cell-autonomously and nonautonomously in DAF-16 signaling paradigms (Singh and Aballay 2006; Lee et al. 2021). In addition, intestinal HSF-1 can promote barrier integrity by maintaining interactions between ACT-5 and DLG-1 (Egge et al. 2019). However, in neuronal *bet-1* animals, DAF-16 is not required in the periphery, and despite elevated HSF-1 activity, intestinal actin is not detectably preserved. This suggests that the protection against intestinal bloating arises primarily from enhanced immune function rather than improved structural integrity of the intestine. Consistent with this interpretation, prior work has demonstrated that HSF-1 outputs are functionally separable, with distinct transcriptional programs regulating cytoskeletal integrity and proteostasis (Baird et al. 2014). Thus, neuronal *bet-1* may preferentially engage immune and proteostatic branches of HSF-1 signaling without equivalently reinforcing intestinal actin architecture.

Another notable finding was that disruption of the actin cytoskeleton was sufficient to fully suppress life span extension in neuronal *bet-1* animals, despite only modest effects on stabilization of actin during aging in this model. It is possible that this requirement for actin may be due to the multifunctional roles of actin. For example, actin dynamics are required for autophagosome formation, linking cytoskeletal integrity to autophagic function and proteome maintenance (Lange et al. 2025). In addition, directed destabilization of actin in *C. elegans* is sufficient to induce pleiotropic aging pathology, including increased protein aggregates, dysregulation of lipids, dysregulated autophagy, and disrupted gut barrier function (Averbukh et al. 2025), all relevant phenotypes we observed in neuronal *bet-1* animals.

An important trade-off associated with neuronal *bet-1* is reduced neuronal function. It is not uncommon for life span extension paradigms to exhibit some trade-offs; for example, long-lived mitochondrial stress models and calorie restriction models have reduced animal size (Durieux et al. 2011; Bar et al. 2016), while neuronal ER stress signaling paradigms exhibit reduced brood size (Özbey et al. 2020). In this context, neuronal *bet-1* animals exhibit defects in multiple neurobehavioral outputs, including pathogen avoidance, chemotaxis, and locomotion, revealing a paradoxical dissociation between neuronal performance and peripheral health. One explanation for this trade-off is that neuronal *bet-1* impairs the function of ciliated sensory neurons that detect food availability and relay those cues to nonautonomous control of aging. This model is supported by the dye-filling defects we observe, as well as by prior work showing that reduced chemosensory function can itself promote longevity, particularly through gustatory neurons and ASI-dependent pathways (Apfeld and Kenyon 1999). More broadly, food-derived sensory cues suppress life span by activating insulin-like signaling and inhibiting DAF-16/FOXO; even a single attractive odor can attenuate longevity under dietary restriction conditions (Park et al. 2021). Thus, rather than reflecting a simple global improvement in neuronal health, neuronal *bet-1* may partially uncouple sensory perception of food abundance from the downstream proaging signals that normally restrain longevity. A similar principle has also emerged in mammalian studies, in which sensory perception shapes systemic aging-related physiology. In mice, conditional ablation of olfactory sensory neurons remodels adipose metabolism and protects against diet-induced obesity, while transient early-life exposure to same-sex odors extends life span in female mice (Riera et al. 2017). Although these findings do not imply mechanistic conservation, they support a broader principle that sensory perception of external cues can modulate organismal aging across phyla.

This framework also suggests specific candidate sensory nodes involved in this process. The chemotaxis assays specifically implicate dysfunction of AWA and AWC neurons, the principal amphid olfactory neurons for attractive volatile food cues and thereby identify food-odor circuits involved in sensory control of life span as plausible upstream regulators of the neuronal *bet-1* phenotype (Bargmann et al. 1993; Chalasani et al. 2010). In parallel, the dye-filling phenotype is consistent with dysfunction among a distinct subset of DiI-positive amphid neurons, supporting the interpretation that neuronal *bet-1* broadly perturbs sensory function across multiple amphid modalities. Particularly intriguing in this context is the marked reduction of *daf-7* and other ASI-enriched transcripts in our RNA-seq data. *daf-7*, produced primarily by ASI in response to food-associated sensory state, is reduced by starvation and functions as a neuroendocrine regulator of physiology and life span (Murakami et al. 2001; Entchev et al. 2015). Importantly, DAF-7/DAF-1 signaling has also been shown to restrain tyraminergic and octopaminergic output from the RIM/RIC circuit (Greer et al. 2008). In that context, the upregulation of *tdc-1* and *tbh-1* identified by RNA-seq in neuronal *bet-1* animals raises the possibility that sensory dysfunction, potentially centered on ASI and/or food-odor circuits, relieves DAF-7-dependent restraint on downstream tyramine/octopamine signaling, thereby contributing to life span extension and peripheral remodeling. A recent ASI-RIM study is notable in this regard, as it showed that ASI-centered TGF-β signaling can couple neuronal stress to intestinal adaptation, pathogen resistance, altered fat metabolism, and longevity (Wang et al. 2024). Because UNC-13 is dedicated primarily to synaptic vesicle exocytosis, whereas neuroendocrine secretion is more closely associated with UNC-31-dependent dense-core vesicles, this favors a model in which altered ASI/DAF-7 signaling acts upstream of the final UNC-13-dependent effector rather than constituting that effector itself (Richmond et al. 1999; Speese et al. 2007).

At the molecular level, our findings align with emerging evidence that bromodomain-containing proteins like BET-1/BRD4 are not merely passive histone readers, but dynamic regulators of stress response. BRD4 is dynamically recruited with HSF1 to maintain splicing under heat stress and the chromatin acetylation landscape modulates stress responses and longevity across a diverse range of model systems (Hussong et al. 2017). We propose that BET-1 orchestrates chromatin remodeling events that can prime cells—both neurons and peripheral cells—to stress signaling. This may involve preserving open chromatin at stress gene loci, directly recruiting stress regulators, such as DAF-16 and HSF-1. In doing so, BET-1 enables a coordinated transcriptional response that maintains proteostasis and immune competence during aging. However, we found that lipid metabolic health of neuronal *bet-1* animals is largely unchanged, which was surprising, as we observed significant changes in metabolism-related gene expression, including mitochondrial function and lipid metabolism. However, it should be noted that while changes to lipid profiles were modest, the significant changes observed were largely associated with prolongevity pathways. An increase in TAGs (Staab et al. 2023) and a reduction in PCs (Poliezhaieva et al. 2026) and SMs (Staab et al. 2023) are all correlated with aging, and thus suppressing these changes could potentially be drivers of prolongevity in neuronal *bet-1* animals. Further research is critical to investigate whether these modest changes in these lipid species would be sufficient to promote longevity. For example, our studies only provide snapshots of lipid levels at discrete time points—it is possible that neuronal *bet-1* animals have differences in lipid flux due to changes in metabolic enzymes that cannot be observed with single snapshot studies. Additionally, it is important to acknowledge that no single analytical method can capture the full diversity of lipid classes. This is especially important considering variable effects seen when utilizing lipid droplet markers versus neutral lipid dyes, including BODIPY and Oil Red O. These inconsistent data highlight that more detailed analyses of lipids—including flux and more robust quantification of individual lipid species—are required to provide deeper insights into the potential metabolic mechanisms underlying the effect of *bet-1* on aging.

Altogether, our work extends the concept of neuron-to-body signaling beyond transcription factors to include the epigenetic reader, BET-1, suggesting new possibilities for modulation of chromatin remodeling factors in stress resilience and aging. Epigenetics are known to drive changes to healthy aging and life span; and here, we show that manipulation of epigenetic readers can directly drive organism-wide stress resilience and longevity (Benayoun et al. 2015). Future studies should dissect the precise chromatin landscapes and neuronal circuits modulated by BET-1 and explore whether BET-1's chromatin regulatory activity is recruited endogenously during natural stress or aging states.

## Materials and methods

### *Caenorhabditis elegans* strains and maintenance

All strains utilized in this research are derived from the N2 wild-type strain obtained from the *Caenorhabditis* Genetics Center (CGC), as specified in Supplemental Table S7. Worms were routinely maintained at 15°C on standard nematode growth media (NGM) plates seeded with OP50 *Escherichia coli* B strain. NGM plates contained 1 mM CaCl_2_, 12.93 µM (5 µg/mL) cholesterol, 25 mM KPO_4_ (pH 6.0), 1 mM MgSO_4_, 2.5 mM (0.25% [w/v]) peptone, and 51.3 mM NaCl. For experiments, animals were grown at 20°C on RNAi plates (NGM plates supplemented with 1 mM IPTG and 100 µg/mL carbenicillin) seeded with HT115 *E. coli* K strain, which carries either the pL4440 empty vector (EV) or a pL4440 construct expressing double-stranded RNA against the gene of interest. Animals were age-matched via a conventional bleaching method (Bar-Ziv et al. 2020), summarized as follows: Worms were collected in M9 buffer (22 mM KH_2_PO_4_, 42.3 mM Na_2_HPO_4_, 85.6 mM NaCl, 1 mM MgSO_4_) and then treated with 1.8% sodium hypochlorite and 0.375 M KOH in M9 until only eggs remained. These eggs were washed four times with M9 and subsequently left in M9 overnight (up to 16 h) at 20°C on a rotator to synchronize at the L1 stage and plated onto RNAi plates.

For *bet-1* overexpression, the coding region of *bet-1* isoform B (Garcia et al. 2023) was amplified from cDNA generated by reverse transcription of RNA isolated from N2 worms, while the *rgef-1* promoter, other tissue-specific promoters, and the *unc-54* 3′ UTR were amplified from N2 genomic DNA. Plasmids were microinjected into N2 worms according to a standard protocol using 10 ng/µL overexpression construct, 2.5 ng/µL pEK2 (*myo-2p::tdTomato*) as a coinjection marker, and 100 ng/µL pD64 as filler DNA. Worms displaying red fluorescent signal in the pharynx were chosen to identify stable extrachromosomal arrays. Integration into the genome was achieved by γ-irradiation of L4 worms at 4000–4400 rems, followed by isolation of lines maintaining 100% coinjection marker expression in the F3 generation. These integrated lines were backcrossed at least eight times into N2 to remove secondary mutations. We have generated two distinct integrated lines of neuronal *bet-1* overexpression (RHS15 and RHS28). These two lines were not distinguishable in terms of life span and other physiological phenotypes and thus are used interchangeably in the text.

#### bet-1B

The sequence for *bet-1B* was as follows: ATGTCTGAGGGCAGCGGAGACCAATCACAACAACGACCATGGGCAAGTCCGCGACAGCAACCAATCAAAGGAATCGTACAGCCACGAGTACTTCCACCATTCGGAAAGCCAACACGACACACAAACAAACTGGACTACATTATGACAACAGTACTCAAAGAGGCTGGAAAACATAAACATGTCTGGCCGTTTCAGAAGCCCGTCGATGCGGTTGCTTTATGTATTCCTCTATATCACGAGAGAGTCGCCCGACCAATGGACTTGAAAACAATCGAGAATAGACTGAAAAGTACTTATTACACATGTGCTCAAGAATGCATTGATGATATCGAAACAGTTTTCCAAAACTGCTACACATTCAATGGGAAAGAGGACGACGTGACAATTATGGCCCAAAATGTGCACGAAGTGATAAAAAAGTCACTGGAACAAGCACCTCGCGAAGAGCATGATATGGATGTTTATTGGGGAAAAAATAAGAAAAAACCGGCAAAAAGTGACGGTGGATCGAAATCTTCGTCGAGCAAGAAGAATGATGCTCGTGGACCATCTGAAGCACCGTCAGAGGCTGGAAGTGAAGTTTCGTCTGTAACAACAGCATCAGCAGCAGCCCCGACGGTTTCTGAGTCTGCGAGTGTTGCCGCGAAGCCAGAACGAAAAGTGGCCGGAAAGAAGACGGGAAAACGAAAAGCCGAATCAGAAGATGACGAGAAGCCGGAACCTTTGAGAGCAAAACGAGAGGTGGCTGTTGTCAAAAAAGAAGTTCATCAGCCATTGCTCCCAAGTATGAAGCCCTGTCTGAAGCTGCTCAATGATTTTTCTACAAAAAAATATCAGGAATTTGCTTGGCCATTCAACGAACCAGTAGACGCTGAACAACTGGGACTCCATGATTATCATAAAATTATCAAAGAACCAATGGATCTGAAATCAATGAAAGCAAAAATGGAAAGTGGAGCATACAAGGAACCTTCAGATTTCGAGCATGATGTTCGTTTAATGCTCAGGAATTGTTTTCTTTATAATCCAGTCGGTGATCCGGTTCACAGTTTTGGTCTTAGGTTTCAAGAAGTTTTTGATAGACGATGGGCTGAACTAGGTGATTCGAGTTCTCGTGCTTCATCAGTTGCACCTCAATCAGCTCCGATTGCTCCAACTCCGAAAGTAGCAAAATCAAGTGCTCCAAAAGAACCGAAAGAGTCTCGAAAAGAGCATAAAAAGGAGACGACTTTTGAAGCAAGCGGTGCAAAATCGGAGGATTTAATGCAGATAAACAACGCGTTGAGCATGATTCGAGAACGTGAGGAAAAGCTTAAAGCAGAGCTCGCCGCTGCACAAGCGATAAAGGATAAACTGACGAGTGTGAAGAATCGACGAGAAGATAATCCGAATGAGCCATTTCCGGAGAAGCTTATCAATGAGACAAGAGCCTTGTGCACGACGCAAGTTGGACAAAATGCTTCAAGTTCTTCAGCTTCTTCTGCTGCTTTGAGGAACGGACGAAGCAAAAAAGCAGCATCCGCACGTCTCTATGGTTACGAATTTGATTCGGATGATGAGGATAATAAGATGGCACTGACTTATGAGGAAAAACGAAACTTGAGCAATCTGATTAATAATTTACCCAACAATCAACTCAACACCATAATTTCGATTATTCAACGGAGAGAACGAAGCGCTCTGATGCAACAACAACTCGATGACAGTGAGGTTGAACTGGATTTCGAATCACTTGGAGATATGTGCCTGAGAGAAATGGGTGCATTTATCAAAACAATTCCAACATTAAACGGAAATGGCGATGATGAGAAGCCGAAAACGTCTTCGAATCCGACATCTTCTGGAGCAACAGGATCAAAGGGTTCGTCGTCGTTGGAGAGCAAAAATGGAAAGAAAAAGAAAAACTTCAATATGTCCGAATCCTCGGATGATGAGACGTCGAATAGTCGAAAACGTCGAAAGAGAGAGAGCAGTGAATCACAGAGCTCTTCGTCCAGTGATGATGATTCAGATGATGAGGATAGGCCGAGTATTCCCCGTAAATCAGGTCAACCACCATCAACATCACGTGAATGGAATCAATCATCAGCTCCTCCACCACGAATGGGAGGAATGGGAGGACAACCACCAATGTCACGAGTACCTGCATCATCATCCACATCTGTATCAGCAATCGGAAAGAACAACGCAGCCGCCTCGTCGAATTCATATCAAAAATTTTATAATTGTTTTCACAGTTATACTCCACCTTTAAAAGTTGAAAAAAAAATCATCAAATTACTGGTAAATTTTTGTTAA.

#### bet-1p

The sequence for *bet-1p* was as follows: CACAGGTCTCTAGTGTATCCACTTCGAATGCGATGCCCGAAACCTCTTCATCCATCCGTCTCCTTCTCGCTCTCTCTCTCTCTCTCTCTTCTCCATCTCTCTCCACATTTTGCCTGCTATCTCGTGATTGTCGTCCCGTCCGTGTTCCGCCGCACACACTGCCTGTCTTCTCTTAACCGTGTGTCGATCAACTCCCAAACCGCTACGCTATTTCTCTCTCCCTCTCTCTCTCTCTTCGGCGGTGACATTTCTGACTAGATGGTCATACAAAACGCGTGCTGCGCGCGCGCTCCGCAAAAATCGACGCGAATCGATTAATGTGCGTCTCGTTTCTCTATCTCTGACCGCCCCCGCTTCAACCTAACACTATTTTTGAATGCTTTTCAACTGTAACTTGCAGCTAATTAGAAGTTGAGAGATAACCTGTTGCGATTGGCTCCGGGCAAGGGTTGGGAGGTCGCACCAGAAATTTTAGAGCTCTAGGATTTCAAATTTTTGGGTTTCAAGACCGTAACATGATTTTCTTGGAAATTTATCACAAATCATGTAGAAAATCGATATCAGTAAGAGGGAGTGAGTGATCTATCATTTTTTATCTTTCGATCTGAAATTCCACAGCGAAGGTTTTCTGCCGAAATTTCGAAATTGGTATTTTGAACTATCCGATAATTCGTAGAACATCAAGATAAAGTGTCAACCTATAGAAAATCACATGATTCGTCAGAAAATAACATTAATTTCATATGAAATAGTTGAGAAAGTGCTCAAAAATGGCCTAAAATTATCCAATAATCGACATTTGACAACTTTCAGCACACTTTTGAACCGTTTATCAATTGTTTCTGCTGAAATAGACGTATTTTTCGGACGAATCGAGTGATTTCCTATAGTTTTACACTGATTTTTGACAAAAAAATATTGATAGAACATGGTGCATTAGGCAATTTTTTAGAATTGCCGTCTACACCTGATTTCGATGGGTCCTCGTGACAAGACCCAAAATTTTATTATTTTTATCGTTGAAAAAAATCAAATCAATAACACCGCAATCACCATTTGCAAAGTTTAATTAAATACAATTTTTATTAAAATATTTCAGGAATAAAAATATTAGTCAGAATAATCCCATGTTCTTCTAGCGATTTCAACTAATTCTTTGAAAATAACATTTCTTGGAATTTAAGAATACGAAAATAGTCACTTCTTTGTATTCTAGAAACGCTAATTCCTGCAACCGACAAATTAAAAGTACAAAAAATGATACGGCAAGCGCGCTCCAATTCAAATCGAGTCTCCCGCCTTCCTTGACGTCATTGCTAACAGCTGCTTCGGTTTTTTCCTCCAAATTTCGTGGTTCAAATTTTATTTTTAATTGAATTTTAACAAAATAGGAAGCTAGTTGAGTAACATTTATTATTAATTTTGTAAAATATTCTGCAAATTCGGCGTTTTCTTTTAATTCAAATAAAAGTTTTCAATAAAAAAAATCGATATTTTCAG.

#### col-19p

The sequence for *col-19p* was as follows: ACGTACCATTATTCGAGACAACTGTGAGTATAAATTTTTGGAAAAATATTGGATAGTGAAGCTCATAATATTCAACAATGTTTGTGGCTGCTTCTGCAATCTCATTCAACTGAGCTTTCATTGAACTTGAAGATCTGATCACAAAAGCGATTGCACTCTGAGCTTCATCAAAATTGTCGTCCGATTGTTGCAGACAAGAAATATGCTCACAATGAGCACCACTGTACCCAACATCACAAATGCAGCCTGCGTTGTCTGGAGTCGTGGTTCCGCCATTGAGACAAAGAGGAATCTCGCAGTTACCGCCGGTGAAAAATTCGCCACAATAACATCCCTGAAAAAATGTAGTGCTATTAGTAATCTCATACATATAAAGATGACATTTTCCTACATATGTATAAAAATTGCAAAAAGTGAAACTTCAAATATCCATATTCTAAGACTTTTTTTCAATCTGCTCCAATTTCTCAAATCTGTTTAAATTTTTGACAAATGTTAATCAGTTTTCGGCTATTTTTGATGTCACTTGGTCTTAGAGCTTATATAGCTTAACAGTCAAAAAAGTTACGCACTTGGAACAAAAAATGCTCTAAAATTTCCAGATAAGAATTGGAAAGGTTTTGATTTGTTCCTTTCATGAATTGTGGTTTTCAATTGTTTGTTTTAAAACTATTTTCAAAAATGCAGCTTGCGATCATTCATGTGTTGTTCGTAGTTTGTGACGTCAATTTTAAAATCTTCAAGAAATACTTGCATTTCTATAAAATTGATTTGAAATTTTGAAGCCTAAATTACTGTCAAAAATTTTGATTCGAAGGTTTTCTAAAACAATGAGTGATTTCGGGCGTTATATCAAATATCCAAATATATAAAATTTCATAAAACTACTATAAACTATATGCTAGAAACTCGTATTTATACTAGAAATACTAGAAACTAGAAACTATAAAAACCTGGAACATCAAATAAATTTAATGTCAGACTACAACCCTTTAAACTACATACCAACGATTTATTAGTTTGAGCTTCAAGAATTTTATATTCTTAAGAGGGACGCACAAAAAATCACTCTTCACACTATTTCTTTGTTGAATAGAGGTCAGCGGCTTTTAAAAATTATAATTTGAAAAAAAATTGGGACTTTTTCAAAAAAAAAGCTTCCAAACGTCCCTATTAGGAAATGTAAACTTATTCCCAAAAAATTAAAAATTCCAGAGAAAGTAGACAAATTTCAGAAAACTTACCGCGCTACATCTAATACTTTCTCATAGGTTTTTTTATTGGGAAACTGATGAAAATTATTTGAATTCATAATAAAATAAATTCGTATTTAGCATTTGAAAATTTGCACCAATGTATTATTTAATTTTTTTTTTCGAAAATTTAACGCATTTTCTCTCTAAAAACTCGAAATTTAGTGTGTTCTAAACAACAGTAAGCATACAATACCTTTGTTCAAAATTGACGTGCTTTCTGAACCAATATGGTTAGTTTCAAAAATTTTTGTATTATAGGATAGAAATATTTGGAAATAAATTTTAAAACCAAACTTATGCCTTTCTCTTTTAGTATCCCAGCTAGGTAATATTTTAGTATTTGCCCAAATCCTTGAAGTAAGGAGTATATAATTTTTGAAAAACAATAAAACTCCAGATAATTCATAGTTTTTTCTCGAAAGAAAATTTTTGAGATTAGTTATTGAACTTCATTTTTGAACATTATTCGTTGAAAAACACTCGCTTTGTCTTTATTTTCAAAAAAATTCCGATTTCCCCAACCAGAAAAAAAAACAGATAGAAGAAATTTCTCCTTAAATTTCATTGTCCATCTCTCTTGGAAACACATTATCTATCAAATGAAAAACGCATTTTTTTTCCTGGCAGAAAAATGAAATTGGTTAGATTACACTGGTTAGGTTTGAAGGTGTAACTTTCGCTTTCTCAGCAACTTTCAGTATAAAAGGAAACGGTCACCATTTAGAAAGACATCAGTTCATCAAC.

#### gly-19p

The sequence for *gly-19p* was as follows: CAAATATTCTCATTTCAAAATTTTCAGAAACGACCGCCGATTGATTGGGGAAGCCACGAAATAATAAATTCAGCATATGATTGTCTGGAATTTCTGAGTCACTTGAAATCCGATTGGAGATATTTTCAATATTTATCAGGAGTTGATATTCCATTAAAGACAAATCTTGAAATGGTTCAAATATTGAAACATCTGAATGGAACTGCAAATGTCGAAATCAAACCATATCAATATCAACGCCTTCGAGGTAAAAATGTGAGTTTTTGAAATTAGTTTTATATTCTCAGTGATCATTGCTCAAATTATAATGACTATTTGCAATTAGGTTTTTTCCAGAAAAAGTTTTCTTTTTGATTTGGTAATGTGCCAGAACTGTGACTGAGAATTGTTGAAAACCCCAGAGTTTTTTTTTAAATTTAAATTGGCGCTACTCCACAAGCTAAACAAAATTTATGTAAAATCAATAATCCGATGAAACATCATTAAAATTATTTTCAGGAGACTCAGTCTCCACTGCCGCTTTTTAAGTCATCACTGTCATCACTAATACCAAGAGAAGCTGCCAATCACTTAGTGAGTTACTGAATCAATTTTAAATAATAAAAATATCATAAAATCATAATTTCAGTCATCATCAAGCATTCCTCAACAACTATTGGAATTTCTTCGAAATACTGGAATTGCTGATGAAGGATTTTGGGGAACTCTTTTTGGAAACAAGAACTTGTTTGATATTCCTGGATCTTTGAATTTCAAAGAATGGATATCATACAAAAATAATGTAGAAACGAACTTAACATATCCCACTGATGGATGGAGATATTATATATCCAGAGATCAAATATGGTCAAAGCCCAATTGTCATAATTACATGAAAGCTGGGTCATGTGTTTTTGGAATTGGAGATGTCCCAAGATTATTGAAGTCAAAAGCGTTGGTTGCTCATAAATTTTACCTAAAATCAGAGCCAGAAGCATATTTTTGTTTATTAAAGGTTTGTGCAATTTTTTTTGGTTTGAATATATACGAGAGTTTTAGAATTTCTATGGGTGATTGTCTGTCATAGAATTTGTGAGATATGATGAAAAGCGAATTTCCAAACGTCAATCAGTTTCTGAATTTATATATATATTGTTTTTTAGGAACACCGTCGCCGAACGTGTAATCCCGATTTAACATTTGATGCATCAAAATACTCAGAGTTACCGCAAGTTGAACTATCACGAGGTCTTGCCATGCCACAACTGACACATAAAAATTGGATTTTATGATTTTTTTCAGTACATTTTTTCATTTCGGGTTCATTTTTTGGGTCAACGTTTCCAAATTGTTTTGTTTTGAAATTTCTCAGGGTTCTCATTTGTTTTAGTTAATAATTTCATGTTTTTATTTATGAATCGAAATAAAATTAAAACATGTTTTAAAATATTTTCTGGTGGATAAAAAAGTATTTGGGTAAACTGGAAATTATTTTGTATGTTGTTTGAGATTTAAAAAAAATCTGAGACAATCGAAGAAAATTTGAAACATTTATATTTTGATTAAAGTAATAACTTCAATCTTCAAAAAAGGGCATTAAAAACAGTTTTTCCTAGGTTTTTGAGTGCAAACATACGCTTATCAAAAAAAAAAAATCAAAGGAATATGCCAACTGCAAATATACTTATCGTATCTTTTAGAATCGAAGGTTGGAGAGAAATATACAATTTATTATATACCAGGGGATTATATTCAAATTAACCTTTTATCATAATTTTTAATATTTCTCAATTCGGTTATAAATTTGAATTTTAAAACTGTTTTCAAATGCATAATTTTCAATTGATACGATGAATGTGTTATCAGTATAAACAATCCGAGTCATTCACCCTCTTTTCCTTTTTCTCTATGAAATTAAAGACATTTTATTAATATTTTCTTCATTGAGAACTCTCAATTCTGTTAAAACAGTTTTCCGGTATTGAATACTAAATTCCAAAGAATTAAATTTAAATTTCCAG.

#### myo-3p

The sequence for *myo-3p* was as follows: TGTGTGTGATTGCTTTTTCACAATCAGTGTTTTCAGGATTATGTGATGAACTAGATCTTCAAGTTTCGTTACATTTCATATGTTTTCGGAACTCACGAAGTACATATTGGGTATTGTGCTCAAAAAATTCAGCAATCAGCTTCGCTCCGCTGACTTTAGAACCCAAAAAAATAGTATGGCCAAACTGACTGTGTTACGATCATTTCAATTTTTCAATACATATTTAAGATTTCTAAGAGTAAGAAGGTCAAAAACTGTTCTGGAATACATATATATTTTTCAGGTTACAATTAGTCAAAAAGTGCACTGAAATATACGTTTTAATTTCACGAATAACCCAATTAGTTCAATGTATTTTTGGTCAACCAACGTTAAAGTTTGGCTTCCAACCAATTATCATTTCTGATCAACCACAATGTTTTTTCTTTATCTGCAAGTTAATTTTTTATTTTTATCCAGATGTTTGGCATATTTTTCAATTCTTCACTAGCGCCCACTTCTTGCACTTCCGGCGCCCTGAATCTAATGCATCTGTTGCAAGAATTGAAAGACCAATCAACACATTGTTTTCTTCACGAGATACTGAAGAAAATGAATAAAAACAGAGAAAAAGAGCCATGTGATTAGTGACAACTGTTGCTAACAGATACCATAGCTTGGACTTGGTACGTGATGGCAACGTATGGGTCAACAAAAATGATTGCAGAGGGGGTGCAAAACAGTCAAGTCGAGAAAATATGAAAAACAGAAAACAAAGAACAGAAAAATGGGTTTGAGAGTCAGTATAATTTATAAAAGAAAAATTGTACATAGAAATTAACCATTTTTGTAGAAGAAGTTATTTTTCAAGCATCGTTAAAAATTATTCAAAGCACCTTATTTCATATTTAATTTTAAACATGGTTAAATGAACAACACGGTGCGCAATCAGGAAAACTTGAAATCTGAAACTGTTGTTGTGATCTTCTTCGCAACTGTTCAGATAGCACTAGTGTAATGTTAAGAGTGCGCGAATATAATGGAATATAATGGATCACACCTCCTGCCATCAGGTAAACGTCTCTGTTATCACATATTTCCAACTATTAAATTTTTACCTTTTACAGTTTTACATTTTTTTGAAAAAAGTAACTTTTTGTCTTCAAAATCCCTGACGAAAATATCAAATATTTTAATCGAGACTGCAGAGGAACCGATTGATGATTTGGAAAATCCAGCTTTACCTGTGTAAGAACTGAAAAGTTTCATAACCCTAGGGTATTCCCAGTTACATTCCCCACTGGCTAACAATAGCACCCAGTTTTTCATCACCTTTCTTCAAATTTCTCGGCGATTTGTTAAAAACAAAATTTGTGTCCCTTCTCTGATATCTCTATGTCTCTAAACACAAGTTCATCGGAAAACGAAGGAGGGTAGGTGTTGGTTGGGCTCCCGAAGTGAAAATAGAAGAGCAAGAATAGAATATTAGAGAGAGAGTGCAGAGAGGGCGGGATAGCTCCCGGGATTCCGTTTTCTTCTTCTTTATCTTCAACGATGATGTGTGTGCGTGTTGTATAGATTCTGTTGCTCCCCCACAACTCGCTCCGAAGGCTCAATACAATTCAATTGATATTGGAGGAGAGCCTACCGGAGTGGGAGGATAAGAAGAAACATAAGAAGAAGAAGAAGAAGAAGCATGCTTCTGGTTTTTGATGCTATGAAAACGGCACAAAAAGATGATTGAGGTCCCTTTTCAATACCTTCTCTCATCTTTCAAATCCCATTGAAACCTAAAACTTCTCACCACGCTTTACCATTGTTCTCCAAAAACTTATAGCAATGTCTATAACTTTTTTATCTCTGAAAAGCAGTGTTCCATTTTTCTTTTTCCTATTTTATTTCAATTGTTTCTCACATTTCGTTTGGATTCTTTGCTTGTCAACCAGCTTCTTCTTCCACTTTTACCGTCTAATTTTCAGGGCAGGGAGCCATCAAACCCACGACCACTAGATCCATCTAGAA.

#### rgef-1p

The sequence for *rgef-1p* was as follows:AAGACTAATTTTCGATTAACCCGTAGGGGTGCAAGACTAATAGAGACTGCAAGACTATTAGAGGCTGAAATACTAATTTTCGTATGCTCAATAATTTTGGAAATTGGCCTATTTTTTGTAGAAACTTGATACCGTTTAAACAAGGAAAAATACACACTTTTTAATATTTCATCAATAATTTGAACGATTTTGTGATTTTAAGTTCAATTTGCCCAAAAAAAGACAATTTTTCTGACGTTACCGCAAAGTAATGCTGGTCAGGCAAAAAGAGCGGTGCAAAAATATAAGAGACTGCAATACTAATAGAGGAAATACGGTAATTGAATTTTAGTGAAACTTGCGACAGTTTTCCTCATTTTTTTGTTATTCCGGCATACGAGTGTGGCATACGAGTGAGGTCATCTTTTGTTTCTTCCGTTTCTTCATCGCTTTTGAAAAAAAATGTTGAAGAAAACTTCGTAGCCGATAGCCGGATAATGTTTGGCACGGCGTTCCAACATATATCATTGGAAATTTTTTAAGATTTTTGCGAAAAATCACATTCTTCACGATGAGAACACGTTATTGAAGGAATATAAATCAAGAATAACATATAGTTATATTTCTCTATTACTTTAACGTTAAATATGAGCAAATTTGAGCATTTTGATTGCGATGAAAAGCAGAATCGAGTCAACTGAAATCCGTTCAAAAACCTAAGCTCGGTTCGCCCGAAAGTCGATTTTTCCAATAGCCGAACAGCACTGCTTCTTTATTTTCCAGGTATTTTTGGGGTGTAAGAATTATATCTTCTTTGTTTGGGTATTTACGTTTCCGAATTCCCTTTCTTGATCTGTCGCTATTTTTCAGAAACCAGAACAGTTTATCCTTTTTCATGAGAACCTGAATAGCTCAAAAACCCGTTTAATTTCTTTTCATCATCTCATTTGAAACTTTCCTAGTCTTCTTAATGATTTCCAGGCTCCTCCTACATTTGTATCTCAAGATGCATACACTTTATTTCCGCTCCAATTCGAATCATCTACTTCTCTTTTTTCTTCAACTTTCTACTTTTTTCCATTCTTTTCTTTCAAATGTTCGACGTCTTCAAGTGACTGCTCTTCATTCTCCTCATTTTTCTCGAATTTCATTCTTGTCTCTTTTTTGCTCAAAAATGGAAAATGAAAGTGACGTGAGATTTGGACGGCGGGACACGGGGGCAGTAGAAGCAGCAAAAAGGAGAGAAAGAGGACACAAATAAGAAGAACGAATTCAAAAATAAGCGGAGAGGAGCTATTTCCGTCAATTCTACCTCCCCAATCTTCATCAATTCGGCTCAATTGAATGACGTCACAGGGAGATAAACCGTTTGGATGAGCGCCGTCCATCACTGACGCCATCCCGTTTGGGACAAGAAAAGAGAAAAAAGAGCACAAAGTTTTTGGTGACGGATCTTGTCAATCATATGAAAGTTGTTCTGATTGATTGTCAGTTTTTTTCCTACTTTTTTGGATTCTATCCACTTCTGAACTTTTGACAAGTTTCAAACTTTCTGAATCATTTTCTATGCATTTTCCTGGAATTCTTTTTATGTAAAATATGAAATAGAATGTTTTTGAATTCAAGTTCTGCTTTTTTCTTCTTTTTTGTTCTGTTCGGGCTTGGGTATGCTTTTTTTAAAAATTATTTTGCACATCGACCAATAAGTGCGCAACTTATAAAATTAATTTATTTTTGTTAATTTTTGAAATACTTGTATTGCTTTAAGTGATCTGACCTCGCCCTGAGCTTTCCACGTAGTTATCAAATACAAATCCTCCAAGGGTAACGTACCTATATTACTGATCTTTATAATAACTTTATCACCTGTCCAGTTCCAGAGGAATTCTGTTAAGCTTATAGGCACAGAAGGAGTCATTTCTGCTGGTTTTTAATGATCCAAATCTTTATTTCAAGTAAAAAACTGAACACTTGCGAATAAAACTATCAGATTAACCATTCACCAAAAATGTGTTTGAATCTAAAACTTTCTCAGTATTCCAAATATAGAAATAAATAACCACGACATTGCTAAAATCTGTCTGAATTGTGTACTCCTTACCGTGAAGTAATAATGGATATAATGAATCGTTTGAAATGAATGATCAGCACATTTTTGGTGAAAGATCACAAATAAGGAATAAGCGACGGAAAATAAAACGATTATTTCGGATCAAAAATTTGTTGAAGATCATATACACTCGAGACCAAGATTATTCTAGACAATTTTCAAATTGGCTTCTTTGTTTGCAAATCTTCATAATAATCTGTGAAGTTTGGAAATTTGAATTTTTAATCTTTTTTCATAGATTATAGTTTTTATTTCTTTGCAAAACTATATTAAAAACCGATGCATTGTTTTAGGGAAATTAATGAGCCTTTTGTTCAACACTAAAAACAATAAAATTAAAATTTTGGCTTCATCATTTGACCTTTTTTAAGTTCGAAAACTTTTCTCGTATTTCTGAACCGCCAATTTTTTCACACATCTCTAGACTTTTGGTGCCCGTTCCAGAAAGTTAAGTAATTGCTATTCTAAGAAAGTTCTCAACATTGTTTTTTAGTTCTGATTGAATTCTGATGTTCCAGGAATATATTTTAAATTTAATATTCTTTGCACTATTTCTATACTAACTAAATAATAAATAGTCTAAGTATGTTCAATGAGAACAAAAAAGCTCTATCTATATTTTTCTATCCTATTTCATTTATATCCTTTTCATTTTGAACTCACCAGCTTCTCTTCTTCTTCTTGTCCATATACTTCTTAAAAGTCTCGAACTTCTCTCTCTCACTCTCCATCAATTTCTTTATGGCTACCGCTTGCGCTCTGCCGCTTCCGAAGAAAGAAGGCGTTGGCAGAGCTCTCAATTCTATTTTTTCTCGCCGTAGGCTATCGATTTCTCAGCTTCTCTTCTCTCTTCCTCACTCCACCACCACCACTTCGGGCTTCTTCTTTTCTTCATTTTCGTCTTCTTCTTCATCCTCTTCTTTTTTTTTCAGTTCCCCCTCACTCCTCCCCTTATATGCGCGTGCGAGAGGGGTGCAAAAGCAGCGCGCATCCGAGAATTGAAACGAGAAAACGGAGACGCAGCAGCAGTTCGTCCTCAGAAAAATAGCCAGTAAAAAGAGAAAAAGATAGAGAGAGACCTATCGCATTTTATTTTCAAATTGCAATTCCTGCAATTCATGTGTGCGTGTGCCCATTTTCAATTCTTCCCGTTATTTTTCAGACCATTACCAACGTTCTTTCTAGATCTTGAGACAATCTTCCTTCTGCTCAATCGTTCGTCGTGAAGACGGCATCGACGAC.

#### *unc-54 3*′ *UTR*

The sequence for *unc-54 3*′ *UTR* was as follows:CATCTCGCGCCCGTGCCTCTGACTTCTAAGTCCAATTACTCTTCAACATCCCTACATGCTCTTTCTCCCTGTGCTCCCACCCCCTATTTTTGTTATTATCAAAAAACTTCTCTTAATTTCTTTGTTTTTTAGCTTCTTTTAAGTCACCTCTAACAATGAAATTGTGTAGATTCAAAAATAGAATTAATTCGTAATAAAAAGTCGAAAAAAATTGTGCTCCCTCCCCCCATTAATAATAATTCTATCCCAAAATCTACACAATGTTCTGTGTACACTTCTTATGTTTTTTACTTCTGATAAATTTTTTTGAAACATCATAGAAAAAACCGCACACAAAATACCTTATCATATGTTACGTTTCAGTTTATGACCGCAATTTTTATTTCTTCGCACGTCTGGGCCTCTCATGACGTCAAATCATGCTCATCGTGAAAAAGTTTTGGAGTATTTTTGGAATTTTTCAATCAAGTGAAAGTTTATGAAATTAATTTTCCTGCTTTTGCTTTTTGGGGTTTCCCCTATTGTTTGTCAAGATTTCGAGGACGGCGTTTTTCTTGCTAAAATCACAAGTATTGATGAGCACGATGCAAGAAAGATCGGAAGAAGGTTTGGGTTTGAGGCTCAGTGGAAG.

### Wide-field and confocal imaging

Wide-field imaging was conducted as described previously (Kim et al. 2025a) on a Leica THUNDER Imager equipped with a 63×/1.4 Plan AproChromat objective, a standard dsRed filter (11525309), a Leica DFC9000 GT camera, and a Leica LED5 light source, all controlled by LAS X software. High-resolution confocal imaging of *gly-19p::LifeAct::mRuby* was performed on a Leica Stellaris 5 confocal microscope fitted with a white light laser source, spectral filters, HyD detectors, and a 63×/1.4 Plan ApoChromat objective, also managed by LAS X. Worms were placed in 100 mM sodium azide on a glass slide, imaged within 5 min to minimize sodium azide-induced artifacts.

Autophagy was monitored by quantifying GFP::LGG-1/Atg8 puncta in proximal intestinal cells, and body wall muscle of strains DA2123, RD202, KUM174, and KUM175 on day one of adulthood (Chang et al. 2017; Kumsta et al. 2019). Animals were grown at 20°C from hatching on NGM plates seeded with *E. coli* OP50 as a food source. For imaging and puncta quantification, animals were mounted on 3% agarose pads in M9 medium supplemented with 0.1% NaN_3_. GFP::LGG-1/Atg8 puncta were visualized using a Zeiss Imager Z1 equipped with an ApoTome.2, a Hamamatsu Orca Flash 4LT camera, and Zen 2.3 software. Puncta were counted in the one to three visible most proximal intestinal cells and in body-wall muscle cells at 1000× magnification. At least 10–12 animals were imaged per condition, with results pooled from three independent experiments and analyzed using two-way ANOVA (GraphPad Prism).

For imaging mitochondrial morphology worms were placed in M9 solution without 100 mM sodium azide to eliminate potential artifacts arising from the effects of sodium azide on mitochondrial integrity (Kim et al. 2025a). Here, HistoBond slides, which have a positively charged surface, were used to enhance immobilization and minimize worm displacement during imaging. Muscle and hypodermal mitochondria were imaged using a Leica Thunder microscope equipped with 63×/1.4 Plan ApoChromat objective, standard GFP and DsRed filter, Leica DFC9000GT camera, a Leica LED5 light source, and run on LAS X software. Intestinal mitochondria were imaged using a Leica Stellaris confocal microscope equipped with a white light laser source and spectral filters, HyD detectors, 63×/1.4 Plan ApoChromat objective, and run on LAS X software. The WLL was set to 85.00% maximum power, using a 485 nm laser line at 3.00% intensity. The HyD S detector was configured to 490–590 nm with an analog gain of 25. Imaging was performed using unidirectional scanning over a 1024 pixel × 1024 pixel area, corresponding to 82.01 µm × 82.01 µm, with multiple *z*-sections at a step size of 0.5 µm. For all images, the Z-range was set from the first detectable GFP signal to its end point. Mitochondrial morphology was quantified using the MitoMAPR macro in Fiji (Zhang et al. 2019). Z-stack fluorescence images were first converted into two-dimensional images using maximum intensity projection, and regions of interest (ROIs) were selected and cropped to ensure consistent analysis across samples. All images were saved in TIFF format prior to analysis. The MitoMAPR macro was installed by importing the source code and executed on the processed images to extract quantitative parameters of mitochondrial morphology. For batch analysis, additional macros were used to standardize ROI selection, perform iterative cropping, and process multiple images simultaneously. The resulting output data, including morphological metrics, were exported and saved as Excel files for downstream analysis. Each experiment comprised three biological replicates with one or two technical replicates per condition, and one representative image was chosen for the figures.

Neuronal dye filling assays were adapted from previous methods (Tong and Bürglin 2010; Zhang et al. 2021). Briefly, adult worms were washed from plates into M9 buffer and rinsed three times to remove adherent bacteria. Animals were then incubated in DiI solution at the standard working concentration of 1 µg/mL in M9 for 30 min at room temperature in the dark. Following staining, worms were washed three additional times in M9, transferred to standard OP50-seeded NGM plates, and allowed to recover for 30 min to clear excess dye before imaging. Worms were then mounted on slides in M9 with 100 mM sodium azide and covered with a coverslip prior to imaging on a Leica Stellaris 5 confocal microscope using the 547 nm laser line in combination with a restricted emission detection window selected to separate the DiI signal from overlapping red fluorescence from the *myo-2p::tdTomato* coinjection marker. Detector ranges were optimized empirically and then held constant across samples within each experiment. DiI uptake was scored at the level of amphid neuron pairs. In *C. elegans*, six amphid pairs routinely fill with lipophilic dyes. For each animal, the number of labeled amphid pairs was recorded and expressed as a percentage of the six total amphid pairs. Because worms were frequently imaged in lateral orientation, only one member of a bilateral amphid pair was often cleanly visible in a single focal plane. Accordingly, a pair was scored as dye-filled when at least one correctly positioned neuron corresponding to that pair showed unambiguous DiI uptake. Final dye-filling values were calculated as (number of labeled amphid pairs/6) × 100.

For BODIPY staining, age-synchronized worms were collected at the indicated day of adulthood, washed three times in M9, and fixed in 4% paraformaldehyde for 15 min in the dark. Animals were then freeze-cracked by liquid nitrogen thaw cycles, washed in PBS, and incubated in BODIPY 493/503 diluted 1:1000 from a 1 mg/mL stock into PBS for 1 h at room temperature in the dark with gentle rotation (Klapper et al. 2011). After staining, worms were washed three times in PBS and held overnight at 4°C in PBS protected from light prior to imaging. For imaging, animals were mounted onto slides in glycerol-based mounting medium and imaged on a Leica THUNDER imager equipped with a 10×/0.40 NA UPlanXApo objective, a standard GFP filter, a Leica DFC9000 GT camera, and a Leica LED5 light source, all controlled by LAS X software.

For Oil Red O staining (Lynn et al. 2015), synchronized worms at the indicated day of adulthood were washed from plates in PBS containing 0.01% Triton X-100, briefly equilibrated in 40% isopropanol, pelleted, and then stained in Oil Red O prepared in water for 2 h at room temperature with rocking. Following staining, animals were washed in PBS containing 0.01% Triton X-100 for 30 min before imaging. For imaging, animals were mounted onto slides in glycerol-based mounting medium and imaged on a Revolution by Discover ECHO software version 2.0.8.1. Quantification was performed by drawing an ROI around each individual worm and measuring the red intensity using an RGB color camera scale. Data is presented as integrated red density normalized to ROI (i.e., worm) area.

### Stereoscope imaging

For full-body imaging of strains expressing *polyQ::YFP*, *dhs-3::GFP*, *sqst-1::GFP*, or *hsp-16.2p::GFP*, animals were imaged starting at day 1 of adulthood and throughout their life spans at ages specified in the figure legends. At least 10 worms were placed into 100 mM sodium azide (prepared in M9) on NGM plates lacking bacteria to induce temporary paralysis, arranged in a line, and imaged with a Leica M205FCA automated fluorescent stereomicroscope driven by LAS X software. The microscope was equipped with a standard GFP filter, a Leica LED3 light source, and a Leica K5 camera. Three biological replicates were performed for each image, and the figures all contain one representative image. To quantify DHS-3::GFP, *polyQ::YFP*, *sqst-1::GFP*, and *hsp-16.2p::GFP*, a region of interest (ROI) was drawn with Fiji along the posterior half of each worm group, and integrated density was measured. Statistical analysis and graphing were carried out in GraphPad Prism 10, using Mann–Whitney testing except where otherwise noted. For *vha-6p::Q44::YFP*, which produce robust aggregates, the number of visible aggregates per worm was counted, and comparisons were made with the Mann–Whitney test (GraphPad Prism 10).

### Electron microscopy

L4 stage *C. elegans* were picked to synchronize the population and grown for 24 h at 20°C to obtain day 1 adult animals. For old animals, L4 animals were placed on FUDR-containing plates (100 µL of 10 mg/mL FUDR). Animals were subjected to high-pressure freezing using an EM PACT2 (Leica Microsystems) followed by freeze substitution in an EM AFS2 (Leica Microsystems). Freeze substitution was performed (as in Stigloher et al. 2011) in 0.1% tannic acid and 0.5% glutaraldehyde in anhydrous acetone at −90°C for 96 h. Samples were then washed four times (1 h each) in anhydrous acetone at −90°C and fixed in 2% osmium tetroxide (OsO_4_) in anhydrous acetone for 28 h at −90°C. Following fixation, the temperature was ramped over 14 h from −90°C to −20°C (5°C/h), held for 22 h at −20°C, and then increased over 4 h to 4°C (6°C/h). Samples were subsequently washed four times for 30 min each in anhydrous acetone at 4°C. After washing, the temperature was raised over 1 h to 20°C, and samples were infiltrated with araldite resin through a graded series: 50% araldite in acetone for 3 h at room temperature, 90% araldite in acetone overnight at 4°C, followed by two changes in 100% araldite at room temperature. Samples were then polymerized in fresh araldite for 48 h at 60°C. Semithin sections were stained with toluidine blue to identify the animals showing long intestinal tissue portion and 70 nm-thick sections of the corresponding regions of interest were deposited on formvar-coated slot grids. Finally, the grids were contrasted by 1% uranyl acetate for 8 min and 2.6% lead citrate for 4 min. Images were acquired using a Tecnai G2 (Thermo Fisher), running an LaB6 crystal at 200 kV and equipped with a 2k Veleta camera (Olympus).

### Chemotaxis assays

Chemosensory ability toward volatile attractants was assessed at day 1 of adulthood using standard chemotaxis assays (Bargmann et al. 1993). Briefly, two marks were made on opposite sides of the back of an unseeded 10 cm NGM plate, ∼0.5 cm from the edge of the plate. At each of these marks, 1 µL of 1 M sodium azide solution was pipetted and allowed to dry, before the addition of 1 µL of test odorant diluted in ethanol to one mark, and ethanol, which served as a control, on the other. Odorants used (all from Sigma-Aldrich) included 0.1% (v/v) butanone, 1% (v/v) benzaldehyde, 1% (v/v) isoamyl alcohol, 10% (w/v) pyrazine, and 1% (v/v) diacetyl.

Day 1 synchronized adult worms grown on NGM plates were washed off their plates with M9 media into 1.5 mL Eppendorf tubes and allowed to settle by gravity. The M9 supernatant was removed and the worms washed with ∼1 mL of M9 buffer a total of three times before adding ∼100 worms at the chemotaxis origin, a spot equidistant between the two odorant marks, using a 200 µL wide orifice pipette tip (USA Scientific). Worms were allowed to chemotax for an hour at 20°C, and number of worms in a 1 cm radius of the odorant spot, ethanol spot, and the rest of the plate excluding a 1 cm radius of the origin were counted.

Chemotaxis indices for each time point were calculated as

$$\mathrm{chemotaxis}\;\mathrm{index}=(\#{\mathrm{worms}}_{\mathrm{odorant}}-\#{\mathrm{worms}}_{\mathrm{ethanol}})/[\mathrm{total}\#{\mathrm{worms}}_{\left(\mathrm{odorant}+\mathrm{ethanol}+\mathrm{rest}\right)}].$$

The percentage of worms for the rest of the plate was calculated as

$$(\#{\mathrm{worms}}_{\mathrm{rest}})/[\mathrm{total}\#{\mathrm{worms}}_{\left(\mathrm{odorant}+\mathrm{ethanol}+\mathrm{rest}\right)}]\times 100.$$

### *C. elegans* RNA sequencing

For all day 1 RNA-seq experiments, *glp-4(bn2)* worms were used to eliminate germline. After bleaching and L1 arresting, worms were grown for 72 h (i.e., 3 days) at 22°C to obtain day 1 adult animals. For day 7 RNA-seq experiments, we used N2 worms, as animals no longer have a functional germline at day 7 of adulthood. After bleaching and L1 arresting, animals were grown for 72 h (i.e., 3 days) at 20°C. Animals were then transferred to FUDR and grown for an additional 6 days prior to sample collection. Roughly 1000 worms per condition were harvested with M9, transferred to 1 mL of TRIzol reagent, then subjected to three freeze/thaw cycles between liquid nitrogen and a 37°C bead bath, with 30 sec vortexing between cycles. Following the final thaw, chloroform was added at a 1:5 ratio (chloroform:TRIzol), and RNA was separated by centrifugation in heavy gel phase-lock tubes (VWR 10847-802). The aqueous phase was combined 1:1 with isopropanol, and purification was performed with a QuantaBio Extracta Plus RNA kit (95214) per the manufacturer's instructions.

Library construction and sequencing were performed at Novogene using a paired-end workflow, poly(A) selection, first-strand synthesis, and Illumina NovaSeq6000. Three biological replicates were analyzed for each condition. Preprocessing to trim low-quality reads and adaptor sequences was performed with Trim Galore v0.6.7-1 (https://www.github.com/FelixKrueger/TrimGalore). Reads were aligned to the WBcel235 genome using STAR-2.7.3a (Dobin et al. 2013), and a raw count matrix was generated using Subread v2.0.3 (Liao et al. 2019). Differential expression was calculated with DESeq2 (Love et al. 2014) and sva v3.54.0 was employed to correct for batch effects and nonbiological sources of variation (Leek et al. 2012). GO enrichment analysis was run through WormEnrichr (Chen et al. 2013; Kuleshov et al. 2016).

### Lipidomics

For lipidomics, *glp-4(bn2)* worms were used to eliminate germline. After bleaching and L1 arresting, worms were grown at 22°C for 72 h (i.e., 3 days) to obtain day 1 adult animals. Animals were grown at 22°C for 168 h (i.e., 7 days) to obtain day 5 animals. Approximately 5000 animals were used for each replicate.

Animals were transferred into 2 mL,1.4 mm ceramic bead tubes (Fisher Scientific 15-340-153) with 500 µL of ice-cold 75% methanol (MeOH) in a 1:5 ratio of worms:MeOH. Animals were then homogenized using the FisherBrand bead mill in a 4°C room with a 1 min homogenization shake followed by placing the tubes for 1 min on ice for five cycles of this shake and cool down cycle. Following homogenization, methyl tert-butyl ether (MTBE) with lipid standards was added to the tubes in a 10:3 ratio of MTBE:MeOH and then vortexed on the benchtop to thoroughly mix, after which the tubes were gyrated on an orbital shaker for 60 min at room temperature (∼22°C). Next, to induce phase separation, mass spectrometry-grade water (MS H_2_O) was added in a 10:3:2.5 ratio of MTBE:MeOH:MS H_2_O and then vortexed on the benchtop to thoroughly mix before letting tubes sit for 10 min at room temperature (∼22°C). Tubes were then centrifuged at 20,000 RCF for 15 min at 4°C, and the upper phase was collected into a 1.5 mL tube, extracting as much of the layer as comfortable; lipids and lipophilic metabolites are present in this upper MTBE phase. These new tubes were centrifuged again at 20,000 RCF for 15 min at 4°C, and then 500 µL of sample was dried via vacuum centrifugation.

Analysis of lipids via liquid chromatography-tandem mass spectrometry (LC-MS/MS) was performed at the University of Southern California Alfred E. Mann Multi-Omics Mass Spectrometry Core (MMSC). The dried lipid samples were reconstituted in 60 µL of injection solvent (15 µL of 60:30:4.5 MTBE:MeOH:H_2_O, 45 µL of 1:2 IPA:ACN) and a 10 µL aliquot of each sample was injected onto a XBridge Amide HPLC column (4.6 × 150 mm, 3.5 µm; Waters Corporation) kept at 35°C using a 1290 Infinity II HPLC system (Agilent Technologies) and eluted at a flow rate of 700 µL/min with solvent A (10 mM ammonium acetate in 95/5 ACN/H_2_O) and solvent B (10 mM ammonium acetate in 50/50 ACN/H_2_O, pH adjusted to 8.7) using an optimized 28 min gradient (min/%B was 0/0.1, 6/6, 10/25, 11/98, 13/100, 18.6/100, 18.7/0.1, and 28/0.1). The column effluent was directed to an OptiFlow Turbo V electrospray ionization source connected to a QTRAP 6500+ mass spectrometer (AB Sciex LLC) acquiring mass spectra in a targeted scheduled multiple reaction monitoring (MRM) mode in both positive and negative polarities. In this assay, detection of fragmented ions originating from each lipid species at specific LC retention times were utilized to ensure specificity and accurate quantification in the complex biological samples. Lipid classes analyzed included triacylglycerides (TAGs), diacylglycerides (DAGs), sphingomyelins (SMs), ceramides (CERs), phosphatidylethanolamines (PEs), lysophosphatidylethanolamines (LPEs), phosphatidylcholines (PCs), lysophosphatidylcholines (LPCs), phosphatidylglycerols (PGs), phosphatidylinositols (PIs), and phosphatidylserines (PSs). The detection window was 200 sec, and a target scan time of 0.5 sec each polarity. Source parameters were as follows: curtain gas (CUR) 35 psi, collision gas (CAD) medium, ion spray voltage (IS) 4500 V, temperature (TEM) 550°C, and ion source gases 1 and 2 (GS1 and GS2) 30 and 40 psi, respectively.

The raw lipidomics data was imported into Skyline (MacCoss Laboratory Software) and checked for quality, utilizing a transition list with updated retention times for accurate peak picking and integration. An external standard (EquiSplash Lipidomix quantitative mass spectrometry internal standard, Avanti Polar Lipids, Inc.) was utilized for retention time determination. Chromatographic peak areas were used to quantify the relative abundance of lipids in each sample and to perform statistical comparisons between experimental groups.

Chromatograms were processed in Skyline Daily (version 23), where all peaks were integrated and manually reviewed to ensure consistent retention times and proper alignment of qualifier and quantifier peaks. In R (v4.3.1), peak areas were normalized to the total ion count for each sample and log_2_-transformed. Pareto scaling was applied for principal component analysis. Differential abundance analyses were performed using the limma package to calculate fold changes and *P*-values for age (day 1 vs. day 5) and treatment (control vs. *bet-1*) comparisons, with *P*-values adjusted by the false discovery rate method. All visualizations were generated with ggplot2 and pheatmap.

### Thrashing assay

Thrashing assays were performed on animals synchronized via bleaching and L1 arresting on day 1, 5, and 9 of adulthood. One-hundred microliters of M9 was added to the plate to float animals, and 30 sec videos were recorded with a Leica M205FCA stereomicroscope equipped with a Leica K5 camera and run via LAS X software. Over a 10 sec window of each video, thrashes were counted by eye, defining one thrash as a bend involving more than 50% of the worm's body in the opposite direction. The data shown represent a super-plot of all three replicates. Dot plots (generated in Prism 10) depict each worm as a point, with median and interquartile range indicated. Statistical analysis used the nonparametric Mann–Whitney test.

### Brood size assay

For brood assays, 10 L4 stage animals were placed individually on separate plates. Every 24 h, adults were moved to fresh plates, and plates with eggs were incubated for 2–3 days at 15°C. The total number of live progeny on each plate was counted, and these counts were summed to determine the brood size. Data is represented as a super-plot of all three replicates (generated using Prism 10) where each worm is represented as a single point; median and interquartile range lines are also displayed. The nonparametric Mann–Whitney test was used for statistical comparisons.

### Life span assay

Life span analyses were conducted on standard RNAi plates at 20°C as described (Castro Torres et al. 2022). Adult worms were placed on FUDR-containing plates (100 µL of 10 mg/mL FUDR) from day 1 of adulthood. Animals were scored every 2 days until all animals were scored as dead or censored. Animals were scored dead if they displayed no movement after tapping the head and tail of the worm lightly with a wire pick. Animals were scored as censored if they displayed bagging, intestinal leaking out of the vulva, crawling up the side of the plate, or other age-unrelated death and removed from quantification.

*E. faecalis* life spans were performed as previously described (Zhang et al. 2024). Briefly, animals were grown on standard EV plates until L4 and then transferred onto BHI plates seeded with 50 µL of the following mix: 5 µL OD_600_ = 3.0 OG1RF *E. faecalis* bacterial culture, 5 µL OD_600_ = 3.0 HT115 RNAi bacterial culture, 40 µL of LB + 1 µM IPTG. One-hundred microliters of 10 mg/mL FUDR was spotted onto plates 24 h after seeding bacterial lawns. Life spans were scored every 1–2 days (every day from day 3 to day 9 when animal deaths were the most rapid, and every other day for all other time points) until all animals were scored as dead or censored.

All statistical analyses for survival assays and life spans were made using the log rank test in Prism 10. Representative survival data are shown in the figures; all repeats are listed in Supplemental Table S1. Experimental conditions were blinded from researchers during scoring for at least one replicate per experiment.

### Fast kill assay

Fast kill assays were conducted as previously described, with minor adjustments (Mahajan-Miklos et al. 1999; Cezairliyan et al. 2013). PA14 was grown overnight at 37°C for ∼14–15 h. A 5 µL aliquot of the overnight culture was spread onto 3.5 cm peptone glucose plates (1% Bacto-Peptone, 1% NaCl, 1% glucose, 1.7% Bacto agar) containing 0.15 M sorbitol, using a custom open-loop glass Pasteur pipette as a spreader. The plates were incubated for 24 h at 37°C, followed by 48 h at 25°C, then seeded with 30–40 synchronized L4 worms and maintained at 25°C. Survival was monitored every 2 h for up to 8 h, and an animal was considered dead if it did not respond to touch. Three biological replicates were performed, each with three technical replicates (total of nine). Survival rates were plotted from 0 to 8 h, with 100% survival shown as a value of 1, and remaining time points represented as decimal fractions of the total population.

For food choice assays (Stuhr and Curran 2020; Nair et al. 2024), a single colony of PA14 or *E. coli* (OP50) was inoculated into 3 mL of LB medium and grown overnight at 37°C. The optical density at 600 nm (OD_600_) of each culture was measured, and cultures were diluted to an OD_600_of 1.0. NGM plates lacking streptomycin were seeded with 15 µL of *E. coli* OP50 bacterial culture placed as a dot at one end of the plate, and the PA14 was streaked diametrically using an “L”-shaped glass Pasteur pipette. Plates were allowed to dry and then incubated for 24 h at 37°C, followed by 48 h at 25°C. On the day of the assay, plates were equilibrated to room temperature, and 30–50 L4 worms were placed on the side opposite the bacterial dot. The numbers of worms located at the line, dot, or undecided zones were recorded at 1, 2, 4, 6, and 8 h. The movement index was calculated as the difference between worms at the dot and line divided by the total number of worms at both locations.

### Intestinal distension

NGM plates lacking antibiotics were inoculated with an overnight culture of PA14 and incubated at 37°C for 24 h, followed by an additional 48 h at 25°C as previously described (Nair et al. 2024). Synchronized L4 stage worms (45–48 h after L1) were then transferred onto the seeded plates and monitored after 8 h of exposure. Imaging was performed using a Leica Thunder microscope with Flexacam C3 color camera at 63× magnification with LASX software. The diameter of the intestinal lumen was quantified using ImageJ and plotted using GraphPad Prism. Each experiment included three independent biological replicates, and a representative image per experimental condition was selected for presentation.

### Data availability

All data required to evaluate the conclusions in this manuscript are available within the manuscript and Supplemental Material. All strains synthesized in this manuscript are derivatives of N2 or other strains from CGC and are either made available on CGC or available upon request. All raw data sets, for RNA-seq are available through Annotare 2.0 Array Express accession E-MTAB-15906. All raw lipidomics analyses are available through the Metabolomics Workbench (Sud et al. 2016) project PR002726 (http://dx.doi.org/10.21228/M89559). All raw data for all replicates are in Supplemental Tables S1–S6 and S8.

## Supplemental Material

Supplement 1

Supplement 2

Supplement 3

Supplement 4

Supplement 5

Supplement 6

Supplement 7

Supplement 8

Supplement 9
