# Supplementary material for: Neuronal expression of bromodomain proteins alters neuronal health and peripheral protein homeostasis to promote longevity
Source: Genes Dev. 2026 Sep 1;40(17-18):1523–52. doi: 10.1101/gad.353559.125 (PMC13532663; doi:10.1101/gad.353559.125)
Supplement: Supplementary file 1 [file Supplemental_Data.pdf]

Supplemental figures and figure legends

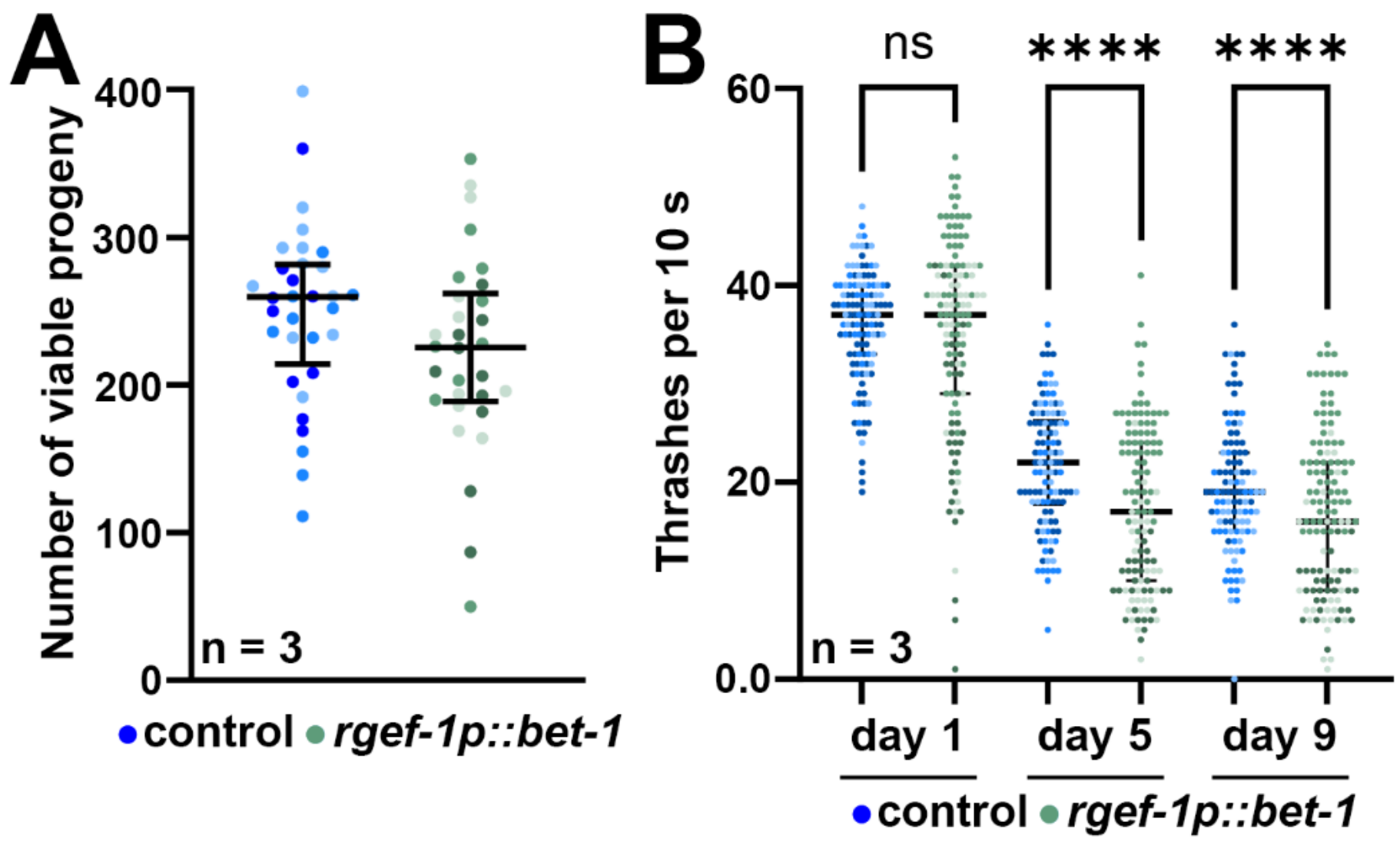

Supplemental Fig. S1. Neuronal *bet-1* has mild impacts on healthspan. (A) Brood assay measured by counting number of live progeny born by a single parent as described in Materials and Methods in N2 control (blue) and neuronal *bet-1* (*rgef-1p::bet-1, green*) animals. Each dot represents the total number of progeny from a single animal and lines represent median and interquartile range. No significant ($p > 0.05$) difference was found between groups using the nonparametric Mann Whitney test. Data is pooled across three biological replicates with different shades indicating each replicate. **(B)** Number of thrashes per 10s by N2 control (blue) and neuronal *bet-1* (*rgef-1p::bet-1*, green) animals suspended in M9 solution at days 1, 5, and 9 of adulthood. Lines represent the median and interquartile range. Data is represented as a super-plot of 3 replicates and different shades of color indicate a different biological replicate. **** = $p < 0.0001$; ns = non-significant, $p > 0.05$ by nonparametric Mann Whitney test.

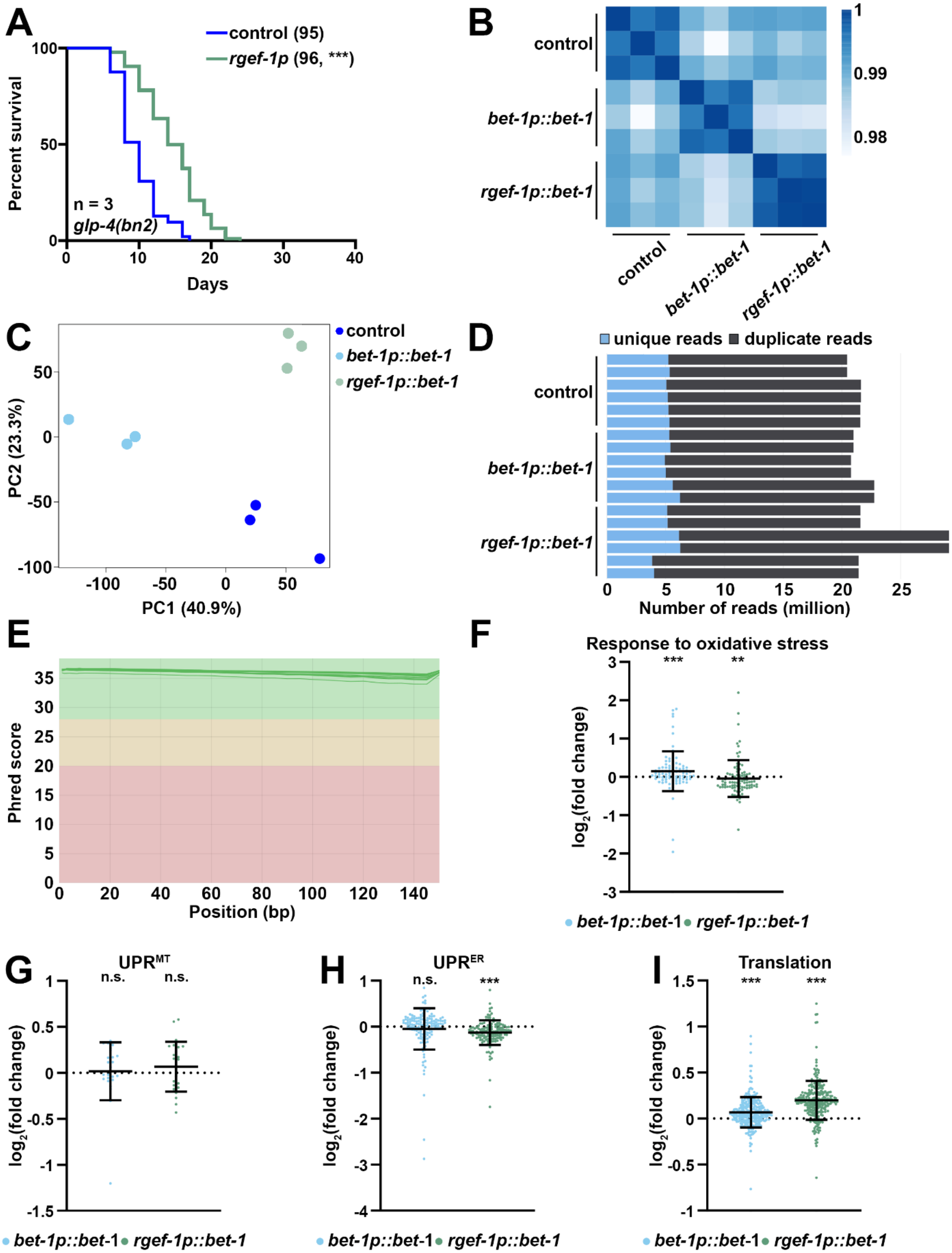

Supplemental Fig. S2. Neuronal *bet-1* alters gene expression related to OxSR and translation. (A) Lifespans of *glp-4(bn2)* control and *glp-4(bn2)* with neuronal *bet-1* overexpression (*rgef-1p*) grown on EV from L1 at 22 °C. **(B)** RNA-seq was performed on day 1

adult *glp-4(bn2)* control and *glp-4(bn2)* with whole animal *bet-1* (*bet-1p::bet-1*) or neuronal *bet-1* overexpression (*rgef-1p::bet-1*) grown on EV from L1 at 22 °C. Spearman correlation plot of all RNA sequencing libraries (n = 3). **(C)** PCA plots of all RNA sequencing libraries. **(D)** Mean quality score (Phred score) of each RNA sequencing library. X-axis indicates the base pair (bp) position of each sequence and the y-axis indicates the Phred score. The graph was generated by MultiQC(Ewels et al. 2016). **(E)** The number of unique (blue) and duplicated (black) reads from each pair-wise sequencing library (n = 3). **(F-I)** Gene expression changes in groups of genes related to response to (F) oxidative stress (GO:0006979), (G) UPR^{MT} (GO:0034514), (H) UPR^{ER} (GO:0030968), and (I) translation (GO:0006412). Each dot represents a gene and data is presented as $\log_2$(fold change) of whole animal *bet-1* (*bet-1p::bet-1*, light blue) or neuronal *bet-1* (*rgef-1p::bet-1*, green) compared to *glp-4(bn2)* control animals. Significance was determined using a one-sample Wilcoxon test. * = $p \leq 0.05$, ** = $p \leq 0.01$, *** = $p \leq 0.001$, n.s. = $p > 0.05$.

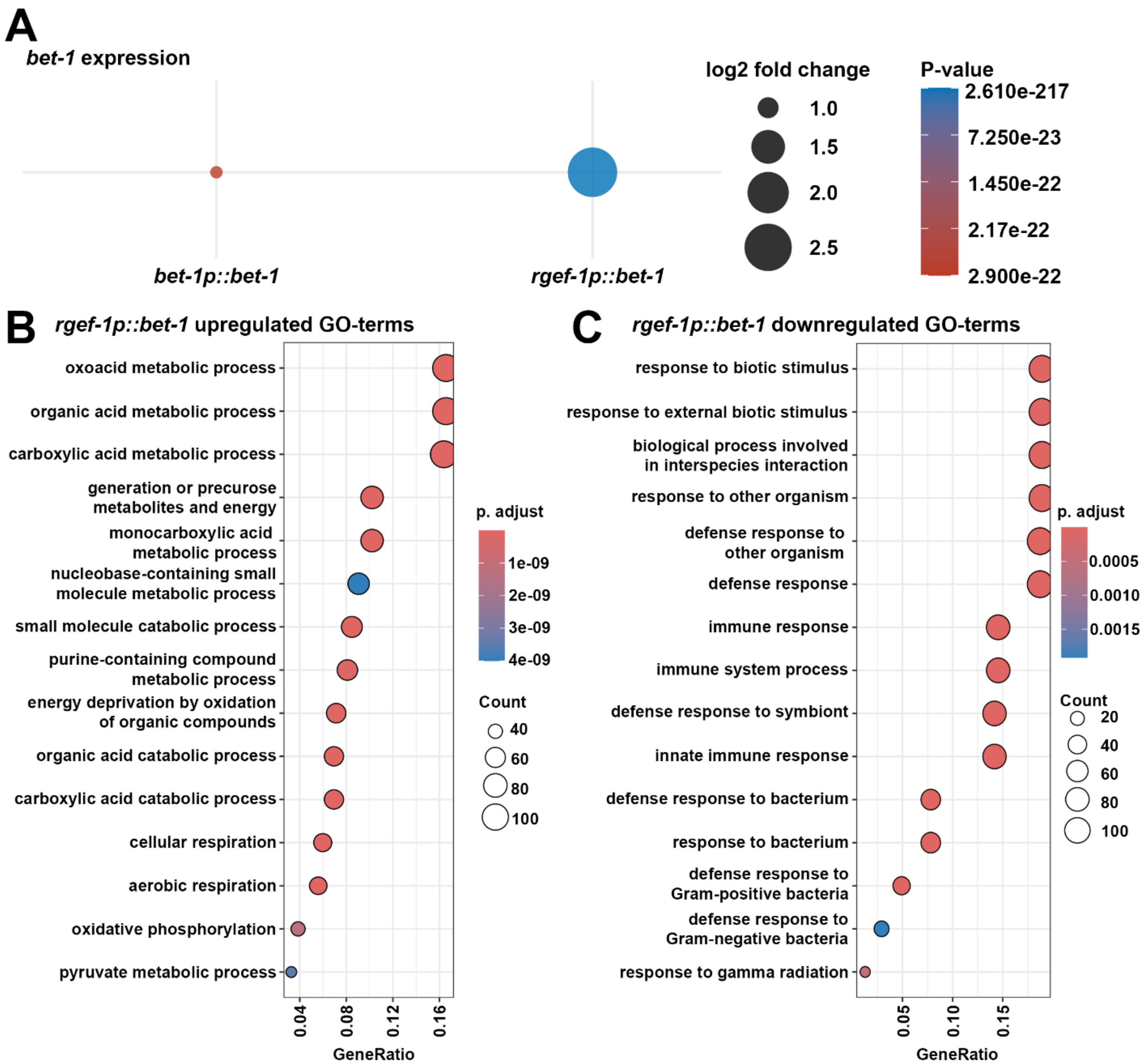

Supplemental Fig. S3. Neuronal *bet-1* transcriptionally activates pro-longevity pathways. **(A)** RNA-seq of *bet-1* in whole animal *bet-1* overexpression (*bet-1p::bet-1*) and neuronal *bet-1* (*rgef-1p::bet-1*) confirms increased expression of *bet-1*. **(B)** Top 15 gene ontology terms from the significantly upregulated genes (p<0.01) in neuronal *bet-1* (*rgef-1p::bet-1*). **(C)** Top 15 gene ontology terms from the significantly downregulated genes (p<0.01) neuronal *bet-1* (*rgef-1p::bet-1*). The list of all gene ontology terms can be found in **Supplemental Table S3**.

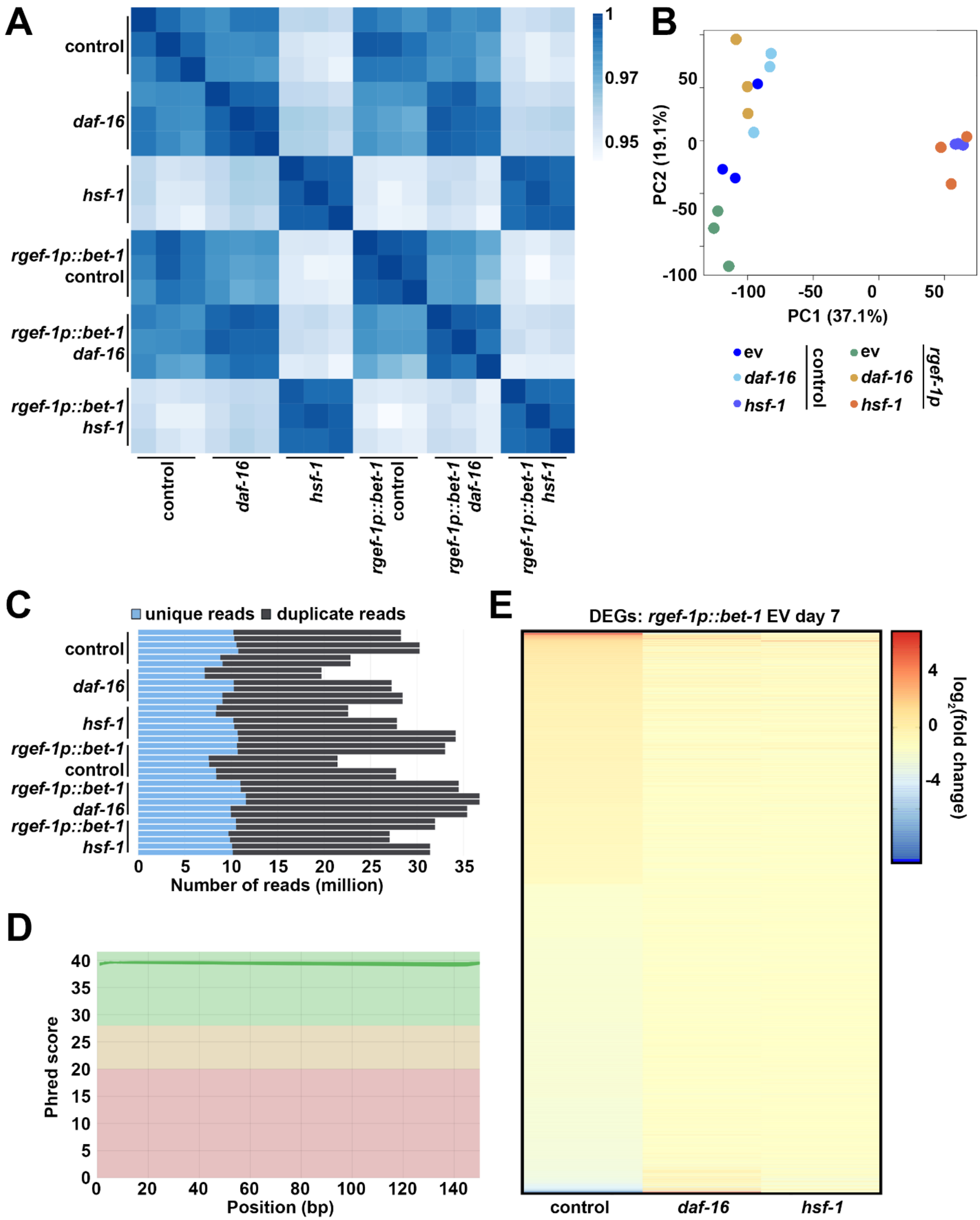

Supplemental Fig. S4. RNA-seq of neuronal *bet-1* performed at day 7 of adulthood. (A) RNA-seq was performed on day 7 adult N2 control and N2 with neuronal *bet-1* overexpression (*rgef-1p*) grown on EV (control), *daf-16*, or *hsf-1* RNAi from L1 at 20 °C. Animals were moved

onto plates containing FUDR at the late L4/early day 1 stage. Spearman correlation plot of all RNA sequencing libraries (n = 3). **(B)** PCA plots of all RNA sequencing libraries. **(C)** Mean quality score (Phred score) of each RNA sequencing library. X-axis indicates the base pair (bp) position of each sequence and the y-axis indicates the Phred score. The graph was generated by MultiQC(Ewels et al. 2016). **(D)** The number of unique (blue) and duplicated (black) reads from each pair-wise sequencing library (n = 3). **(E)** Heat map of all DEGs in neuronal *bet-1* (*rgef-1p::bet-1*) grown on EV (control) compared to N2 control grown on EV. *daf-16* represents fold changes of neuronal *bet-1* (*rgef-1p::bet-1*) grown on *daf-16* RNAi against an N2 control grown on *daf-16* RNAi. *hsf-1* represents fold changes of for neuronal *bet-1* (*rgef-1p::bet-1*) grown on *hsf-1* RNAi against an N2 control grown on *hsf-1* RNAi. Warmer colors indicate increased expression and cooler colors indicate decreased expression. This is the complete list of genes compared to a filtered gene list in **Fig. 8E**. See **Supplemental Table S4** for all raw gene counts for heat map.

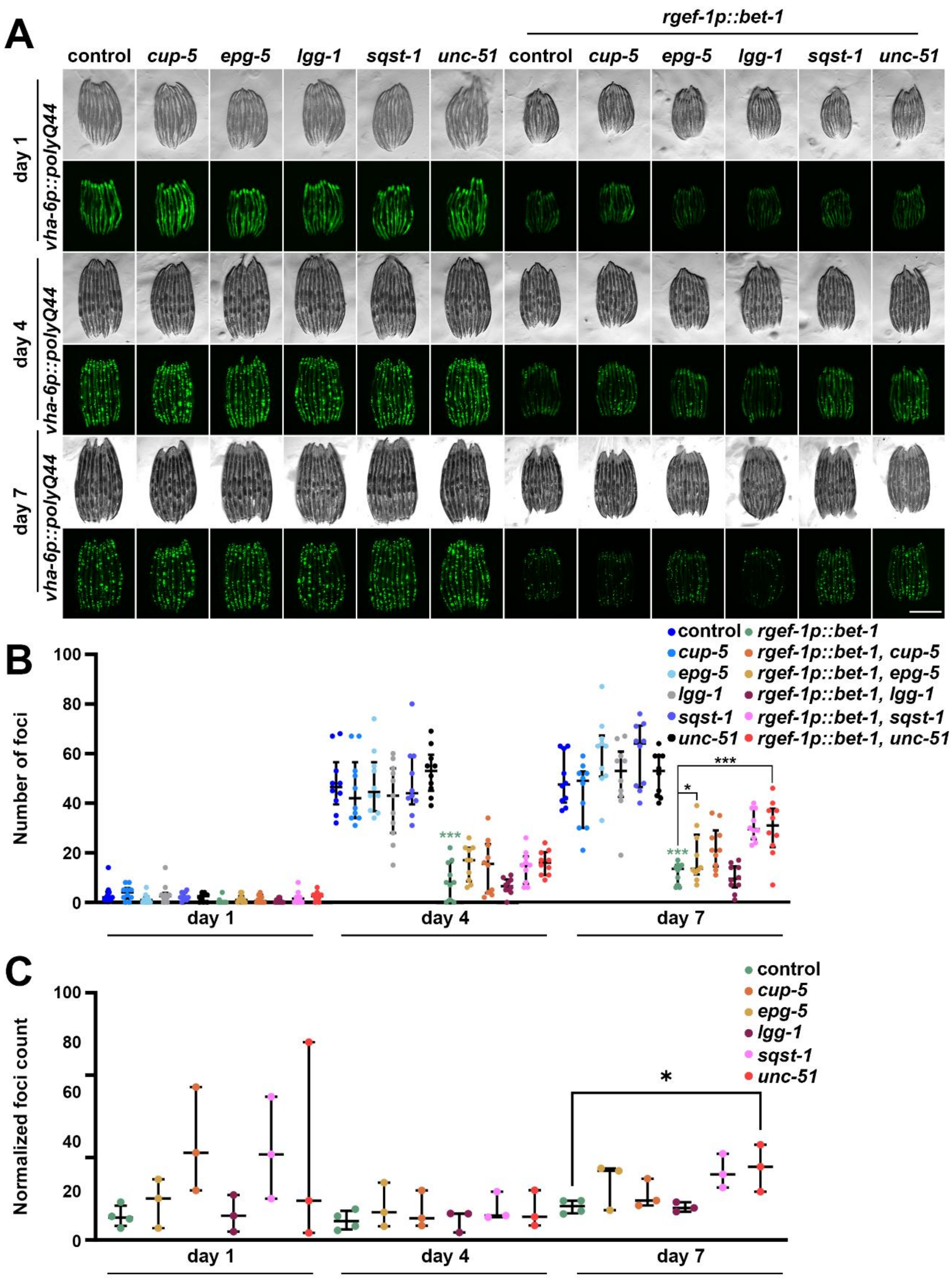

Supplemental Fig. S5. Neuronal *bet-1* reduces intestinal polyQ foci.
(A) Representative fluorescent images of day 1, 4, and 7 adult animals expressing *vha-6p*::*polyQ44::YFP* grown on EV (control), *bec-1, cup-5, lgg-1, unc-51,* or *sqst-1* RNAi bacteria

from L1. Data is representative of 3 independent replicates. Scale bar is 500 μm. **(B)** Quantification of A is presented as the number of polQ foci present in each animal. Lines represent the median and interquartile range. Each dot represents a single animal. Data is representative of 3 independent replicates. Green *** = $p < 0.001$ vs. wild-type control (N2, EV). Black * = $p < 0.05$; *** = $p < 0.001$ of RNAi condition vs. neuronal *bet-1*, EV control. **(C)** Quantification in (B) is represented as the ratio of polyQ foci in neuronal *bet-1* animals relative to the matched N2 control condition for each RNAi treatment. Ratios were calculated by dividing the mean polyQ foci number in the *rgef-1p::bet-1* group by the mean polyQ foci number in the corresponding N2 group within the same RNAi condition. Each dot represents a single animal. Lines represent the median and interquartile range. Data is compiled from 3 independent replicates. **** = $p < 0.0001$; *** = $p < 0.001$; ** = $p < 0.01$; * = $p < 0.05$; ns = non-significant, $p > 0.05$ by two-way ANOVA.

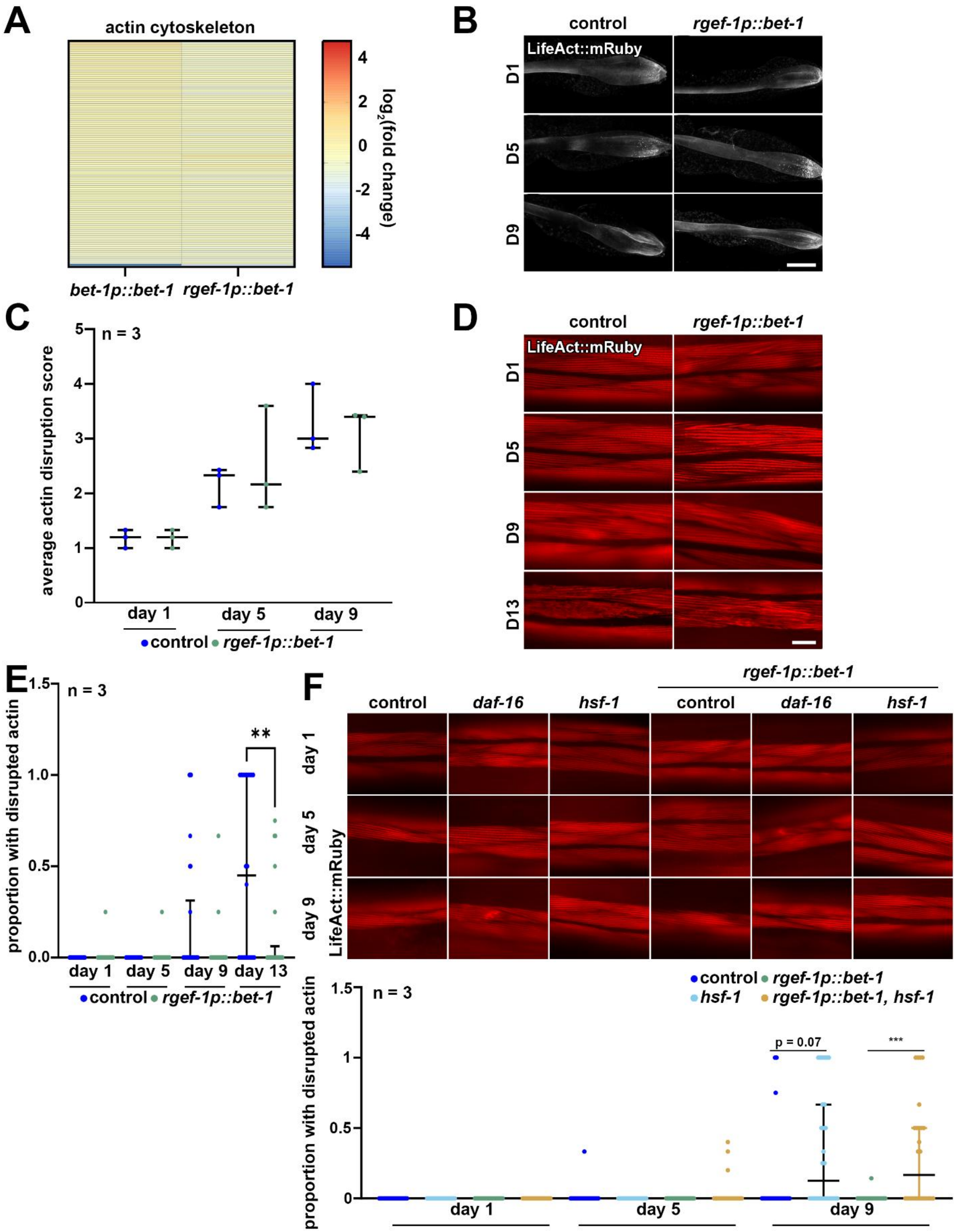

Supplemental Fig. S6. Neuronal *bet-1* mildly increases actin stability in the muscle, dependent on *hsf-1*.

(A) Heat map of actin gene expression (GO:0015629) showing *bet-1p::bet-1* and *rgef-1p::bet-1* animals compared to *glp-4(bn2)* control worms. Warmer colors indicate increased expression and cooler colors indicate decreased expression. See **Supplemental Table S4** for all raw gene counts for heat map. **(B)** Representative images of day 1, 5, and 9 adult animals expressing *gly-19p*::*LifeAct::mRuby* grown on EV bacteria from L1. Data is representative of 3 independent replicates. Scale bar is 10 µm. **(C)** Quantification of B measured as percentage of animals that have disrupted actin morphology per biological replicate. Each dot represents one percentage replicate across 3 biological replicates. **(D)** Representative images of day 1, 5, and 9 adult animals expressing *myo-3p*::*LifeAct::mRuby* grown on EV bacteria from L1. Data is representative of 3 independent replicates. Scale bar is 10 µm. **(E)** Quantification of D measured as the proportion of muscle cells with disrupted actin filaments in each animal. Each dot represents one animal. Lines represent the median and interquartile range. Data is representative of 3 independent replicates. **(F)** Representative images of day 1, 5, and 9 adult animals expressing *myo-3p*::*LifeAct::mRuby* grown on EV, *daf-16*, or *hsf-1* bacteria from L1. Data is representative of 3 independent replicates. Scale bar is 10 µm. **(G)** Quantification of F measured as the of muscle cells with disrupted actin filaments in each animal. Each dot represents one animal. Lines represent the median and interquartile range. Data is representative of 3 independent replicates.

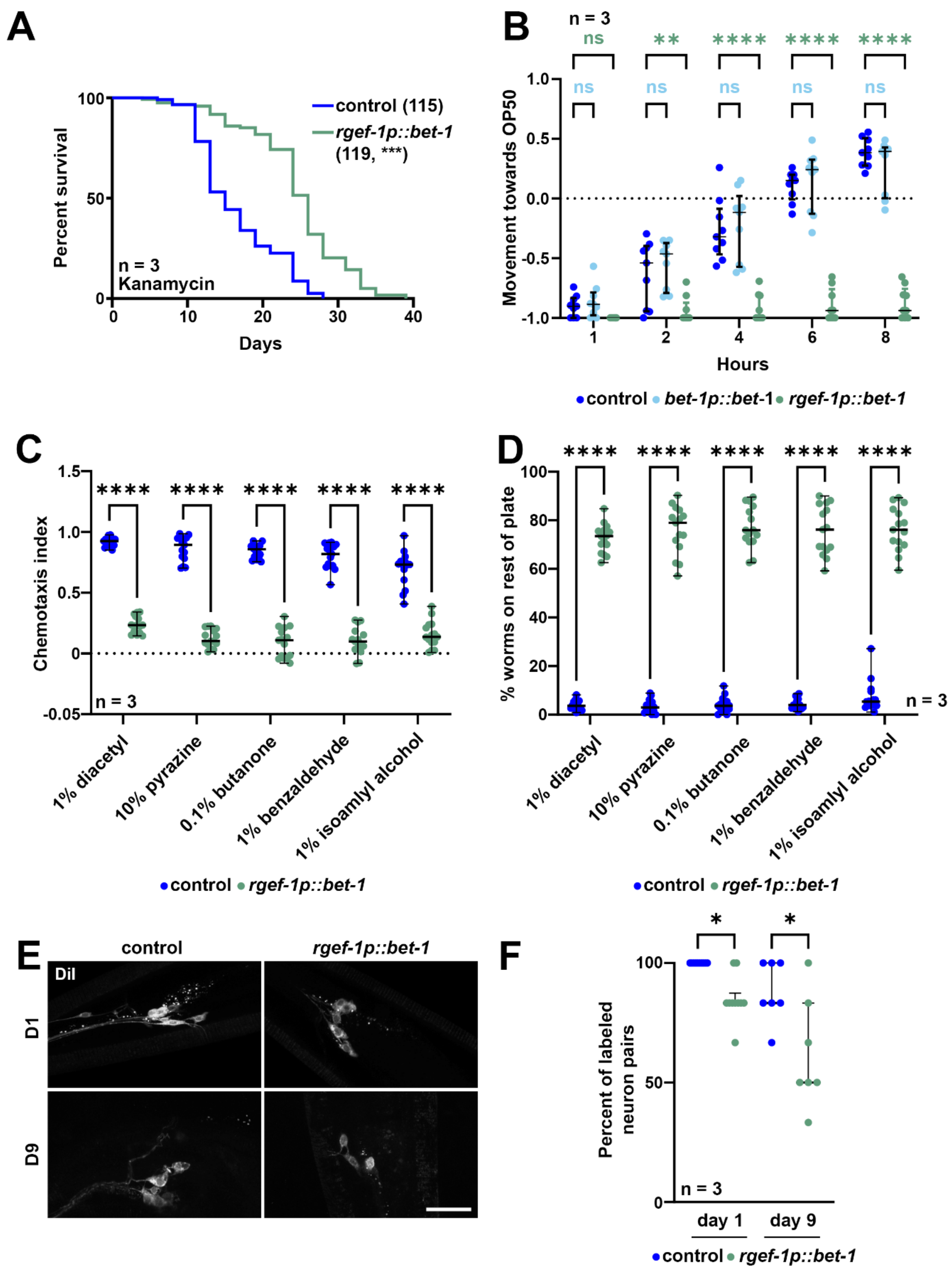

Supplemental Fig. S7. Neuronal *bet-1* animals display neuronal defects.
(A) Lifespans of N2 control and neuronal *bet-1* (*rgef-1p::bet-1*) animals grown on live or kanamycin-killed EV. *** = p < 0.001; n.s. = not significant, p > 0.05 using Log-rank testing. See

Supplemental Table S1 for complete lifespan statistics. **(B)** Relative movement of N2 control (blue), whole animal *bet-1* (*bet-1p::bet-1*, light blue), and neuronal *bet-1* (*rgef-1p::bet-1*, green) day 1 adult animals in a “forced” food choice assay towards a PA14 pathogen containing OP50 bacterial lawn. **** = $p < 0.0001$; *** = $p < 0.001$; ** = $p < 0.01$; ns = non-significant, $p > 0.05$ two-way ANOVA. **(C)** Relative chemotaxis index of N2 control (blue) and neuronal *bet-1* (*rgef-1p::bet-1*, green) day 1 adult animals against several chemoattractants. **(D)** % of animals not on the chemoattractant spot are calculated as “% of worms on rest of plate” at the end of the chemotaxis assay. Data is pooled across 3 independent biological replicates. **** = $p < 0.0001$ using 2way ANOVA with Bonferroni’s multiple comparison tests. **(E)** Representative images of DiI-labeled sensory neurons in N2 control (blue) and neuronal *bet-1* (*rgef-1p::bet-1*, green) animals at days 1 and 9 of adulthood. **(F)** Quantification of sensory neuron dye filling in N2 control (blue) and neuronal *bet-1* (*rgef-1p::bet-1*, green) animals at days 1 and 9 of adulthood. Dye filling was quantified as the percentage of the six amphid neuron pairs showing detectable DiI labeling per animal. Each dot represents one animal, and lines indicate the median and interquartile range. Data is representative of 3 independent replicates. **** = $p < 0.0001$; *** = $p < 0.001$; ** = $p < 0.01$; * = $p < 0.05$; ns = non-significant, $p > 0.05$ by Mann-Whitney U test.

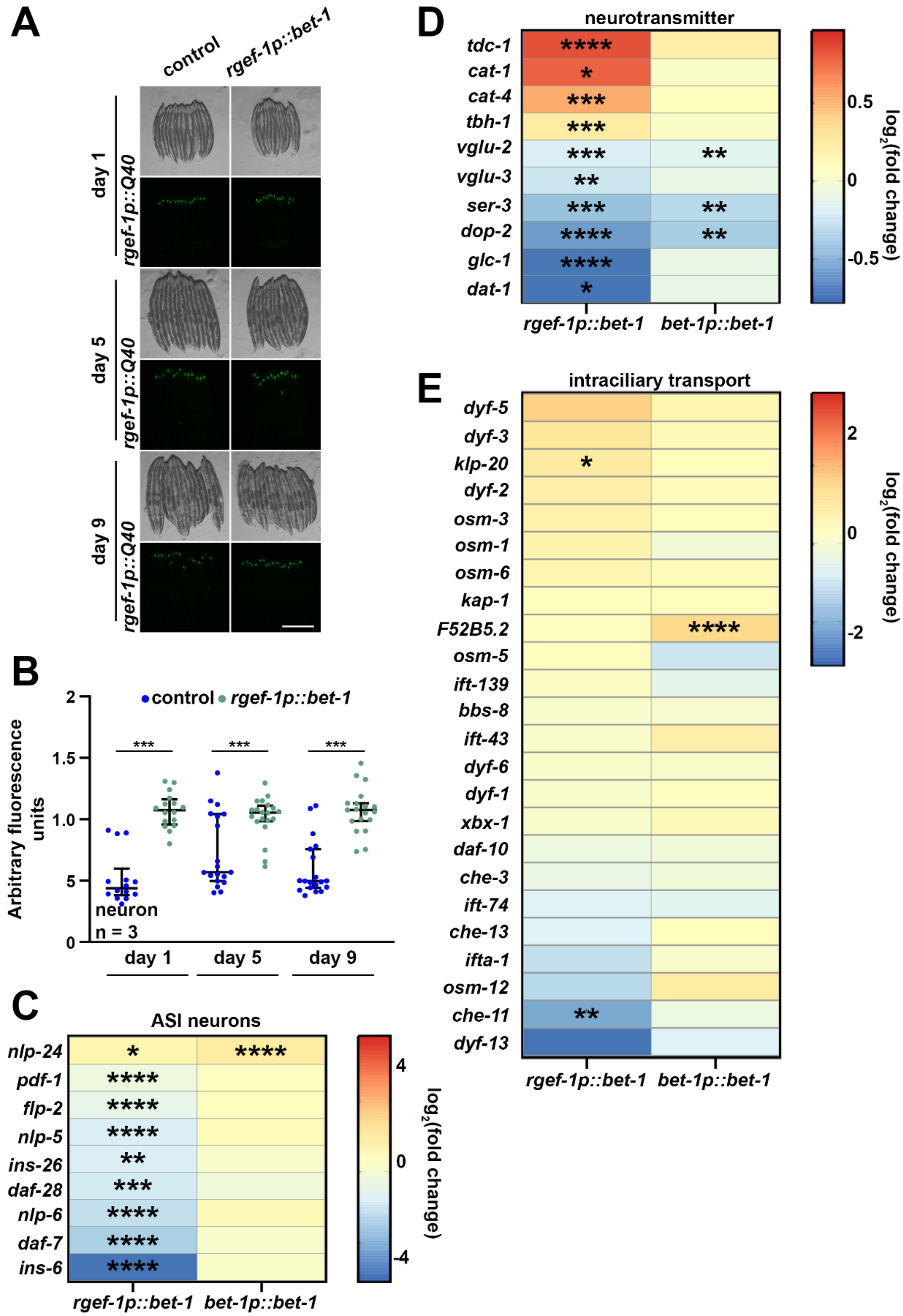

Supplemental Fig. S8. Neuronal *bet-1* animals display increased neuronal polyQ levels. **(A)** Representative fluorescent images of control and neuronal *bet-1* (*rgef-1p::bet-1*) day 1, 5, and 9 adult animals expressing *rgef-1p::polyQ40::YFP* grown on EV. Data is representative of 3

independent replicates. Scale bar is 500 µm. **(B)** Quantification of A is presented as the integrated fluorescence intensity in each animal. Lines represent the median and interquartile range. Each dot represents a single animal. Data is representative of 3 independent replicates. Green *** = $p < 0.001$ vs. wild-type control (N2, EV). *** = $p < 0.001$ vs. N2 control. **(C)** Heat map of differentially expressed ($p < 0.05$) ASI neuron-expressed peptide signaling genes showing expression changes in *bet-1p::bet-1* and *rgef-1p::bet-1* animals compared to *glp-4(bn2)* control worms. **(D)** Heat map of differentially expressed (p-value < 0.05) neurotransmitter genes showing expression changes in *bet-1p::bet-1* and *rgef-1p::bet-1* animals compared to *glp-4(bn2)* control worms. **(E)** Heat map of IFT/IFT-associated genes (IntraciliaryTransport GO:0042073) showing expression changes in *bet-1p::bet-1* and *rgef-1p::bet-1* animals compared to *glp-4(bn2)* control worms. Warmer colors indicate increased expression and cooler colors indicate decreased expression. **** = $p < 0.0001$; *** = $p < 0.001$; ** = $p < 0.01$; * = $p < 0.05$. See Supplemental Table S4 for all raw gene counts for heat map.

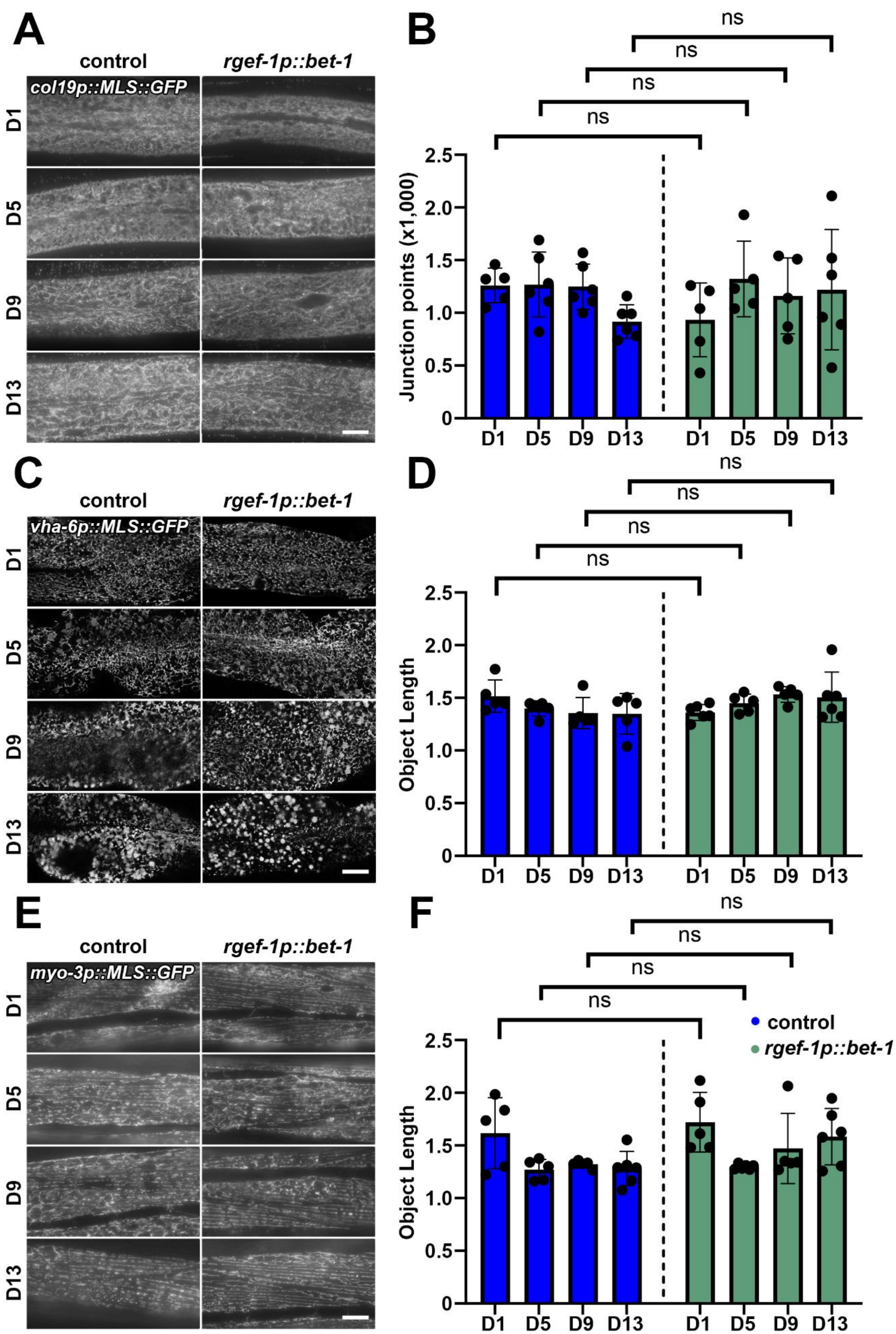
A
control
rgef-1p::bet-1
col19p::MLS::GFP
D1
D5
D9
D13
B
ns
ns
ns
ns
Junction points (x1,000)
2.5
2.0
1.5
1.0
0.5
0
D1
D5
D9
D13
D1
D5
D9
D13
C
control
rgef-1p::bet-1
vha-6p::MLS::GFP
D1
D5
D9
D13
D
ns
ns
ns
ns
Object Length
2.5
2.0
1.5
1.0
0.5
0
D1
D5
D9
D13
D1
D5
D9
D13
E
control
rgef-1p::bet-1
myo-3p::MLS::GFP
D1
D5
D9
D13
F
ns
ns
ns
ns
control
rgef-1p::bet-1
Object Length
2.5
2.0
1.5
1.0
0.5
0
D1
D5
D9
D13
D1
D5
D9
D13

Supplemental Fig. S9. Neuronal *bet-1* does not impact mitochondrial morphology. (A) Imaging of hypodermal mitochondria (*col-19p::MLS::GFP*) in wild-type control (blue) and neuronal *bet-1* (*rgef-1p::bet-1*, green) animals at day 1, 5, 9, and 13 of adulthood. Scale bar is 10 µm. **(B)** Quantification of (A) using mitoMAPR. Junction points is used as a representative measurement for hypodermal mitochondria as previously described(Kim et al. 2025b) and all measurements are available in **Supplemental Table S6**. ns = $p > 0.05$ using Wilcoxin testing. **(C)** Imaging of intestinal mitochondria (*vha-6p::MLS::GFP*) in wild-type control and neuronal *bet-1* (*rgef-1p::bet-1*) animals at day 1, 5, 9, and 13 of adulthood. Scale bar is 10 µm. **(D)** Quantification of (C) using mitoMAPR. Object length is used as a representative measurement for intestinal mitochondria as previously described and all measurements are available in **Supplemental Table S6**. ns = $p > 0.05$ using Wilcoxin testing. **(E)** Imaging of muscle mitochondria (*myo-3p::MLS::GFP*) in wild-type control and neuronal *bet-1* (*rgef-1p::bet-1*) animals at day 1, 5, 9, and 13 of adulthood. Scale bar is 10 µm. **(F)** Quantification of (E) using mitoMAPR. Object length is used as a representative measurement for hypodermal mitochondria as previously described and all measurements are available in **Supplemental Table S6**. ns = $p > 0.05$ using Wilcoxin testing.

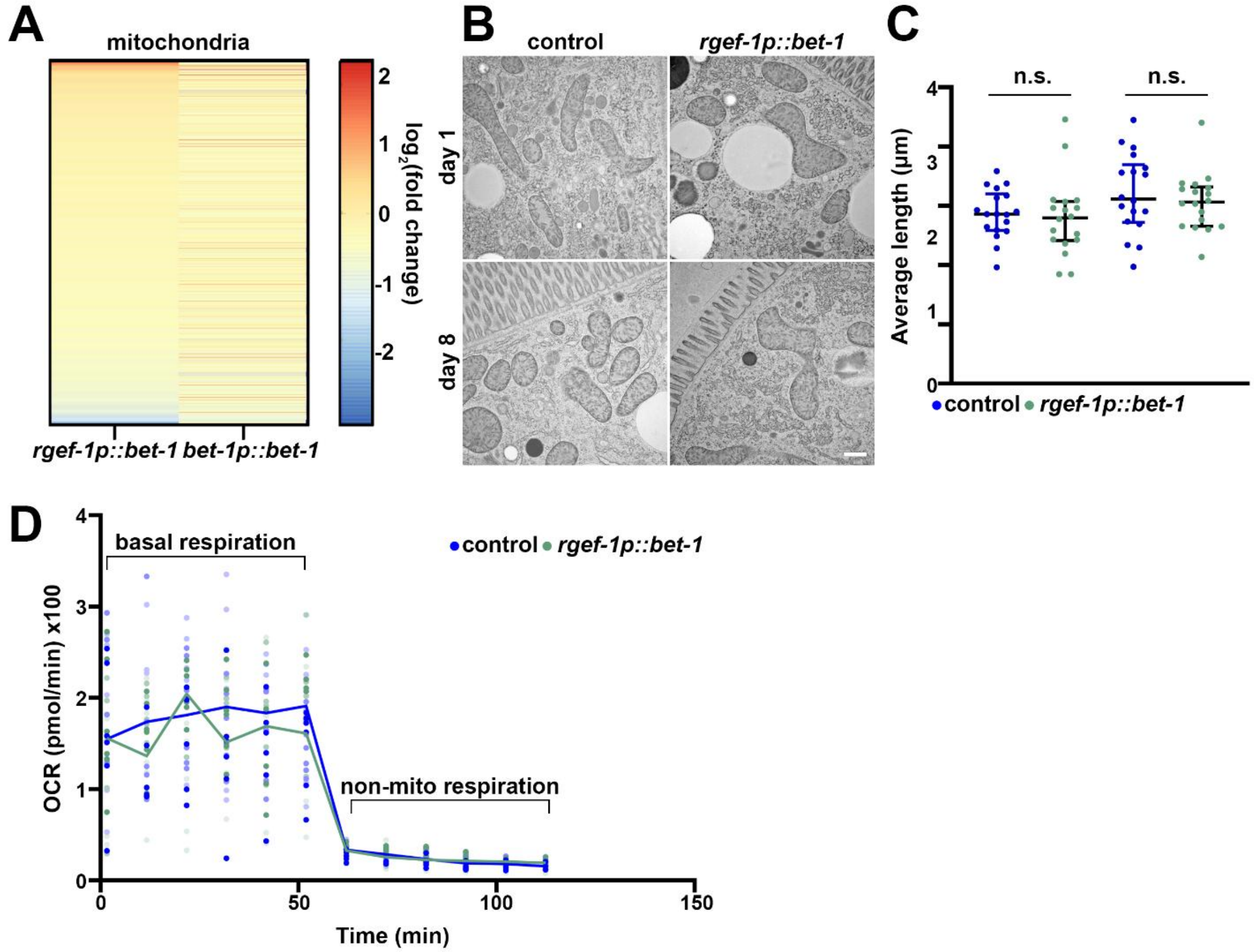

Supplemental Fig. S10. Neuronal *bet-1* does not impact mitochondrial structure or mitochondrial respiration. (A) Heat map of mitochondria gene expression (GO:0005739) showing *bet-1p::bet-1* and *rgef-1p::bet-1* animals compared to *glp-4(bn2)* control worms. Warmer colors indicate increased expression and cooler colors indicate decreased expression. See **Supplemental Table S4** for all raw gene counts for heat map. **(B)** Electron microscopy was performed in the intestine of wildtype control and neuronal *bet-1* animals grown on EV RNAi from L1 at day 1 and day 8 of adulthood. White arrowheads indicate autophagosome-like structures. Scale bar is 500 µm. **(C)** Quantification of B measured as average length of mitochondria in microns. Each dot represents the average of 10 mitochondria that were measured in each EM image. 9 images of 2 independent worms were quantified for a total of 18 images per condition. **(D)** Respiration of day 1 adult animals grown on EV from L1. N = 6 wells, each with 10 worms, over n = 3 biological replicates (18 wells total per condition) measuring as oxygen consumption rate (OCR) using a Seahorse XF96 cell culture microplate and XFE96 Analyzer. OCR was measured every ~6 min with 3 minutes mixing, 2 minutes wait, and 2 minutes measuring over 6 time points for basal respiration. Then sodium azide was added to 50 mM concentration to measure non-mitochondrial (non-mito) respiration. No statistical differences were measured at any time points using non-parametric Kruskal Wallis testing ($p > 0.05$). Dots represent individual wells and line represents median across all 18 wells. Data is pooled across 3 biological replicates.

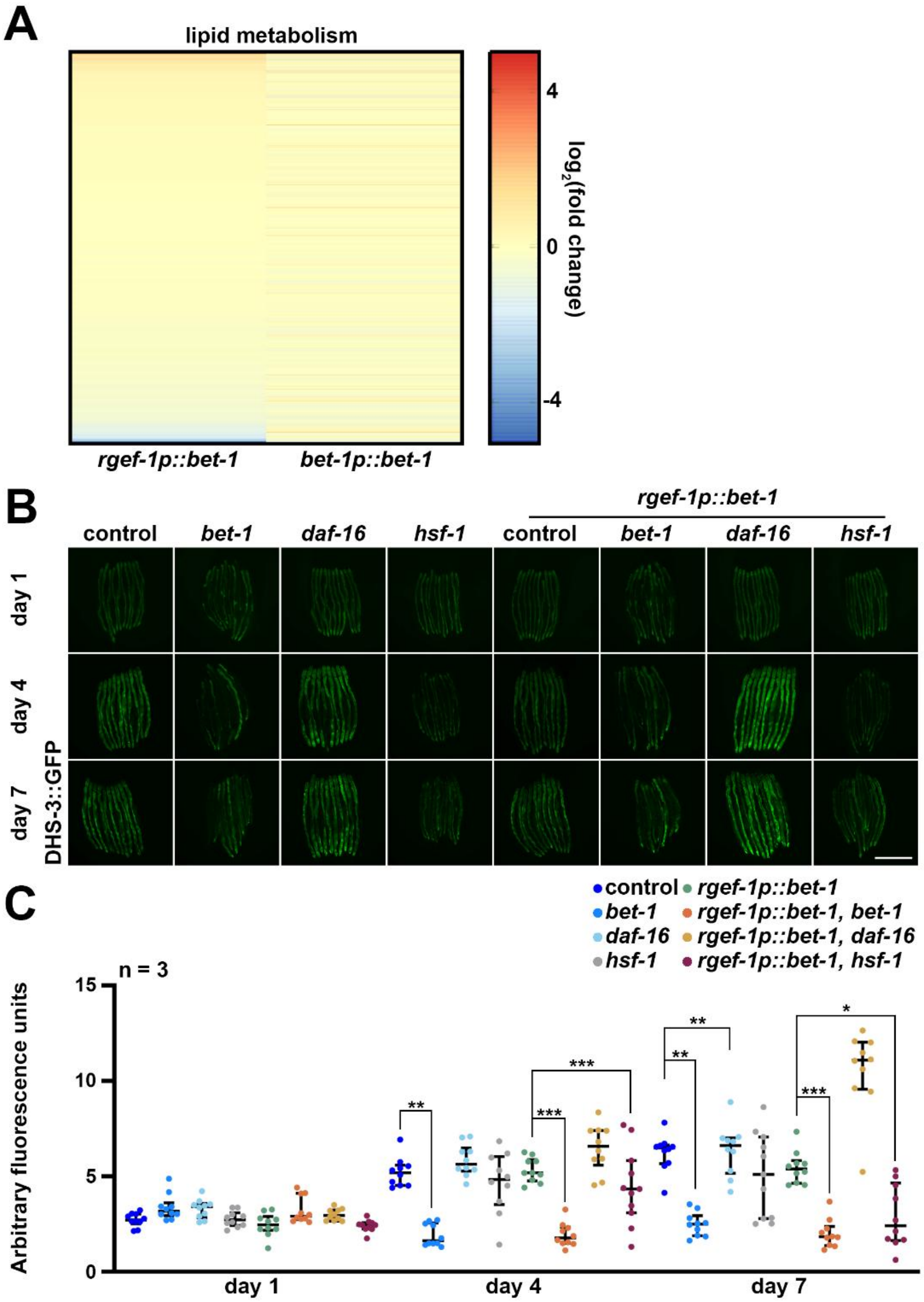

Supplemental Fig. S11. Neuronal *bet-1* does not alter lipid droplet levels. (A) Heat map of lipid metabolism gene expression (GO:0006629) showing *bet-1p::bet-1* and *rgef-1p::bet-1* animals compared to *glp-4(bn2)* control worms. Warmer colors indicate increased expression,

and cooler colors indicate decreased expression. See **Supplemental Table S4** for all raw gene counts for heat map. **(B)** Representative fluorescent images of day 1, 4, and 7 adult animals expressing *DHS-3::GFP* grown on EV (control), *bet-1, daf-16,* or *hsf-1* RNAi bacteria from L1. Data is representative of 3 independent replicates. Scale bar is 500 µm. **(C)** Quantification of B is presented as the integrated fluorescence intensity in each animal. Lines represent the median and interquartile range. Each dot represents a single animal. Data is representative of 3 independent replicates. * = $p < 0.05$; ** = $p < 0.01$; *** = $p < 0.001$.

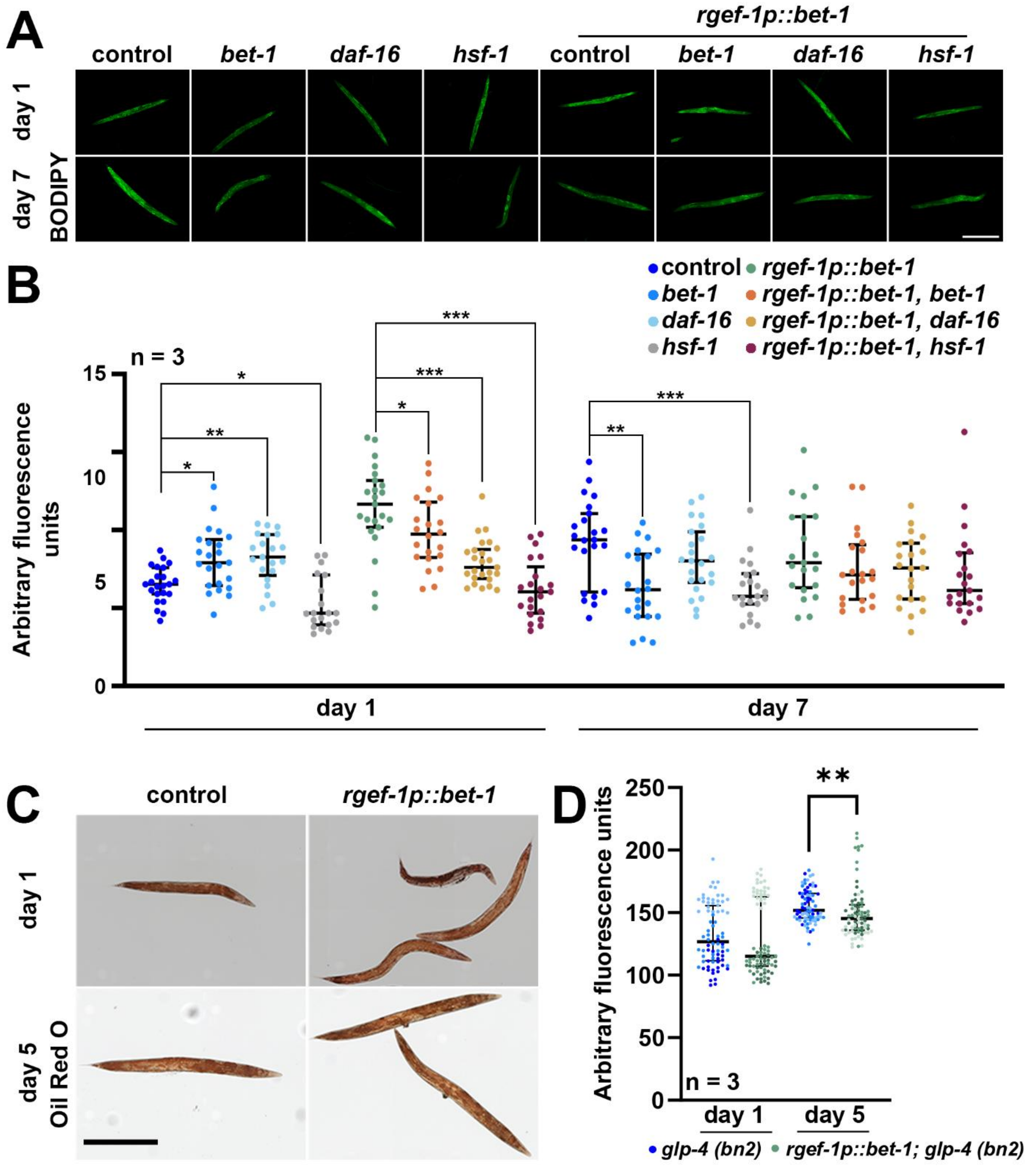

Supplemental Fig. S12. Neuronal *bet-1* has variable changes in lipids as measured by BODIPY and Oil Red O. (A) Representative images and quantification of BODIPY-stained neutral lipids in N2 and neuronal *bet-1* (*rgef-1p::bet-1*) animals under the indicated RNAi conditions. **(B)** Quantification of BODIPY-stained neutral lipids in N2 and neuronal *bet-1* (*rgef-1p::bet-1*) animals under the indicated RNAi conditions. Lipid levels were quantified as integrated fluorescence units per animal. Each dot represents one animal, and lines indicate the median and interquartile range. Data is representative of 3 independent replicates. **** = p < 0.0001; *** = p < 0.001; ** = p < 0.01; * = p < 0.05, p > 0.05 by two-way ANOVA. **(C)**

Representative images of Oil Red O-stained neutral lipids in *glp-4(bn2)* animals with and without neuronal *bet-1* overexpression(*rgef-1p::bet-1*). Day 1 and day 5 data are contrasted separately for clarity. **(D)** Quantification of (C). Lipid levels were quantified by drawing an ROI around each individual worm and measuring the red intensity using an RGB color camera scale. Data is presented as integrated red density normalized to ROI (i.e., worm) area. Each dot represents one animal, and lines indicate the median and interquartile range. Data is a superplot of 3 independent replicates. **** = $p < 0.0001$; *** = $p < 0.001$; ** = $p < 0.01$; * = $p < 0.05$, $p > 0.05$ by Mann-Whitney U test.

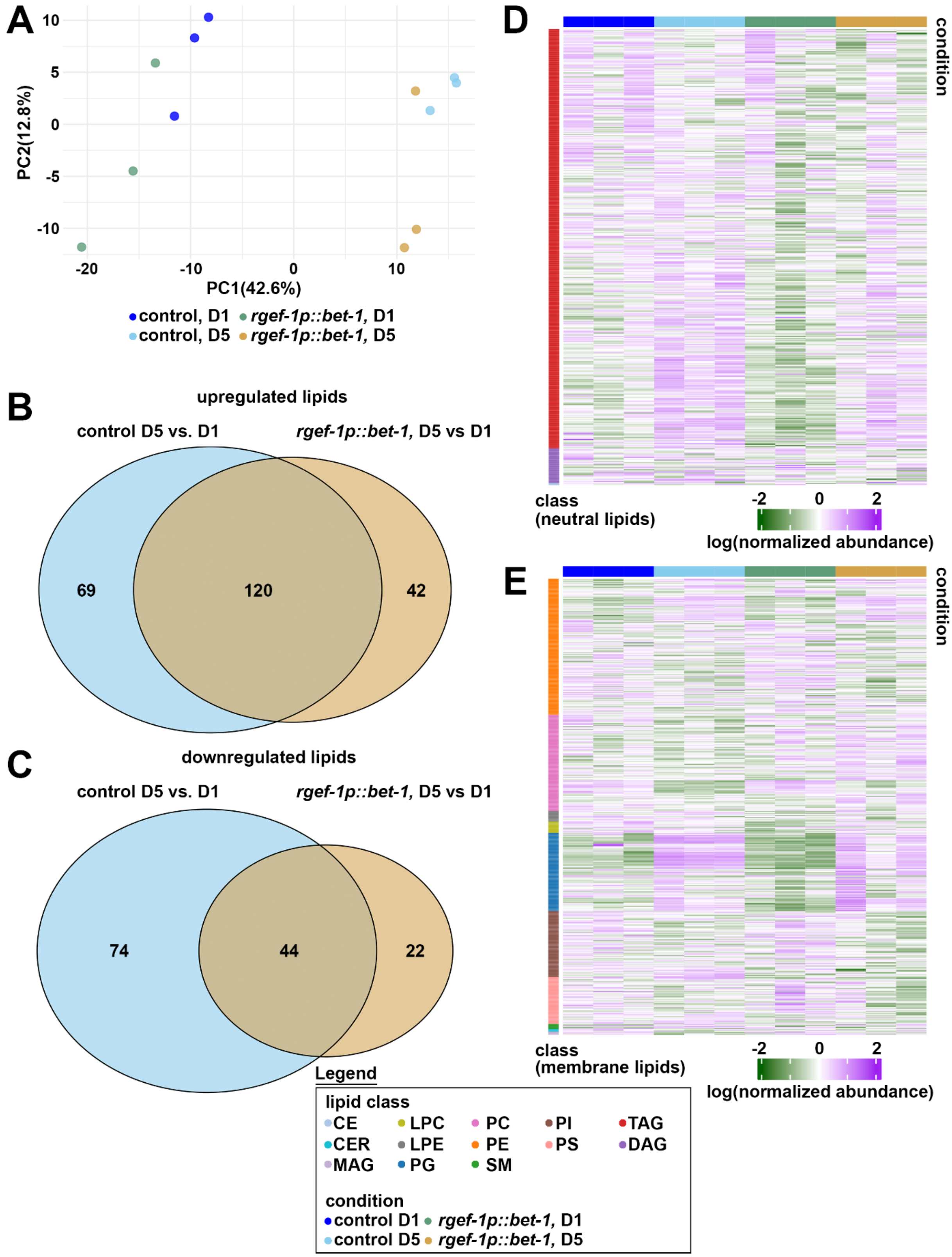

Supplemental Fig. S13. Neuronal *bet-1* results in minor changes in lipids with age. (A) PCA plots of all lipidomics conditions. **(B)** Venn diagram displaying overlap of lipids that increase in abundance with age. **(C)** Venn diagram displaying overlap of lipids that decrease in

abundance with age. **(D)** Spearman correlation plot of neutral lipids. Green indicates lower abundance and purple indicates higher abundance. **(E)** Spearman correlation plot of membrane lipids. Green indicates lower abundance and purple indicates higher abundance. Plot for all lipids is available in **Fig. 8**. All raw values are available in **Supplemental Table S5.**

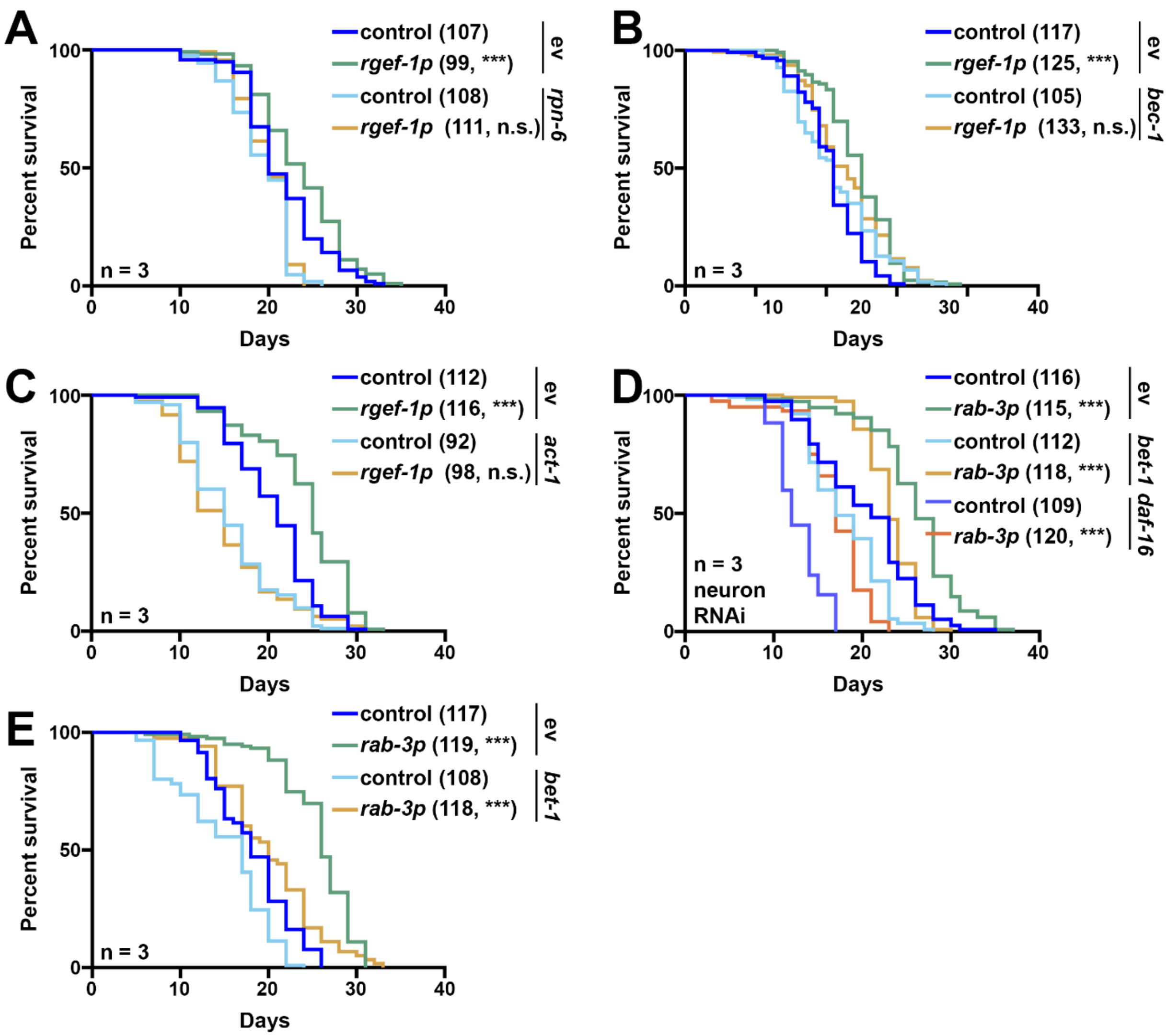

Supplemental Fig. S14. Neuronal *hsf-1* animals do not require *bet-1* for lifespan extension. (A) Lifespans of N2 control and neuronal *bet-1* (*rgef-1p::bet-1*) animals grown on EV or *rpn-6.1* RNAi from L1. *** = p < 0.001; n.s. = not significant, p > 0.05 using Log-rank testing. **(B)** Lifespans of N2 control and neuronal *bet-1* (*rgef-1p::bet-1*) animals grown on EV or *bec-1* RNAi from L1. *** = p < 0.001; n.s. = not significant, p > 0.05 using Log-rank testing. See **(C)** Lifespans of N2 control and neuronal *bet-1* (*rgef-1p*) animals grown on EV or 10% *act-1* RNAi (diluted with 90% EV) from L1. *** = p < 0.001; n.s. = not significant p > 0.05 using Log Rank testing. **(D)** Lifespans of MAH677 (neuron-specific RNAi) control and MAH677 with neuronal *hsf-1* overexpression (*rab-3p*) grown on EV, *bet-1*, or *daf-16* RNAi from L1. **(E)** Lifespans of wildtype N2 control and neuronal *hsf-1* overexpression (*rab-3p*) grown on EV or *bet-1* RNAi from L1. See **Supplemental Table S1** for complete lifespan statistics.

Supplemental Table Legends

Supplemental Table S1. Lifespan Statistics

Supplemental Table S2. All Significant DEGs

Supplemental Table S3. All GO Terms

Supplemental Table S4. Heat Maps Gene list updated

Supplemental Table S5. Lipidomics data

Supplemental Table S6. All mitoMAPR Quantification

Supplemental Table S7. Strains used in this study

Supplemental Table S8. All raw data files (not included in Tables S1-S6)
